# Supplementary material for: Aberrant spliceosome activity via elevated intron retention and upregulation and phosphorylation of SF3B1 in chronic lymphocytic leukemia
Source: Mol Ther Nucleic Acids. 2024 Apr 26;35(2):102202. doi: 10.1016/j.omtn.2024.102202 (PMC11154714; doi:10.1016/j.omtn.2024.102202)
Supplement: Document S1. Figures S1–S13 and Tables S3–S5 [file mmc1.pdf]

OMTN, Volume *35*

Supplemental information

Aberrant spliceosome activity via elevated intron retention and upregulation and phosphorylation of SF3B1 in chronic lymphocytic leukemia

Manoj Kumar Kashyap, Hiren Karathia, Deepak Kumar, Roberto Vera Alvarez, Jose Vicente Forero-Forero, Eider Moreno, Juliana Velez Lujan, Carlos Ivan Amaya-Chanaga, Newton Medeiros Vidal, Zhe Yu, Emanuela M. Ghia, Paula A. Lengerke-Diaz, Daniel Achinko, Michael Y. Choi, Laura Z. Rassenti, Leonardo Mariño-Ramírez, Stephen M. Mount, Sridhar Hannenhalli, Thomas J. Kipps, and Januario E. Castro

Supplemental Material

Detailed information required to perform the intron/exon quantification as we did in this work

Our assumption was if the expression of a distinct transcript *i* in sample *j* differs from the expected value for an experimental condition (i.e., normal or cancer), the values *μijl* for all of the counting bins *l* of transcript *i* will deviate from the values expected for condition in normal or CLL by the same factor. Hence, we consider the bin (*l*) as length of exonic and intronic region of a transcript defined in standard annotation of hg19 NCBI Refseq.

Formulas used to operate the *in-house* program designed to calculate TPM values for every exonic and intronic region of the expressed transcripts

The TPM calculations were performed separately for every exon, intron and transcript in each sample (CLL and NBC). To capture single value of intron and exon per transcript in a sample, we first calculated $\max(\forall TPM_{exon})$ and $\max(\forall TPM_{intron})$ for each transcript and then took $mean[\max(\forall TPM_{exon})]$ and $mean[\max(\forall TPM_{intron})]$ across NBC and CLL samples, which we termed respectively for intron-usage and exon-usage as defined as $TPM\_NBC\_Intronic$, TPM_NBC_Exonic, $TPM\_CLL\_Intronic$ and TPM_CLL_Exonic (Equation S1, S2, and S3). [1]

$$TPM_{i=} \frac{r_i * 10^6}{l_i \sum_{j=1}^{N} \frac{r_i}{l_j}}$$ **Equation - S1**

$$TPMexon\_i = 10^6 * \frac{\frac{NEi}{LEi}}{\sum_{j=0}^{j=n} \left(\frac{NEj}{LEj}\right)}$$ **Equation - S2**

$$TPMintron\_i = 10^6 * \frac{\frac{NIi}{LIi}}{\sum_{j=0}^{j=n} \left(\frac{NIj}{LIj}\right)}$$ **Equation - S3**

where *N*=Transcript, *NE*=Exonic region, *NI*=Intronic region, *LE*=Length of the exonic region and *LI*=Length of intronic region.

In order to quantify relative intron-usage and exon-usage for each transcript in NBC and CLL, we took two combinations of ratios. Firstly, two separated ratios of $TPM\_Intronic$ and TPM_Exonic in between CLL and NBC; we call these ratios as Log2_intronic and Log2_exonic respectively (Equation S4 & S5). Secondly, the same way the two separate ratios; one for $TPM\_Intronic$ and TPM_Exonic in CLL and the same in NBC.

$$Log2Intronic_i = log_2\left(\frac{TPM\_CLL\_Intronic_i}{TPM\_NBC\_Intronic_i}\right) \qquad \textbf{Equation – S4}$$

$$Log2Exonic_i = log_2\left(\frac{TPM\_CLL\_Exonic_i}{TPM\ NBC\ Exonic_i}\right) \qquad \textbf{Equation – S5}$$

Equations (S4 & S5) were used for comparing the similar entity between experimental conditions, which was CLL versus NBC cells in this case. By doing so, TPM value of the exonic regions can be related to the expression level of the isoform in the experiments, an isoform with a positive value of equation S4 can be read as more expression of those exonic or intronic regions in the CLL compared to NBC and vice versa if the value is negative.

Supplemental Tables

* **Table S1.** List of Chronic Lymphocytic Leukemia Patients and Normal Subject Used in the study

* **Table S2.** List of transcripts with IR in Chronic Lymphocytic Leukemia (CLL)-B Cells and Normal B Cells (NBCs)

* Please refer to the "**Supplemental Videos and Spreadsheets**"

Table S3. List of primers for detection of SF3B1 mutations in CLL Samples[2]

SF3B1	Primer Direction	Primer Sequence	PCR Product (BPs)
Exon 14	Forward	5’ TCTGTTTATGGAATTGATTATGGAA 3’	424
Exon 14	Reverse	3’ GGGCAACATAGTAAGACCCTGT 5’	
Exon 15	Forward	5’ TTGGGGCATAGTTAAAACCTG 3’	209
Exon 15	Reverse	3’ AAATCAAAAGGTAATTGGTGGA 5’	

Table S4. *SF3B1* Mutational status of CLL patients used in RNA-seq Study

S. NO.	Sample ID	Type of *SF3B1* Mutation
1	CLL009	MUT
2	CLL019	MUT
3	CLL029	MUT
4	CLL053	MUT
5	CLL154	MUT
6	CLL156	MUT
7	CLL182	MUT
8	CLL197	MUT
9	CLL306	MUT

Table S5. Primers used for other genes validated using RT-PCR

Name of Molecule (Gene Symbol)	Refseq ID	Primers	Primer Sequence [5' -----→ 3']	Amplicon Size (bp)
THAP8	NM_152658	FP	GATGGGGAGAGCCAAGACTTC	213
		RP	GTTTGTGTCCCGGAAGAATGG	
PPP2R5B	NM_006244	FP (Set-1A)	ATTAGACTCGCTTTGGGATGC	104
		RP (Set-1A)	GCCCATAAGAACGGGAGGAAG	
		FP (Set-1B)	CATTAGACTCGCTTTGGGATGC	106
		RP (Set-1B)	GGCCCATAAGAACGGGAGGA	
PSTPIP1	NM_003978	FP	GTGGAAACCAAGACTGCCTCT	135
		RP	GGAGGATGGAGCATGACTGA	
PTPRJ	NM_001098503	FP	TTCAACCCGTGGACTTTGGT	177
		RP	TGCCCCCACGATGTTGATTT	
NUBP2	NM_001284502	FP	CCTGGCTCCGTGTTCTGATT	178
		RP	TCCATCAGGTGTCACTTGCC	
FP: Forward primer, RP: Reverse primer, THAP8: THAP domain containing 8, PPP2R5B: Protein phosphatase 2 regulatory subunit B'beta, PSTPIP1: Proline-serine-threonine phosphatase interacting protein 1, PTPRJ: *Protein tyrosine phosphatase receptor type J*, *NUMBP2*: NUBP iron-sulfur cluster assembly factor 2, cytosolic				

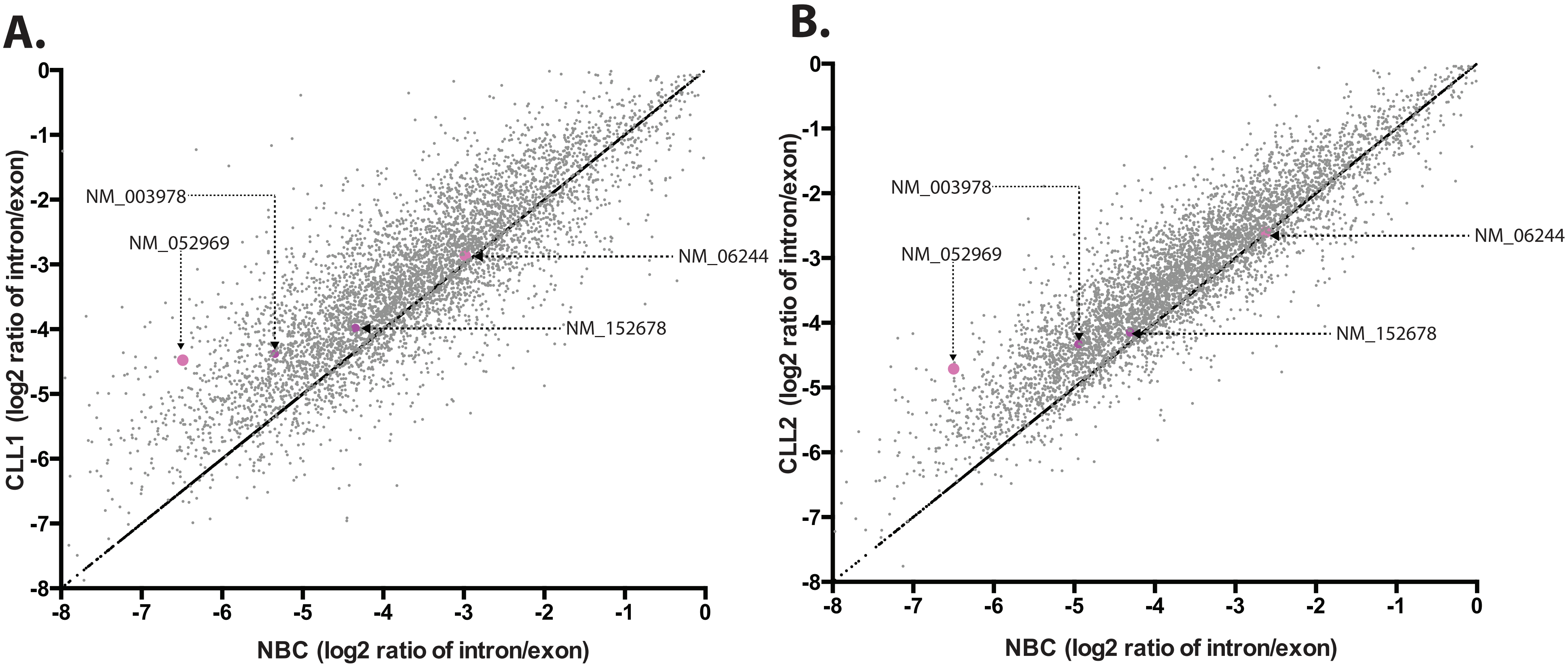

Figure S1: Intron/Exon ratio in CLL vs. NBCs

A. Scatter Plot

B. 2D Quadrant Distribution

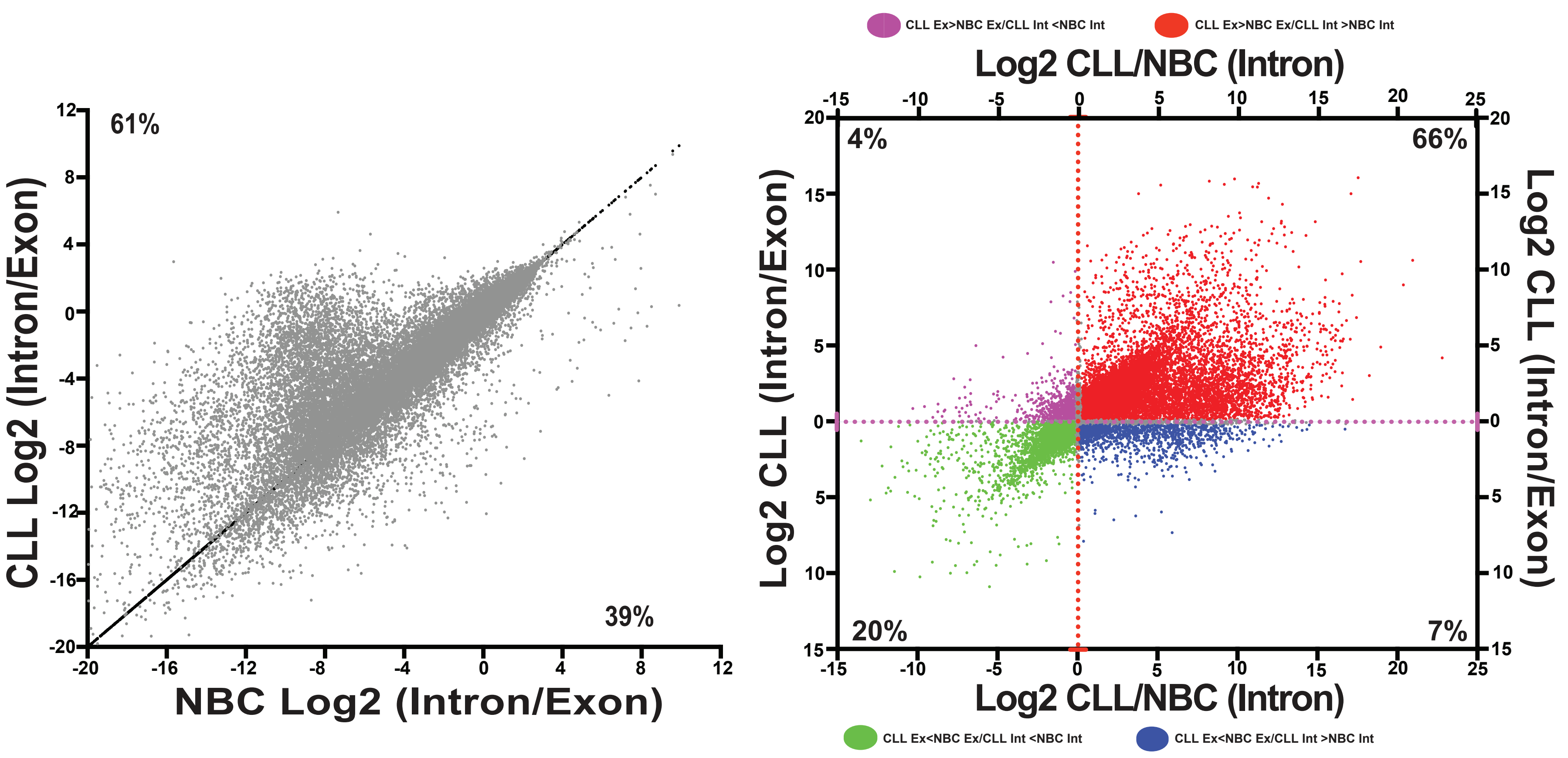

Figure S2. Global distribution of I/E Between CLL vs NBC

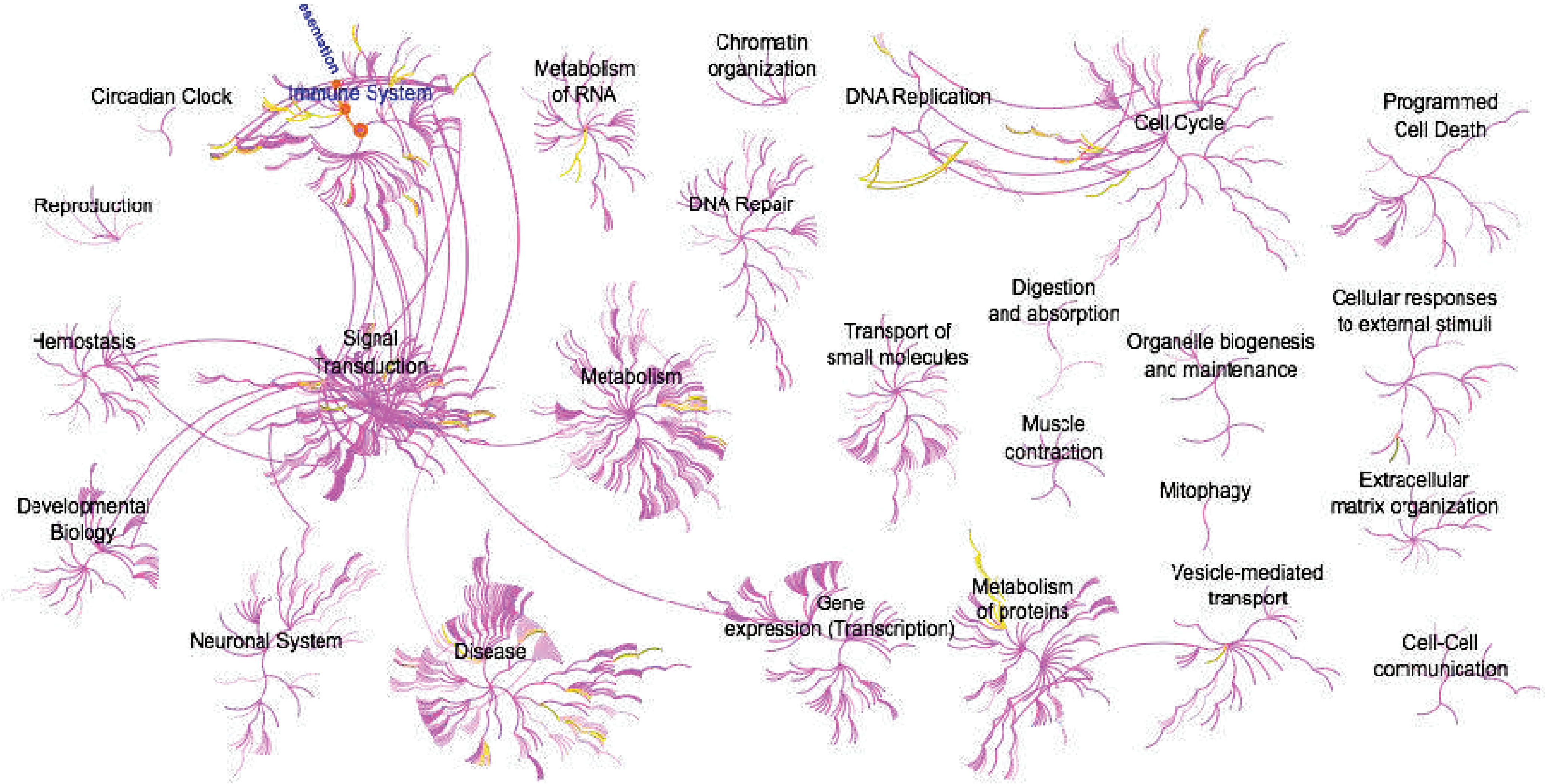

Figure S3. Emergence of major spliceosome pathwyas with higher intron retention possessing transcripts in Normal B Cells

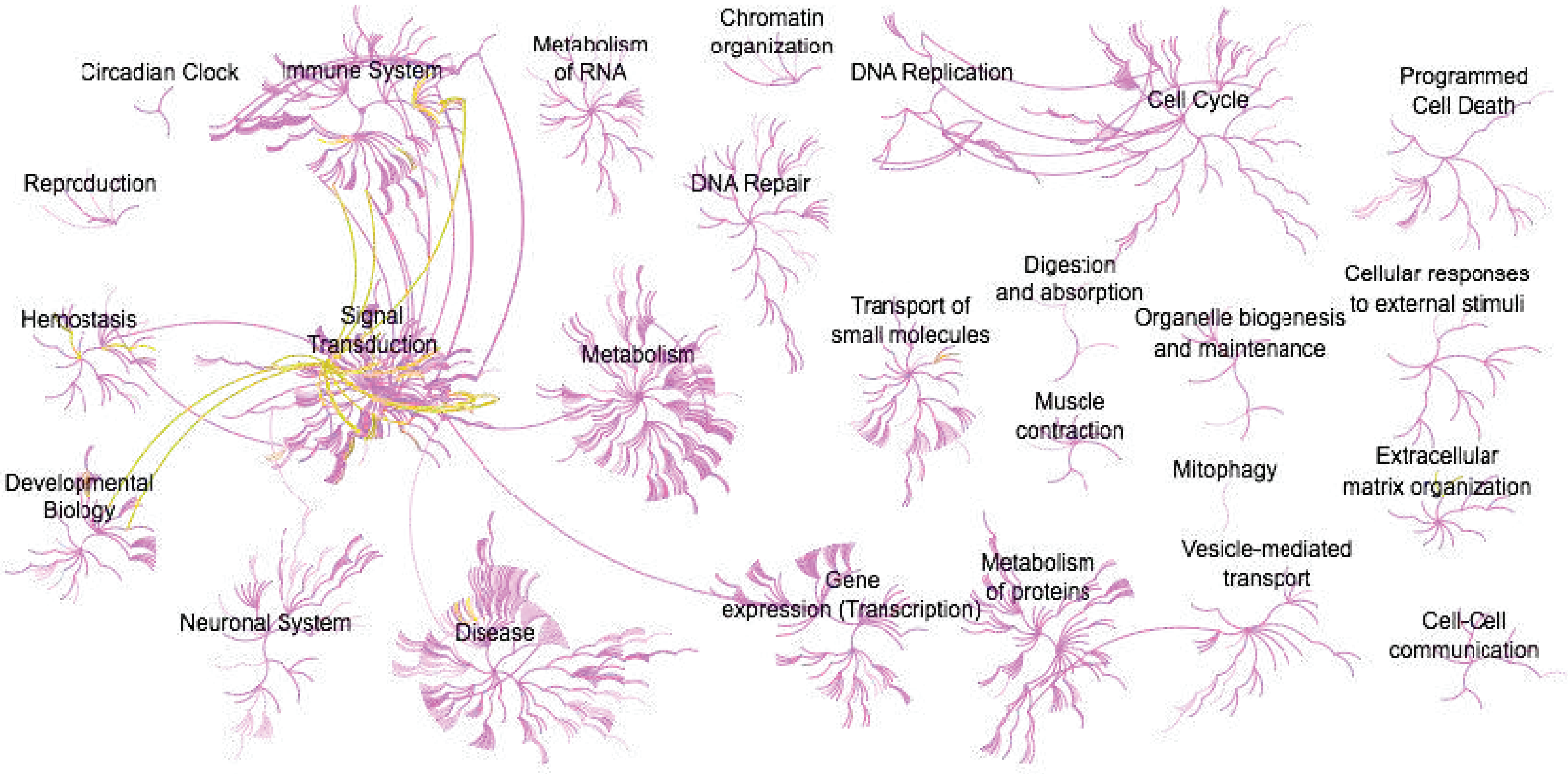

Figure S4. Emergence of major spliceosome pathwyas with higher intron retention possessing transcripts in CLL

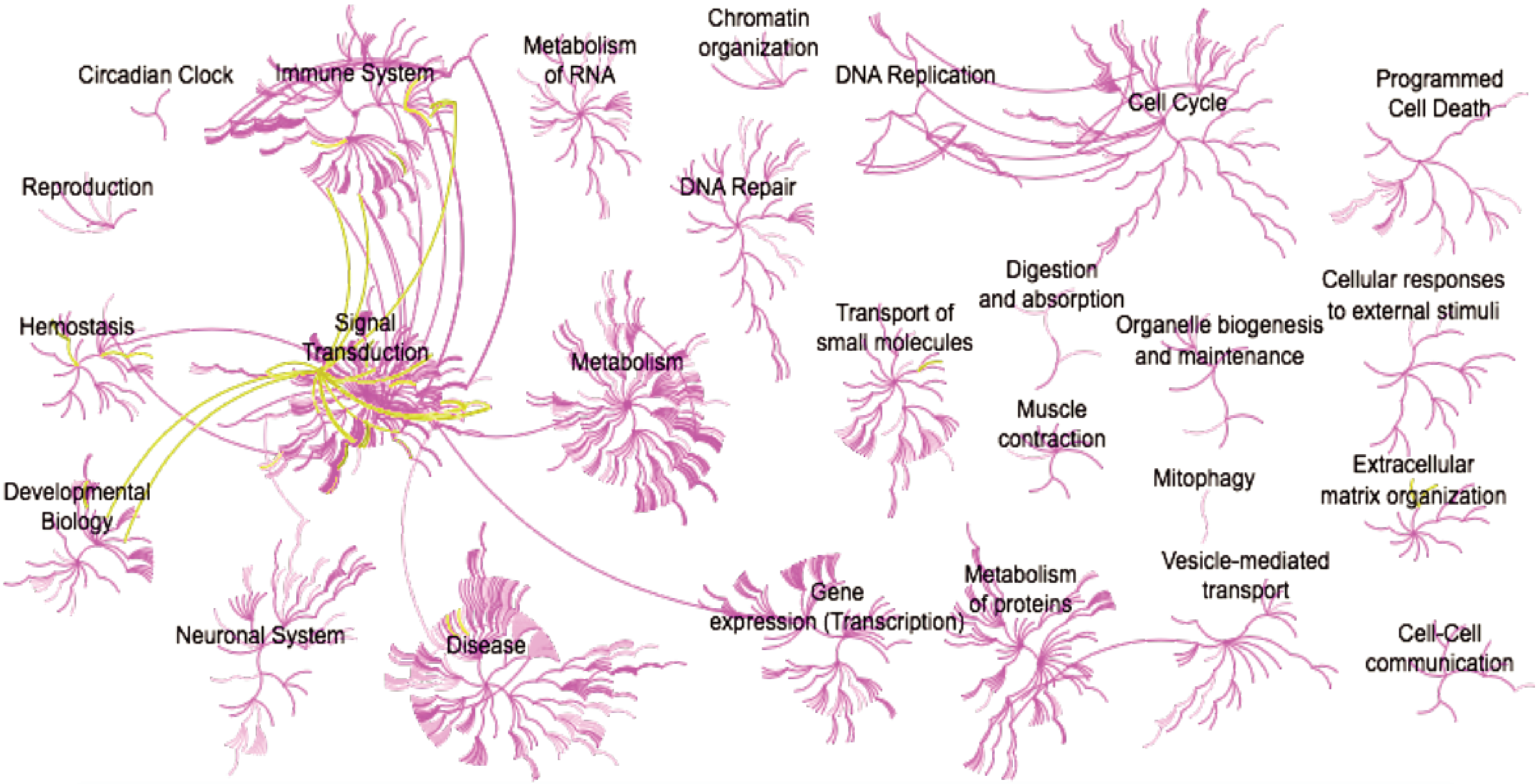

Figure S5. Emergence of major spliceosome pathwyas with higher intron retention possessing transcripts in CLL upon superimposing on NBC

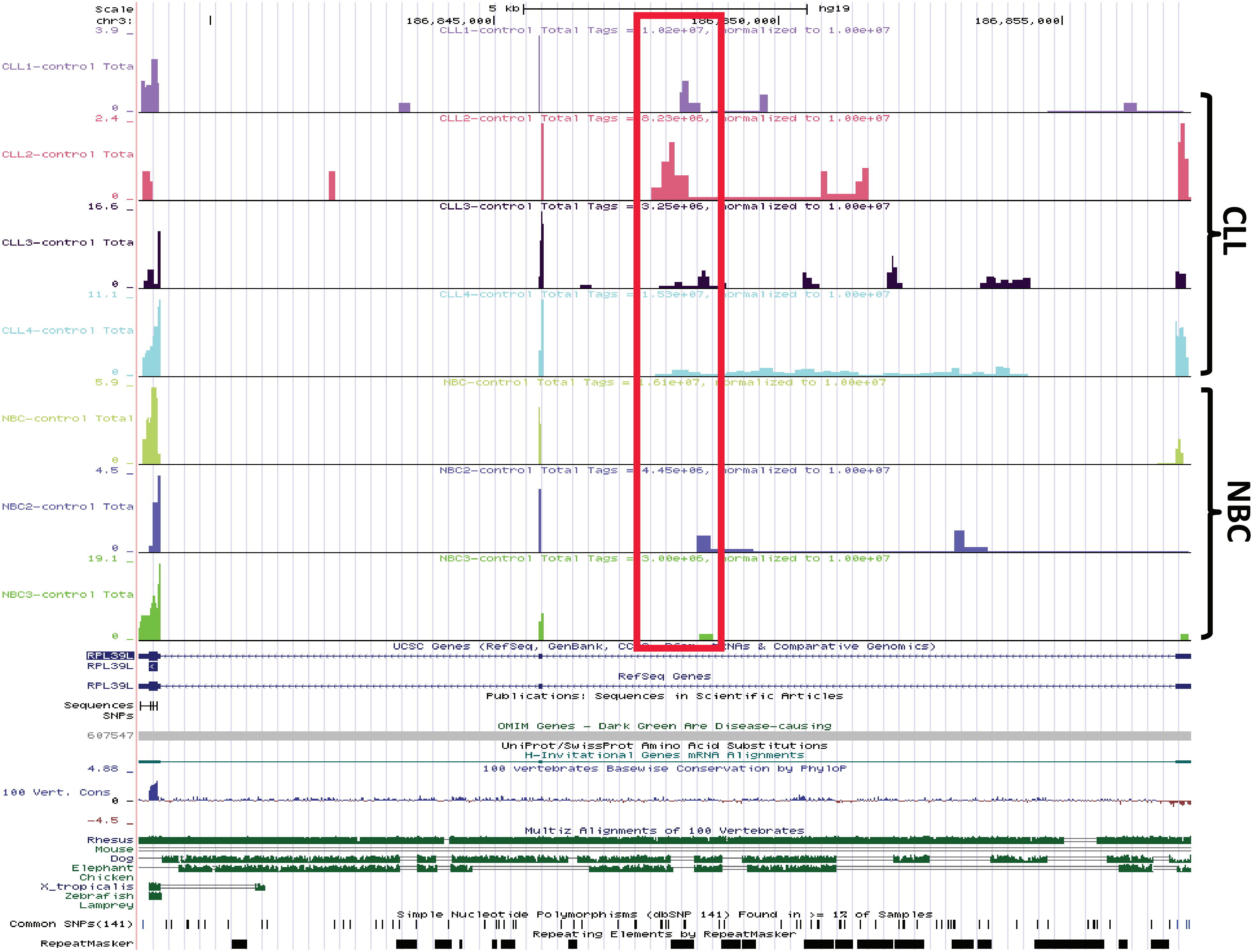

Figure S6A. Tracks of R*PL39L* gene showing intron usage in CLL vs. NBC

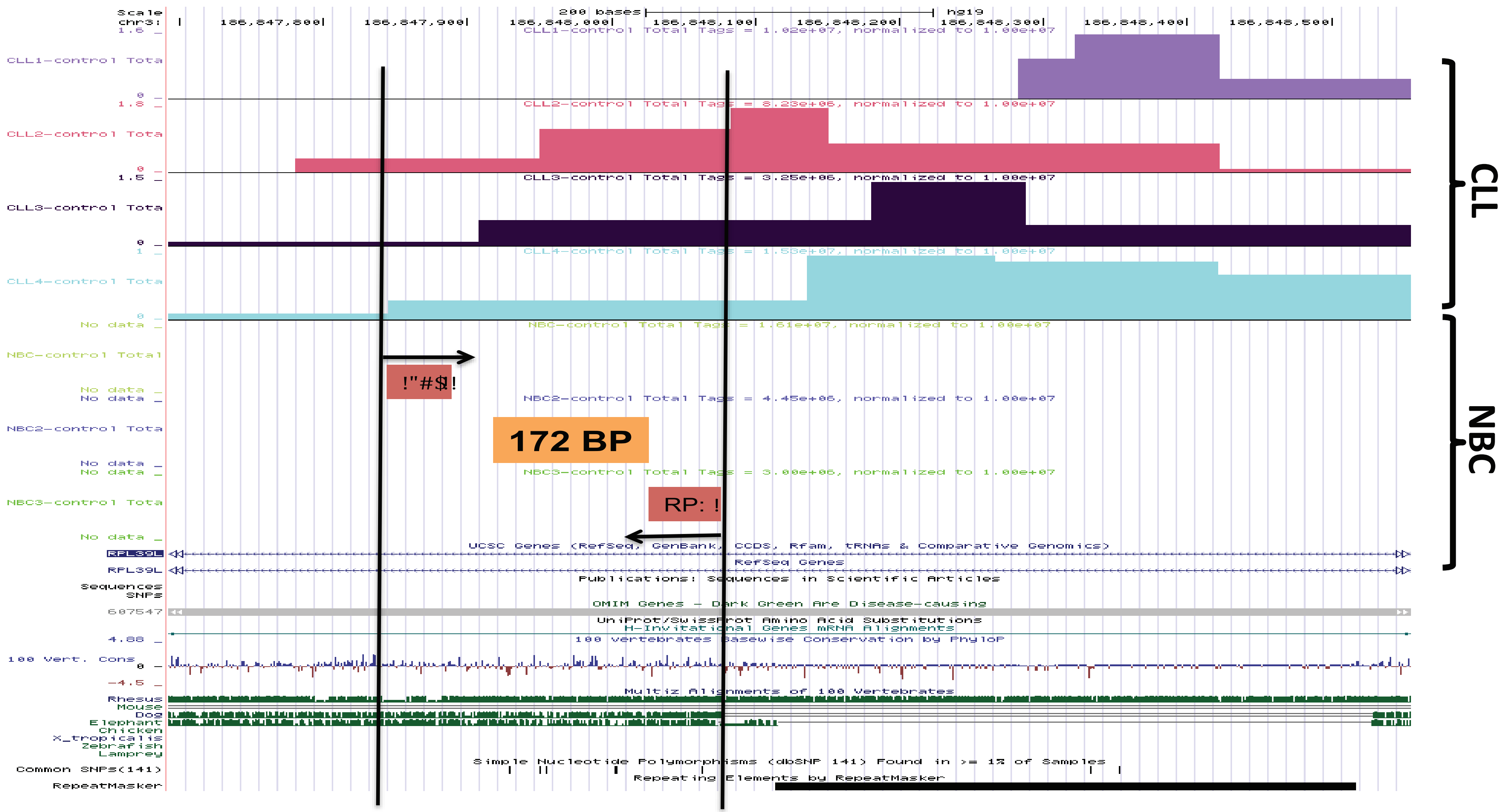

Figure S6B. Tracks of *RPL39L* gene showing intron usage in CLL vs. NBC

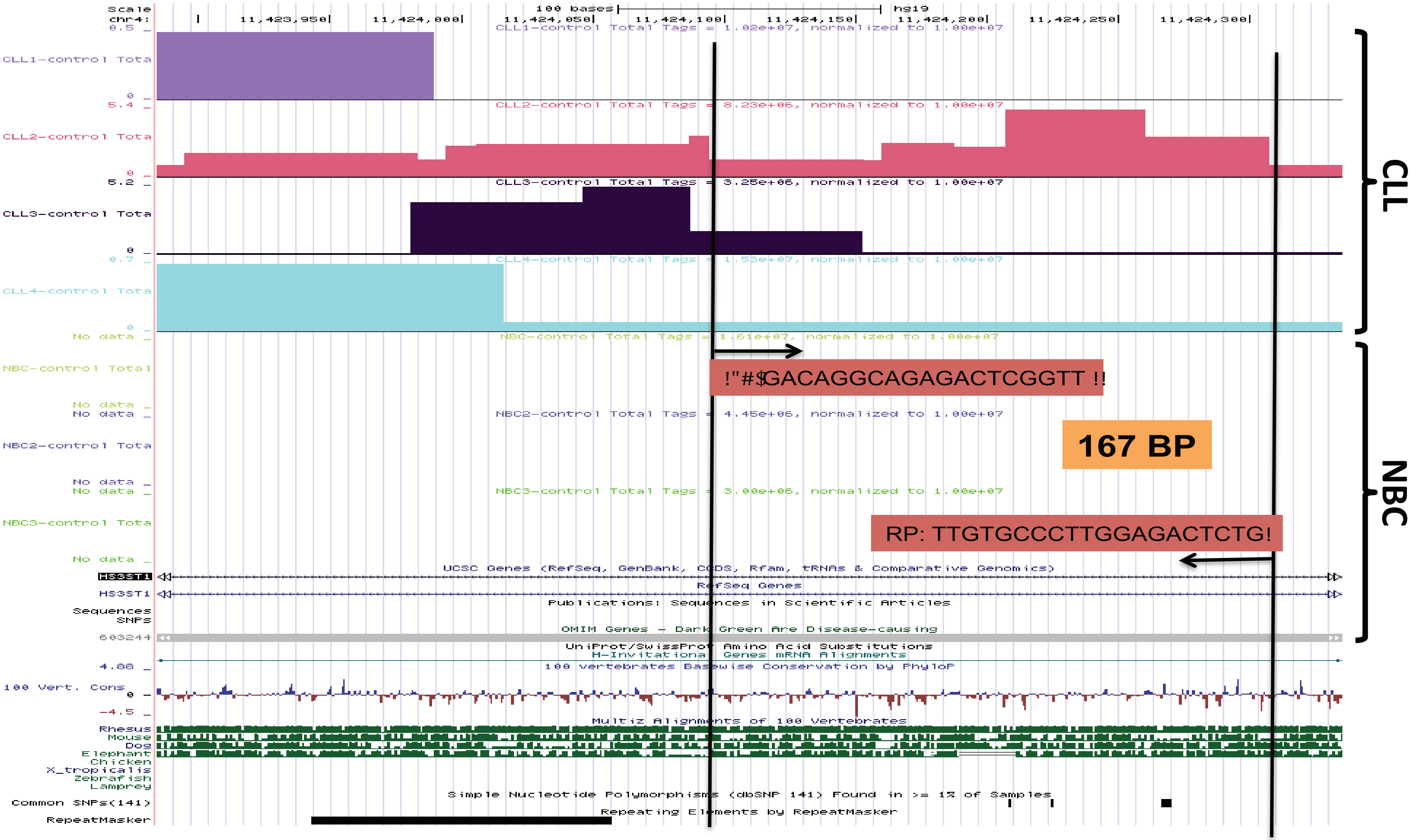

Figure S7A. Tracks of *HS3ST1* gene showing intron usage in CLL vs. NBC

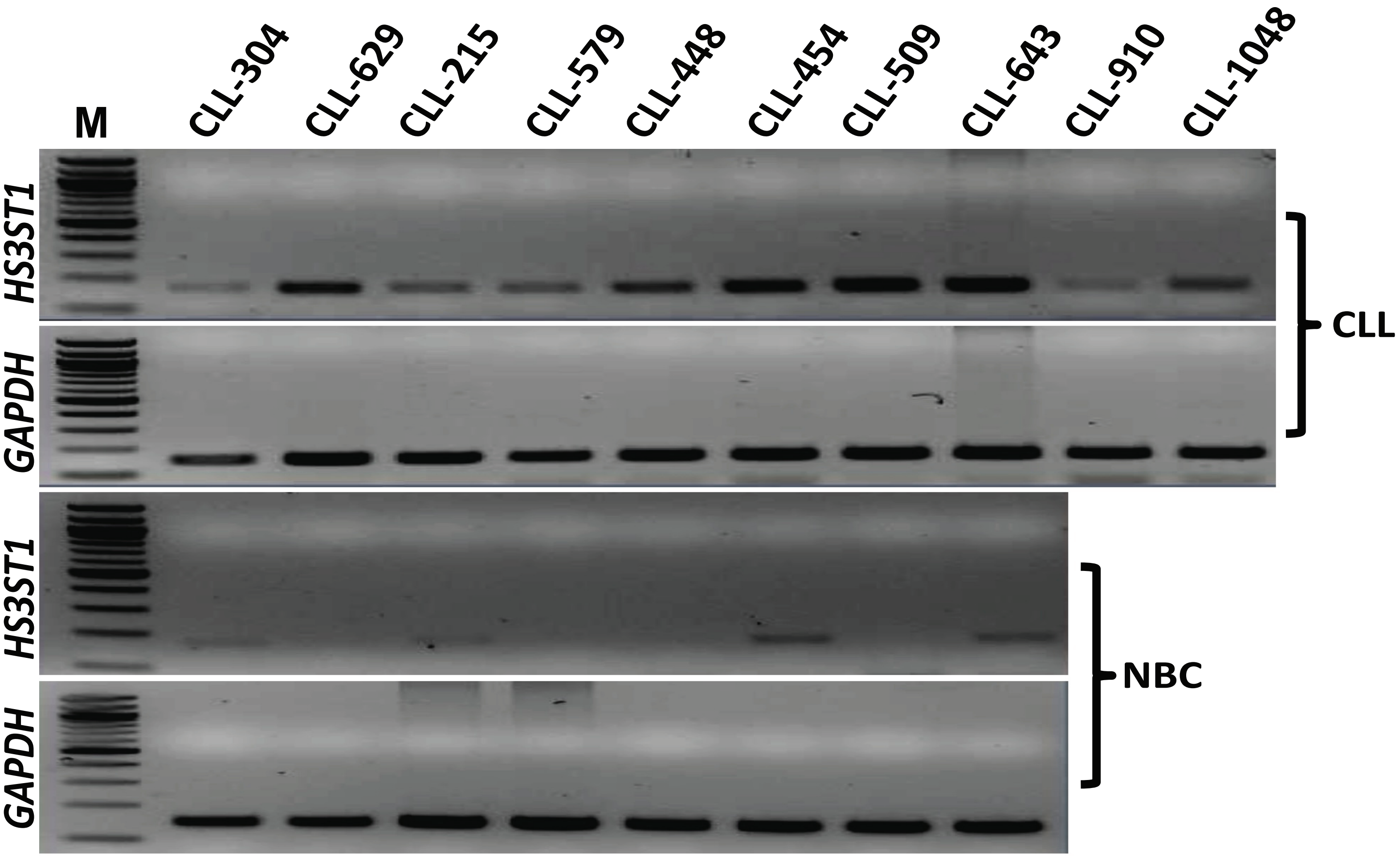

Figure S7B. RT-PCR of *HS3ST1* showing expression between CLL vs. NBC

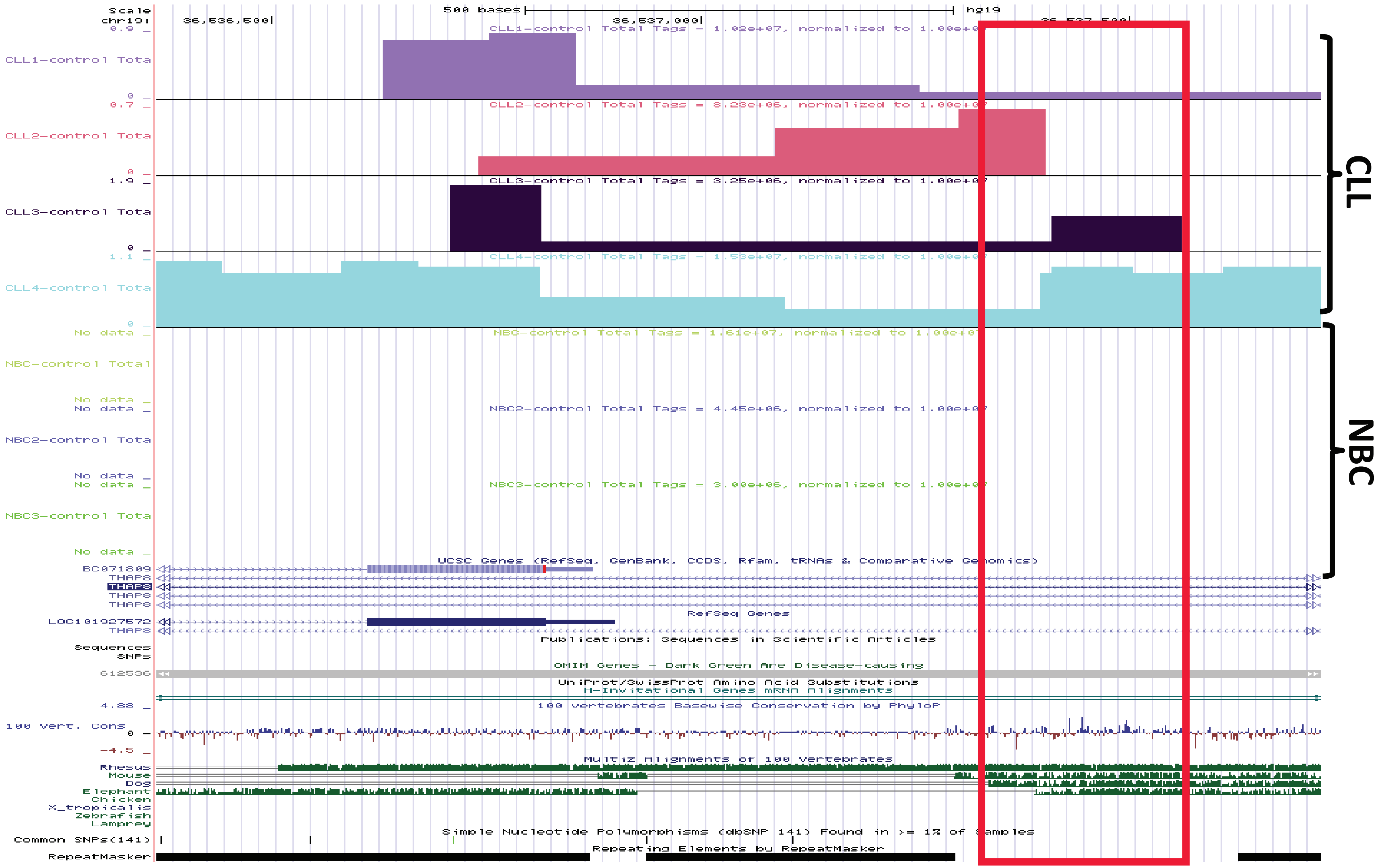

Figure S8A. Tracks of *THAP8* gene showing intron usage in CLL vs. NBC

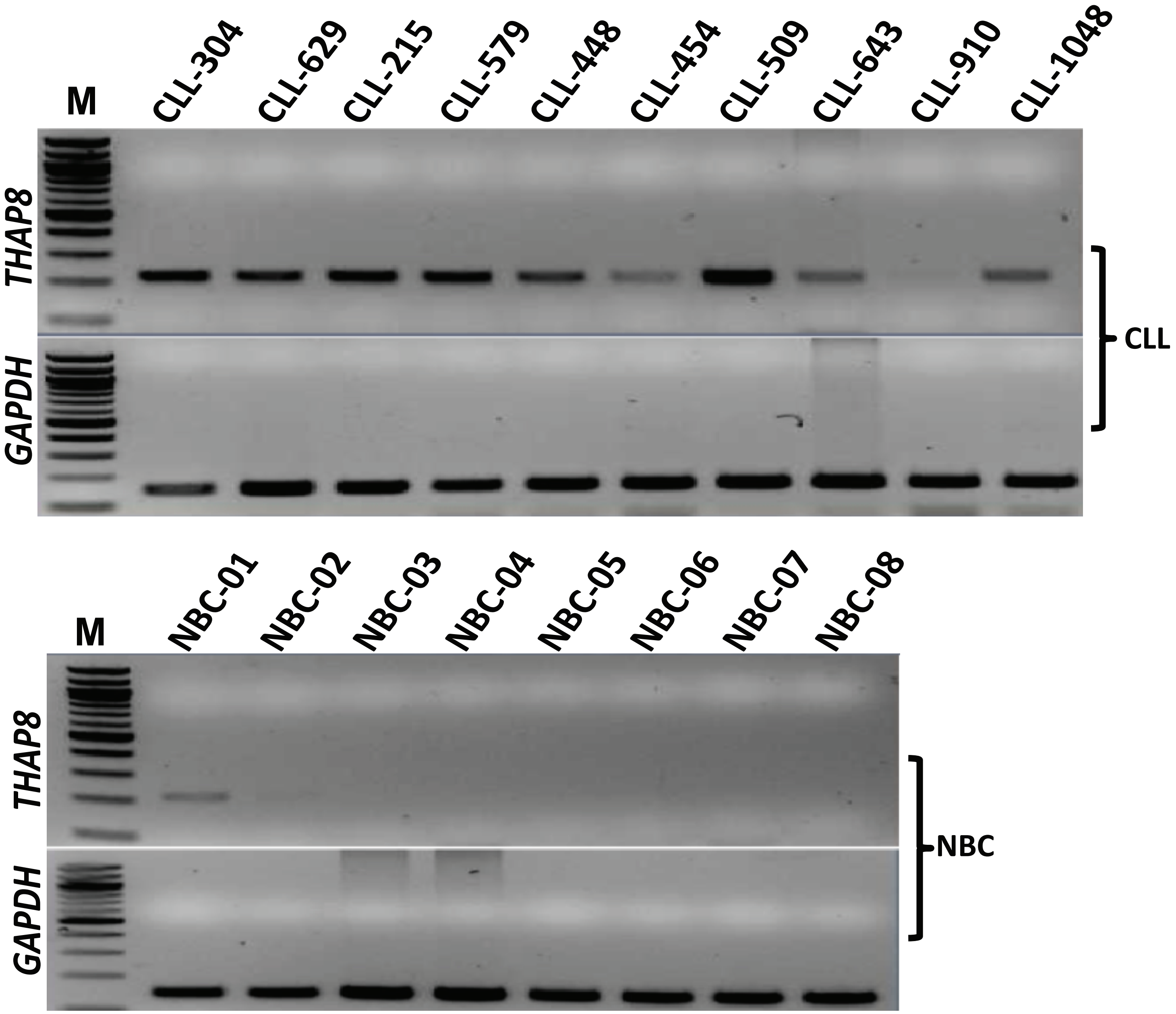

Figure S8B. RT-PCR of *THAP8* showing expression between CLL vs. NBC

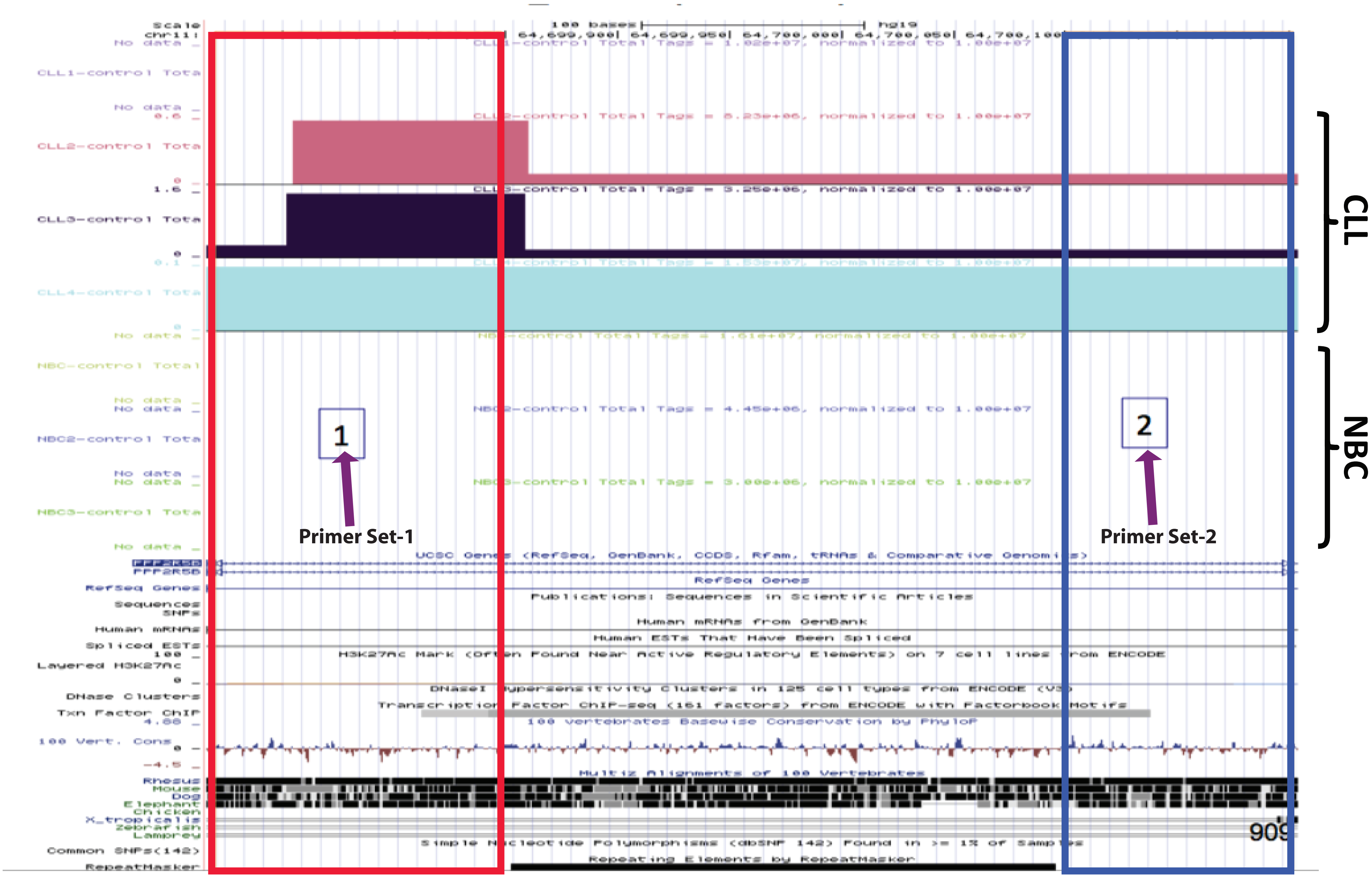

Figure S9A. Tracks of *PPP2R5B* gene showing intron usage in CLL vs. NBC

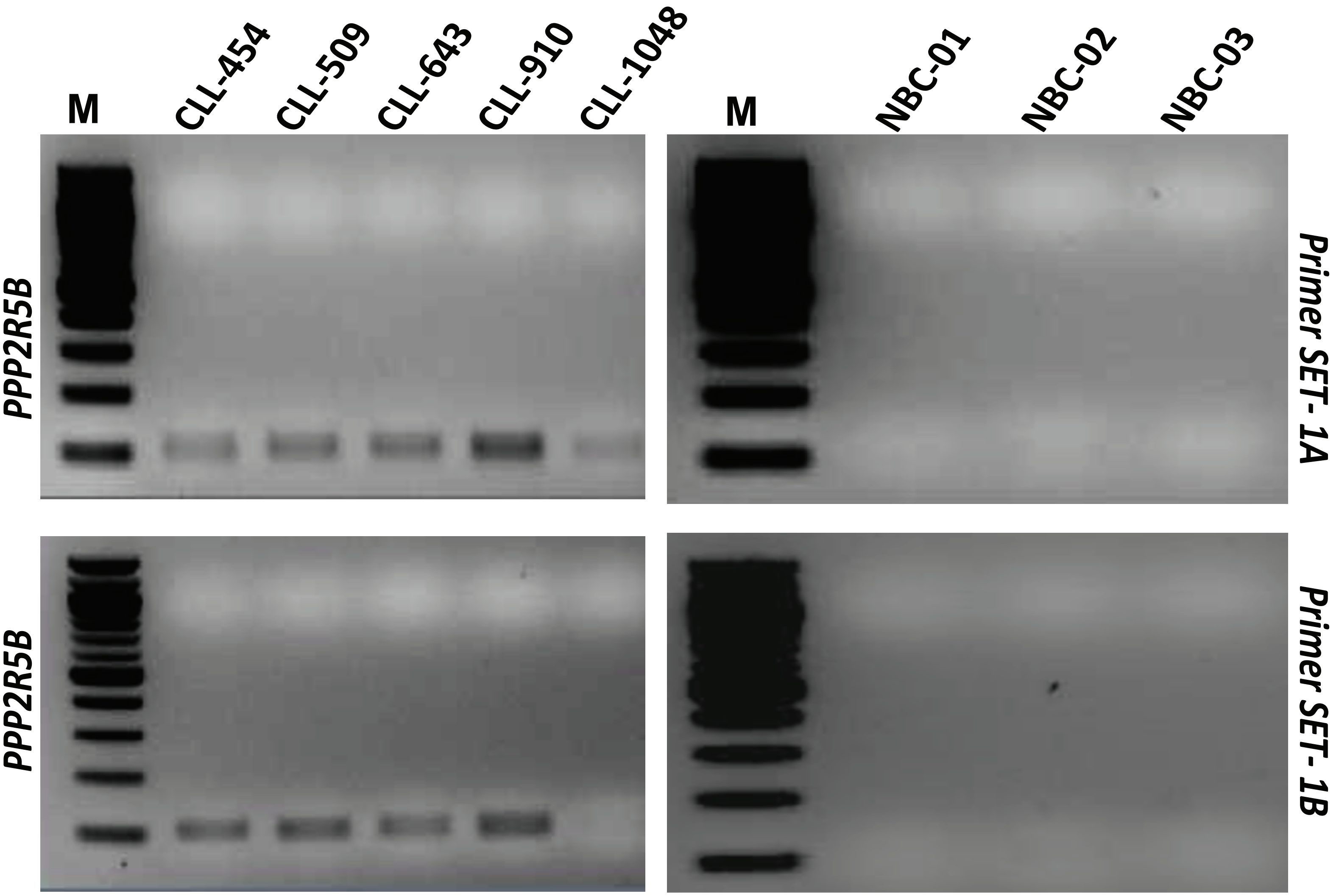

Figure S9B. RT-PCR of *PPP2R5B* showing expression between CLL vs. NBC

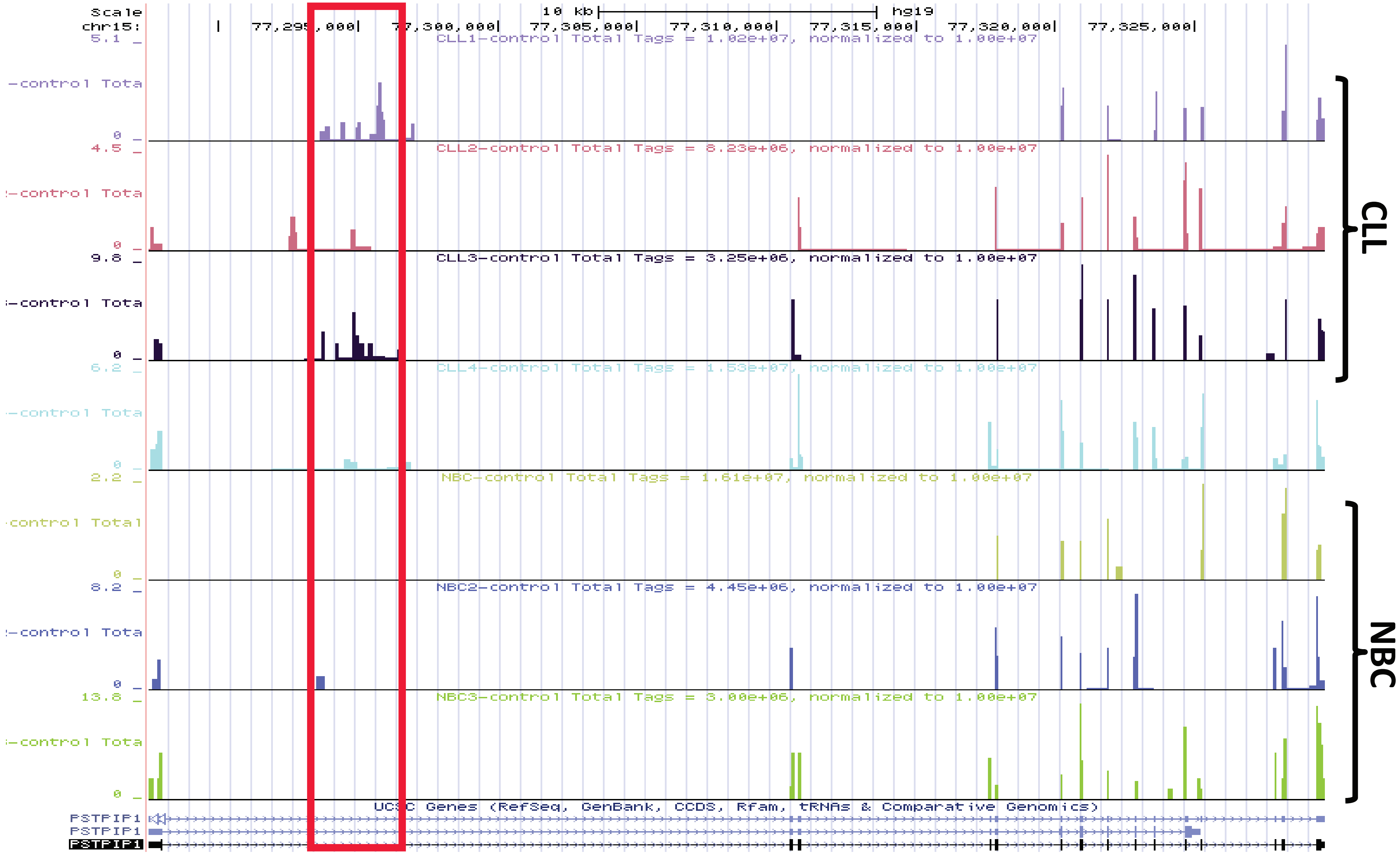

Figure S10A. Tracks of *PSTPIP1* gene showing intron usage in CLL vs. NBC

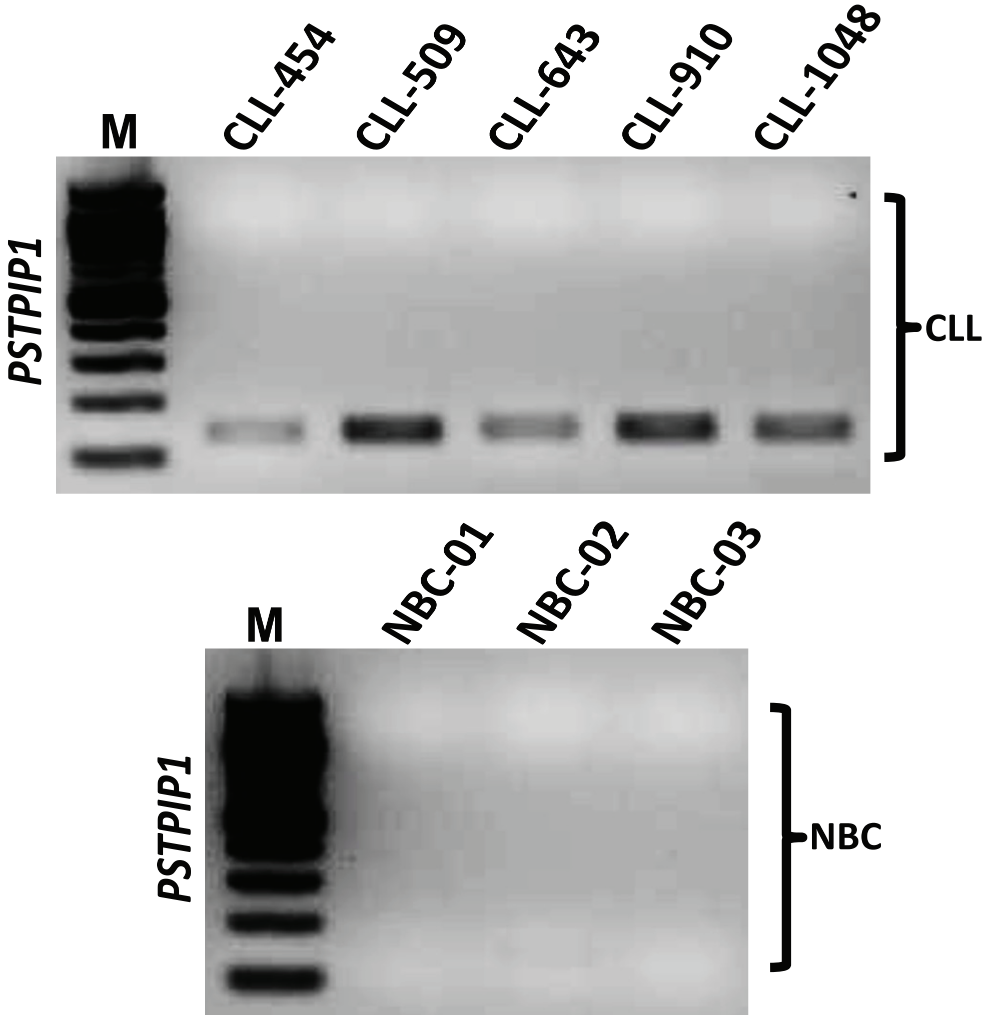

Figure 10B. RT-PCR of *PSTPIP1* showing expression between CLL vs. NBC

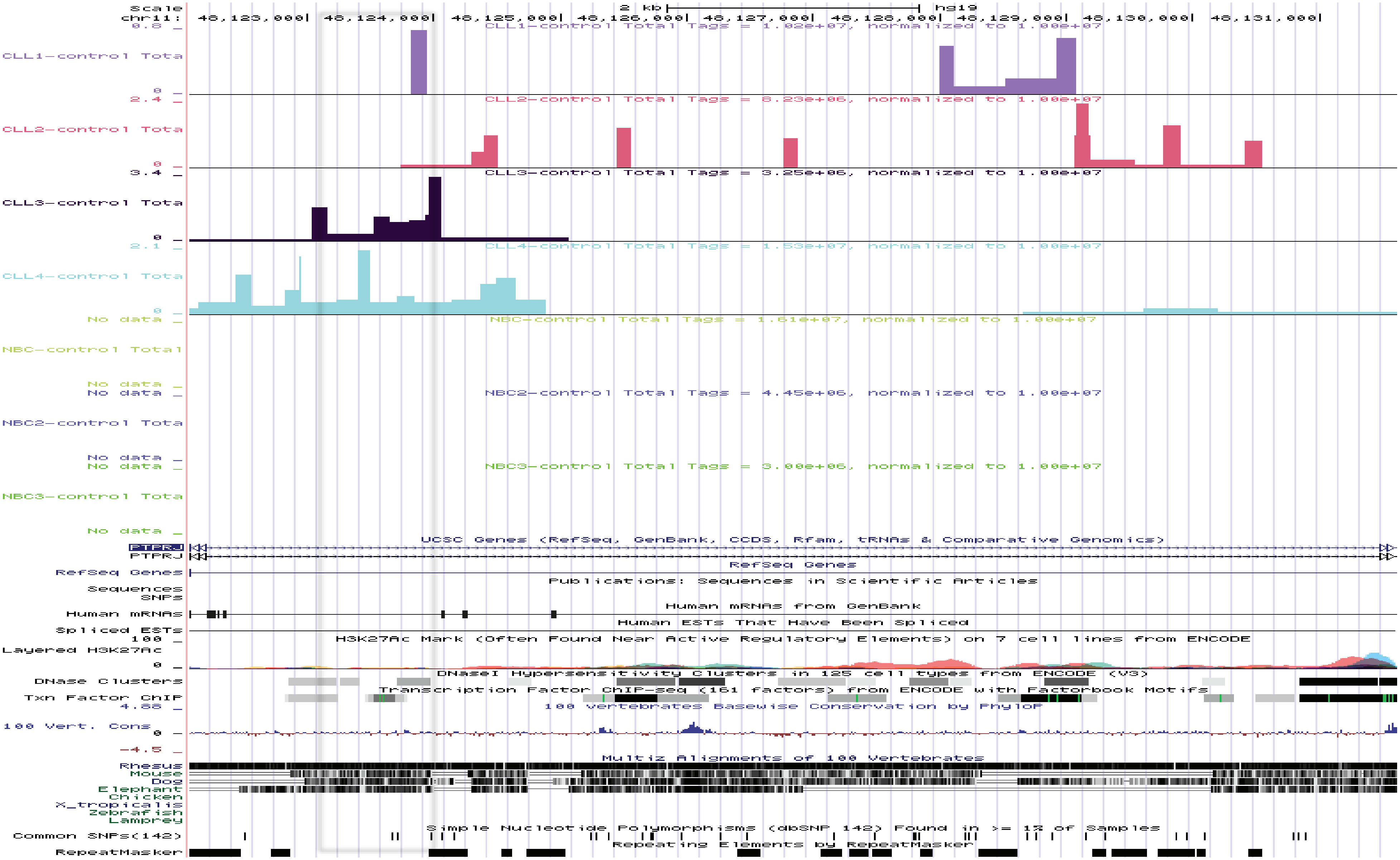

Figure S11A. Tracks of *PTPRJ* gene showing intron usage in CLL vs. NBC

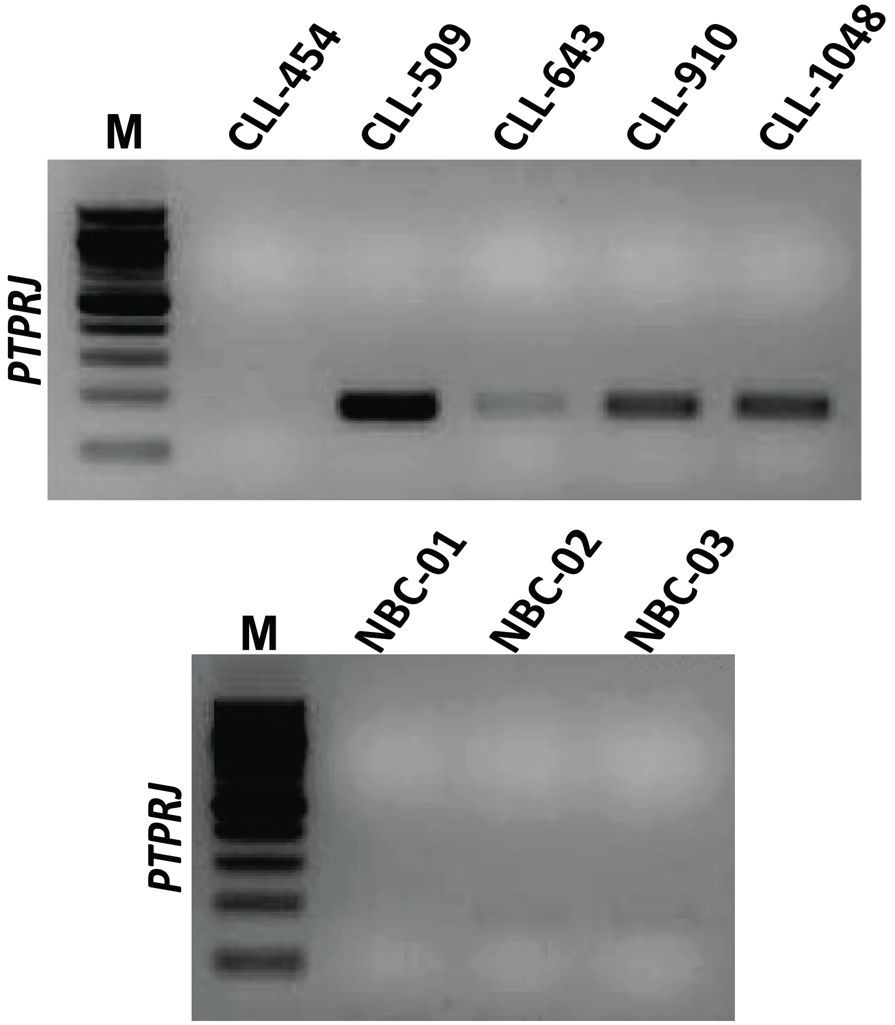

Figure 11B. RT-PCR of *PTPRJ* showing expression between CLL vs. NBC

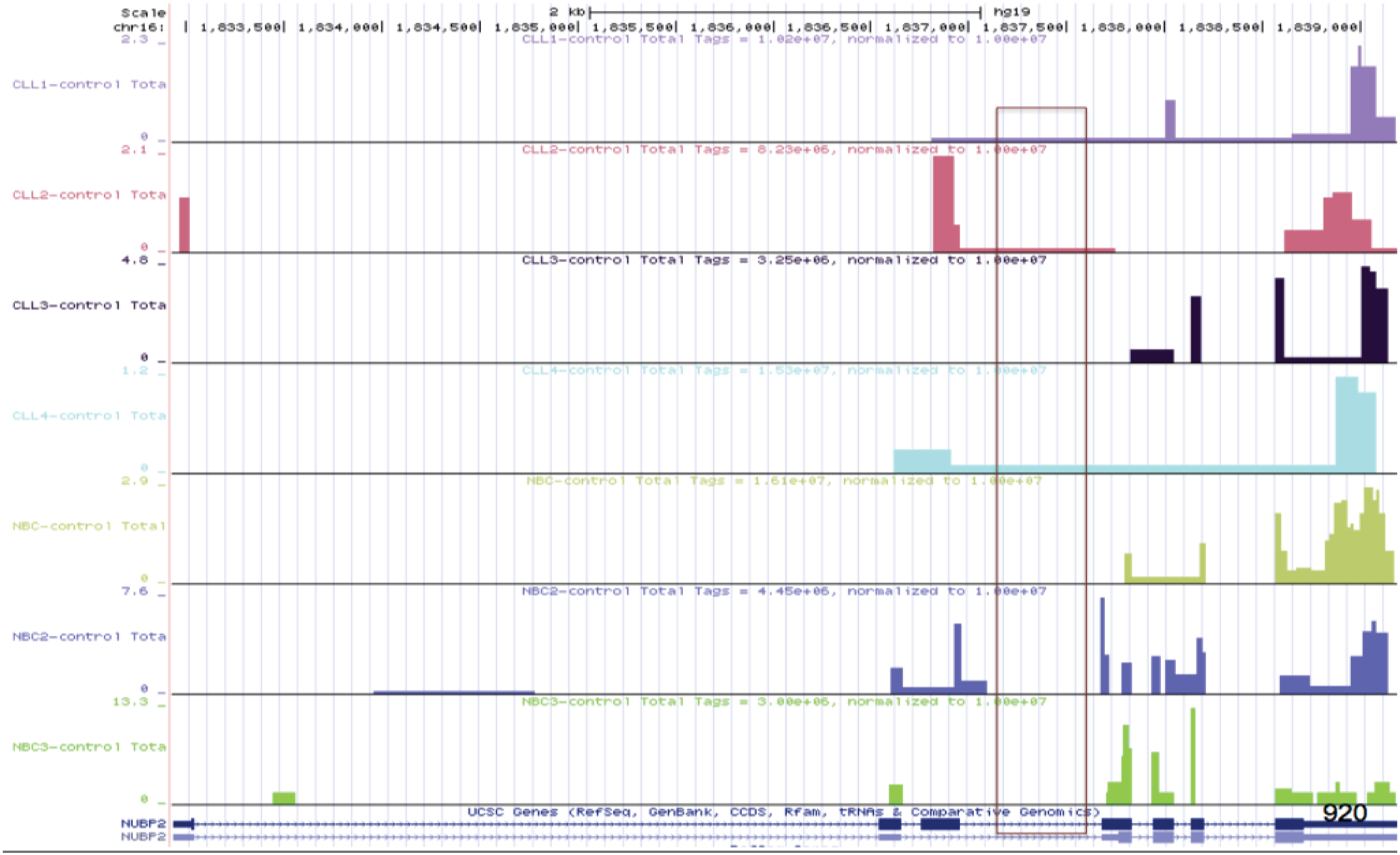

Figure S12A. Tracks of *NUBP2* gene showing intron usage in CLL vs. NBC

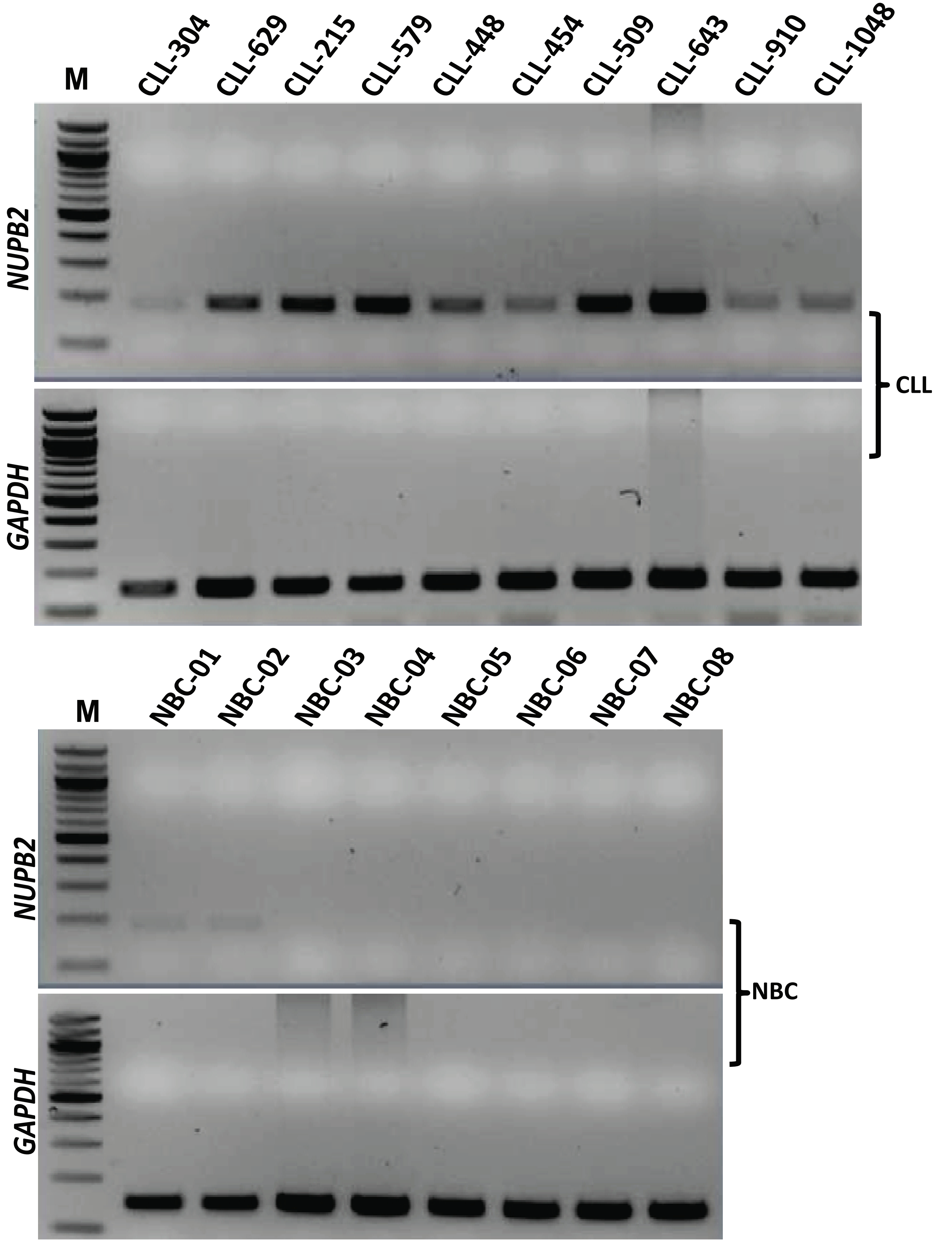

Figure S12B. RT-PCR of *NUPB2* showing expression between CLL vs. NBC

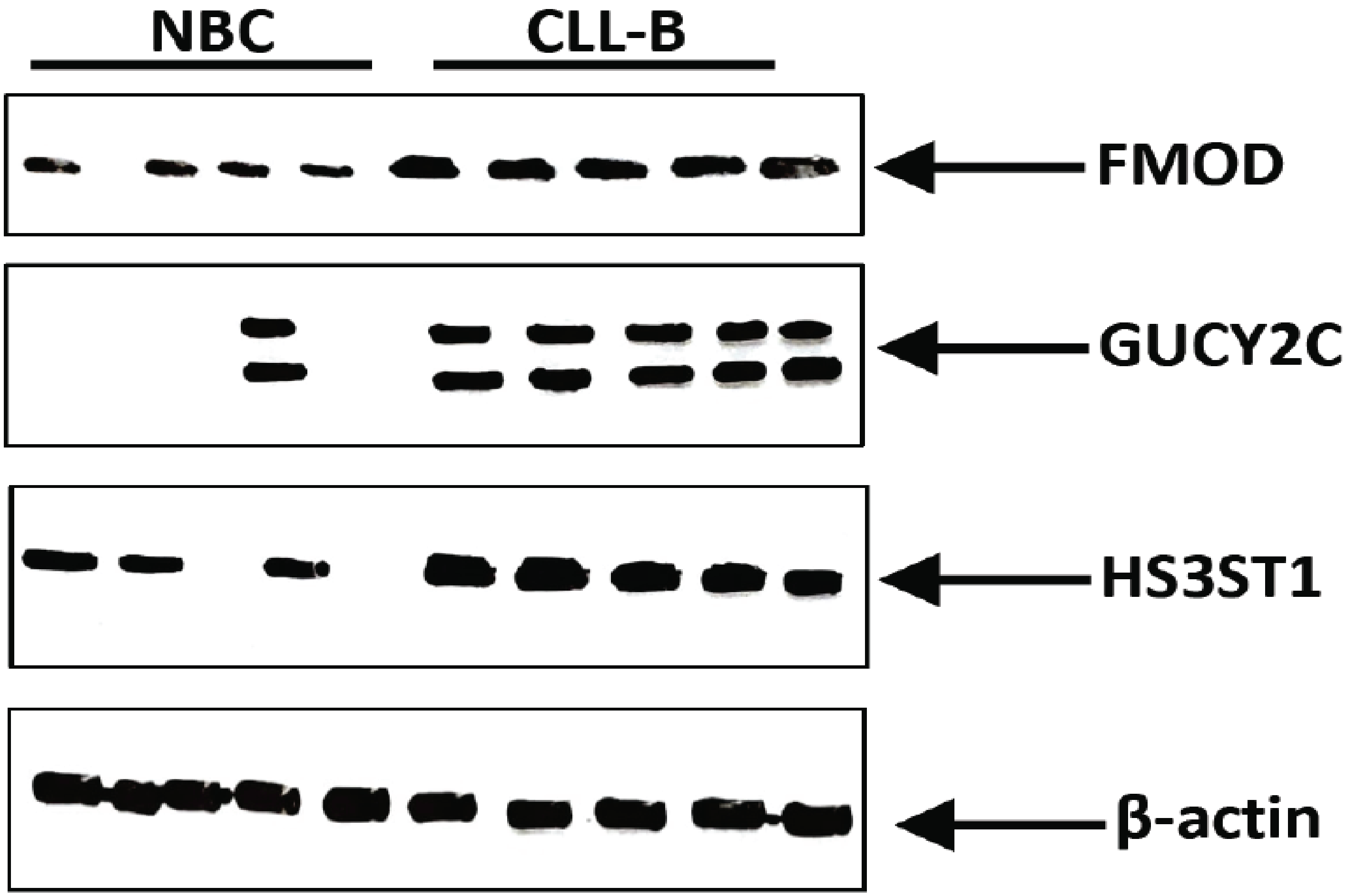

Figure S13: Validation of FMOD, GUCY2C, and HS3ST1 Using Western Blot Technique in NBC and CLL-B Cells

References

1. Vera Alvarez, R, Pongor, LS, Marino-Ramirez, L, and Landsman, D (2019). TPMCalculator: one-step software to quantify mRNA abundance of genomic features. *Bioinformatics* **35**: 1960-1962.

2. Schwaederle, M, Ghia, E, Rassenti, LZ, Obara, M, Dell'Aquila, ML, Fecteau, JF, *et al.* (2013). Subclonal evolution involving SF3B1 mutations in chronic lymphocytic leukemia. *Leukemia* **27**: 1214-1217.
