# Supplementary material for: Aberrant spliceosome activity via elevated intron retention and upregulation and phosphorylation of SF3B1 in chronic lymphocytic leukemia
Source: Mol Ther Nucleic Acids. 2024 Apr 26;35(2):102202. doi: 10.1016/j.omtn.2024.102202 (PMC11154714; doi:10.1016/j.omtn.2024.102202)
Supplement: Document S2. Article plus supplemental information [file mmc3.pdf]

Molecular Therapy
Nucleic Acids
Original Article

Aberrant spliceosome activity via elevated intron retention and upregulation and phosphorylation of SF3B1 in chronic lymphocytic leukemia

Manoj Kumar Kashyap,[1,2,3,12] Hiren Karathia,[8,10,11,12] Deepak Kumar,[1,12] Roberto Vera Alvarez,[6] Jose Vicente Forero-Forero,[5] Eider Moreno,[5] Juliana Velez Lujan,[1] Carlos Ivan Amaya-Chanaga,[1] Newton Medeiros Vidal,[6] Zhe Yu,[1] Emanuela M. Ghia,[1,2,9] Paula A. Lengerke-Diaz,[5] Daniel Achinko,[6] Michael Y. Choi,[1,2,9] Laura Z. Rassenti,[1,2,9] Leonardo Mariño-Ramírez,[6] Stephen M. Mount,[4] Sridhar Hannenhalli,[7] Thomas J. Kipps,[1,2,9] and Januario E. Castro[1,5]

[1]Moores Cancer Center, University of California, San Diego, La Jolla, CA 92093-0820, USA; [2]Division of Hematology Oncology, Department of Medicine, University of California, San Diego, La Jolla, CA 92093, USA; [3]Amity Stem Cell Institute, Amity Medical School, Amity University Haryana, Panchgaon (Manesar), Gurugram (HR) 122413, India; [4]Department of Cell Biology and Molecular Genetics, University of Maryland, College Park, Maryland 20742, USA; [5]Department of Internal Medicine, Division of Hematology-Oncology, Mayo Clinic, Phoenix, AZ 85054, USA; [6]National Center for Biotechnology Information, National Library of Medicine, National Institutes of Health, Bethesda, MD, USA; [7]Cancer Data Science Lab, National Cancer Institute, National Institutes of Health, Bethesda, MD, USA; [8]Advanced Biomedical Computational Science and National Center for Advancing Translational Sciences, National Cancer Institute, National Institutes of Health, Frederick, MD, USA; [9]Center for Novel Therapeutics, University of California, San Diego, La Jolla, CA 92037, USA; [10]Greenwood Genetic Center, Greenwood, SC, USA; [11]Center for Bioinformatics and Computational Biology, University of Maryland, College Park, MD 20742, USA

Splicing factor 3b subunit 1 (SF3B1) is the largest subunit and core component of the spliceosome. Inhibition of SF3B1 was associated with an increase in broad intron retention (IR) on most transcripts, suggesting that IR can be used as a marker of spliceosome inhibition in chronic lymphocytic leukemia (CLL) cells. Furthermore, we separately analyzed exonic and intronic mapped reads on annotated RNA-sequencing transcripts obtained from B cells (*n* = 98 CLL patients) and healthy volunteers (*n* = 9). We measured intron/exon ratio to use that as a surrogate for alternative RNA splicing (ARS) and found that 66% of CLL-B cell transcripts had significant IR elevation compared with normal B cells (NBCs) and that correlated with mRNA downregulation and low expression levels. Transcripts with the highest IR levels belonged to biological pathways associated with gene expression and RNA splicing. A >2-fold increase of active pSF3B1 was observed in CLL-B cells compared with NBCs. Additionally, when the CLL-B cells were treated with macrolides (pladienolide-B), a significant decrease in pSF3B1, but not total SF3B1 protein, was observed. These findings suggest that IR/ARS is increased in CLL, which is associated with SF3B1 phosphorylation and susceptibility to SF3B1 inhibitors. These data provide additional support to the relevance of ARS in carcinogenesis and evidence of pSF3B1 participation in this process.

INTRODUCTION

Chronic lymphocytic leukemia (CLL) is the most common B cell malignancy among adults, characterized by apoptosis defects that provide a survival advantage to neoplastic B-lymphocytes. High-risk patients such as those with a deletion of chromosome 17 (del17p) or TP53/ATM mutation generally fail to respond to conventional chemotherapy. Thus, understanding the biology of CLL cells may enlighten the path to novel therapeutic development.[1,2]

Alternative RNA Splicing (ARS) is a widespread process in eukaryotes that contributes to post-transcriptional mRNA isoform diversity. These isoforms are essential for several physiological functions such as cell division, differentiation, stress response, and lineage specification.[3–8] Many RNA-sequencing (RNA-seq)-based approaches are used to characterize the basic pattern of ARS events such as exon skipping, alternative 5′ or 3′ splice sites, mutually exclusive exons, and intron retention (IR).[9,10] Generally, these characterizations are based on the events captured based on the abundance of RNA-seq reads that map to the annotated composition of the exons and introns of a transcript. An examination of mapped RNA-seq reads helps to infer the gene expression levels, splicing patterns, and regulatory mechanisms operating within the transcriptome and also helps to unravel the complexity of gene expression and regulation in biological systems.[11] The exonic reads reflect mature cytoplasmic mRNAs, and recent studies suggest that mapping the RNA-seq reads to the defined intronic regions of a gene essentially reflects nascent transcripts (pre-RNA).[10,12,13]

Received 14 July 2022; accepted 24 April 2024;
https://doi.org/10.1016/j.omtn.2024.102202.

[12]These authors contributed equally

Correspondence: Manoj K Kashyap, Amity Stem Cell Institute, Amity Medical School, Amity University Haryana, Manesar, India.
E-mail: mkkashyap@ggn.amity.edu
Correspondence: Januario E. Castro, Mayo Clinic, Phoenix, AZ, USA.
E-mail: castro.januario@mayo.edu

This is an open access article under the CC BY license (http://creativecommons.org/licenses/by/4.0/).

EISA (exon-intron split analysis) is a practical tool that analyses separately the effect of pre-mRNA and mRNA forms a transcript. EISA quantifies changes in the exonic and intronic reads in different conditions and effectively characterizes post-transcriptional changes in gene expression.[13] In recent years, IR has been the focus of several studies for its functional importance in cancer studies, its association with alternative RNA splicing, and carcinogenesis.[14–17] Our previous studies support this concept, as we demonstrated *in vivo* and *in vitro* evidence that IR is significantly elevated in CLL cells compared with normal B cells (NBCs). However, it is unknown how the regulatory pathways contribute to the increase in the IR/ARS in CLL and what fraction of the intron-using transcript (a proxy of pre-mRNA expression) contributes to the total RNA expression activity in CLL and other malignancies.[18]

The spliceosome machinery is a protein complex that regulates RNA splicing in eukaryotic cells. It comprises many splicing factors, and the best characterized include splicing factor 3B subunit 1 (SF3B1), U2 Small Nuclear RNA Auxiliary Factor 1(U2AF1), and serine and arginine rich splicing factor 2 (SRSF2). Evidence suggests that mutations of the splicing factor genes are associated with the aberrant splicing process during tumor development and metastases.[19,20] In CLL, mutations of *SF3B1* are found in ∼10%–15% of cases and constitute an independent prognostic factor associated with rapid progression, short survival, and lack of response to conventional treatments; hence, SF3B1 appears to be a highly relevant target for CLL therapy.[21,22] Nevertheless, how the mutated SF3B1 (Mut-SF3B1) or imbalanced expression of the SF3B1 in CLL cells affects the accumulation of IR on transcripts is mostly unknown. Our previous *in vitro* study suggests that hyper-activation of *SF3B1* might be related to transcripts with elevated IR in CLL cells. It might explain phenotypes associated with survival, cell proliferation, and possibly treatment resistance.[18]

PLAD-B and FD-895 are two polyketides known to modulate the ARS by targeting SF3B1. This protein's specific inhibition is postulated to be responsible for their mechanism(s) of antitumor activity.[18,23–25] Our previous work demonstrated that these polyketides modulate IR level on a few selected genes in the treated CLL cells, but not in untreated CLL or NBC samples.[18] Interestingly, we also found no significant difference in expression of Mut-SF3B1 vs. wild-type *SF3B1* (WT-*SF3B1*) in CLL cells, speculating that the contributions of *SF3B1* to changes of the IR may not be mediated through its transcriptional activity, but rather its activity at the protein level.[18] There are studies where downregulation of intron retention events was correlated with mutational status of SF3B1.[26] However, it is not yet clear how the activity of SF3B1 contributes to the regulation of IR/ARS in CLL cells. We carried out this study to achieve the following specific aims: (1) Obtain a profile of the sets of transcripts associated with IR/ARS and their correlation with prognostic factors in CLL. For this, we use bioinformatics tools to compare in-depth RNA-seq data (available from the European Genome-phenotype Archive [EGA]) between CLL and NBC.[2,27,28] (2) We further validated the changes in abundance of transcripts previously found altered, using RNA isolation followed by RT-PCR of another cohort of CLL vs. NBCs. (3) Attempt to elucidate the mechanistic pathway associated with IR/ARS alterations in CLL patients and the relevance of the function of SF3B1 at the protein level (Figure 1).

RESULTS

Our initial study found that the intron/exon's ratio was significantly higher in CLL than in NBCs (Figure S1). The observations were quite intriguing, so we decided to test our observation in a large cohort of CLL and NBC samples. For the same, we analyzed the RNA-seq data obtained from EGA.[27]

Classification of RNA-seq data

The immunoglobulin heavy chain variable region genes (IGHV) were available for 97 (99%) CLL samples. A total of 41 patients had CLL cells expressing unmutated IGHV (HR) with 98% homology to the germline IGHV, whereas 56 had CLL cells expressing mutated IGHV genes (LR). One sample without known IGHV mutational status was excluded from the analysis (Table S1). Eighty-five patients had CLL cells harboring WT-*SF3B1*, whereas nine patients had CLL cells carrying the Mut-*SF3B1* gene. The *SF3B1* mutational status was not available for four samples, so these samples were excluded from the analysis (Table S1).

Identification of IR events in CLL vs. NBC samples

The transcriptome-wide data were analyzed using transcript per million (TPM) analysis. The transcripts were expressed in at least 60% of both NBCs and CLL samples and were subsequently analyzed for IR. The global distribution of IR for 14,811 transcripts is shown in the volcano plot of Figure 2. The lists of transcripts are provided in Table S2. Also, an overall pattern for intron/exon ratio for all CLL cells (regardless of their subtypes) vs. NBC is shown in Figure S2A. The scatterplot of CLL cells vs. NBCs presents overall distribution of IR (Figure S2A) where we observed that there were 20,906 transcripts (61%) above the regression line and 13,894 (39%) below the regression line, indicating that a large majority of transcripts had >1 intron/exon ratio in CLL cells as compared with NBC, consistent with our previously published study on CLL.[18]

The volcano plot created to schematize the differential IR between two cell types gives a global view of expression level and IR. The upper right quadrant in Figure S2B represents values positive for both quantities.

The isoforms plotted in this quadrant were interpreted as having more expression and more IR in CLL cells. The lower right quadrant was for positive values of the "log2 of CLL cells (intron/exon)/NBC (intron/exon)" and low values of the "log10 (CLL cells [transcript expression]/NBC [transcript expression])". This quadrant showed isoforms with more IR in CLL cells but with lower expression. The upper left quadrant shows negative values of "$\log_2$ of CLL cells (intron/exon)/NBC (intron/exon)" and positive values of the "$\log_{10}$ (CLL cells [transcript expression]/NBC [transcript expression])". It showed more IR in NBCs but more expression in CLL cells. Finally,

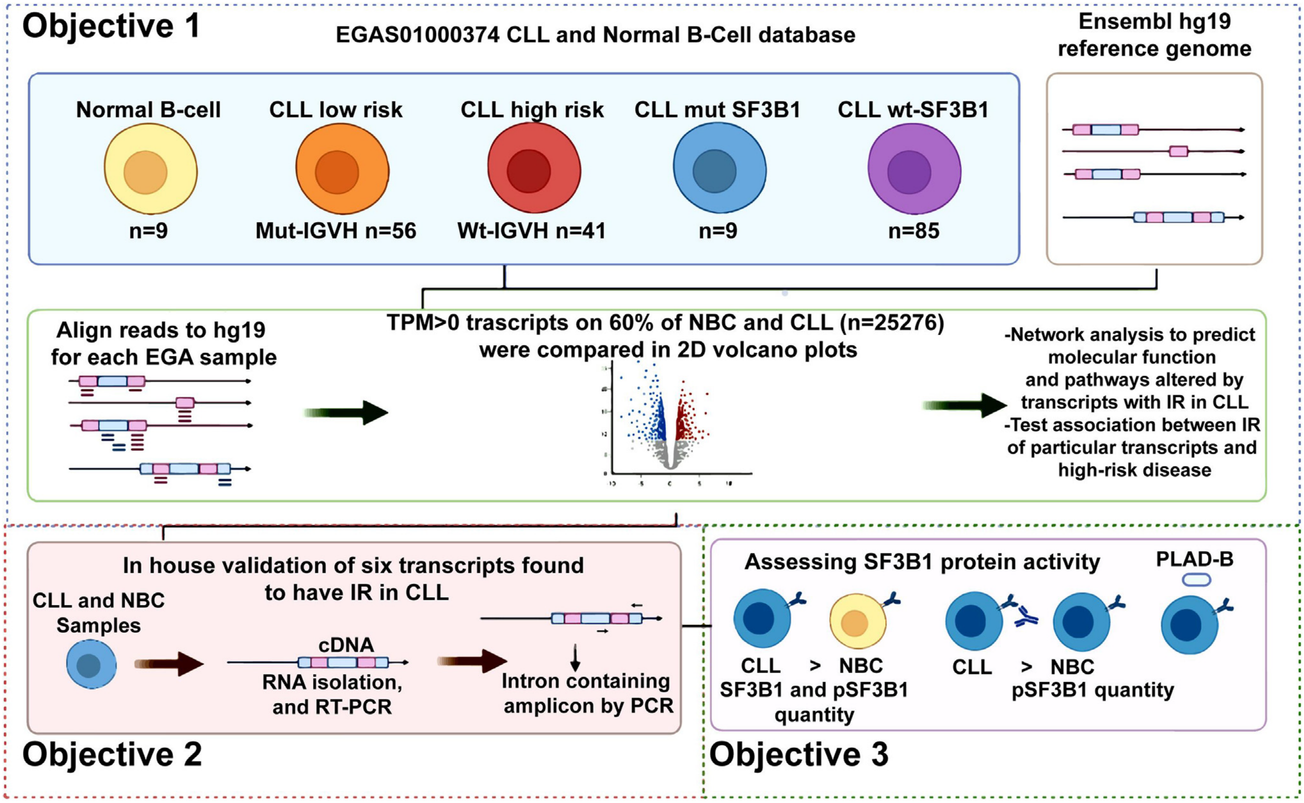

Figure 1. The workflow for RNA-seq analysis for IR study in CLL vs. NBC

For studying the global IR pattern between CLL vs. NBC, the RNA-seq data obtained from EGA was processed for analysis after alignment with the reference genome hg19. The transcripts with >0 TPM values were selected if those were detected in at least 60% of the population. A comparative analysis of IR between different CLL cell subtypes vs. NBCs was done. Further biological pathways and molecular function were studies using PANTHER. An SF3B1 centric subnetwork was constructed to study if there are involvements of genes/transcripts in RNA splicing. A selected number of transcripts to show IR were validated using cDNA derived from CLL cells and NBC (synthesized from total RNA treated with DNaseI to remove the DNA contamination) after subjecting to RT-PCR followed by agarose gel electrophoresis. SF3B1 and pSF3B1 profiling was done after IgM stimulation of CLL and NBCs. Further, the effect of macrolide PLAD-B and fludarabine (F-ara-A) was studied *in vitro* on CLL cells after treatment for different time points. The protein was isolated after cell lysis and after SDS-PAGE; the protein was transferred on the membrane for detection of SF3B1, pSF3B1, and loading control (β-actin).

the lower left quadrant was the opposite of the right upper quadrant. It shows more expression and more IR in NBCs than in CLL cells (Figure S2B). The volcano plots making a comparison between CLL cells harboring Mut-SF3B1 and NBC (Figure 2B), CLL cells harboring Wt-*SF3B1* and NBC (Figure 2C), CLL cells harboring Mut-*SF3B1* and CLL cells harboring Wt-*SF3B1* (Figure 2D) and finally M-CLL cells and U-CLL cells (Figure 2E) have been interpreted similarly.

Further, the scatterplot of Figure 2F shows the comparison between IR ratio on the x axis (Log_2 CLL intron/NBC intron) and exon ratio on the y axis (Log_2 CLL exon/NBC exon), for all transcripts between CLL cells vs. NBC.

Association between IR and transcript expression in CLL cells

To assess the transcripts' global association of IR and expression of the transcripts, we first took the complete 25,276 reference transcripts. We classified them based on levels of IR and expression of the transcripts in CLL cells and NBCs using a one-sided Wilcoxon test (see materials and methods). Using this, we found six sets of distinct transcripts: set-IA, set-IB, and set-IC, corresponding to transcripts contributing to high IR with upregulation, high IR with downregulation, and high IR with non-differential expression in CLL. Similarly, set-IIA, set-IIB, and set-IIC correspond to transcripts contributing similarly to three sets in NBC. We found a total of 16,725 transcripts contributing to differentially intron-retaining transcripts in CLL cells and NBCs, among those, 11,969 (set-I = 71%) were significantly high intron-retained in CLL cells, and 4,756 (set-II = 29%) were significantly high used in NBC with p value <0.05 (FDR = 5%). Within the 11,969 transcripts, the set-IA contains 10,436 (87%), set-IB contains 188 (2%), and set-IC contains 1,345 (11%). We constructed a 2×3 contingency table and performed a chi-square test. The chi-square test value was highly significant with a p-value <1.0e−300. The data presented in the bar graph (Figure 3A), show overexpression of high and low intron-retaining transcripts in CLL cells and NBCs. These results suggest that in CLL cells the IR contributes positively to the upregulation of corresponding transcripts.

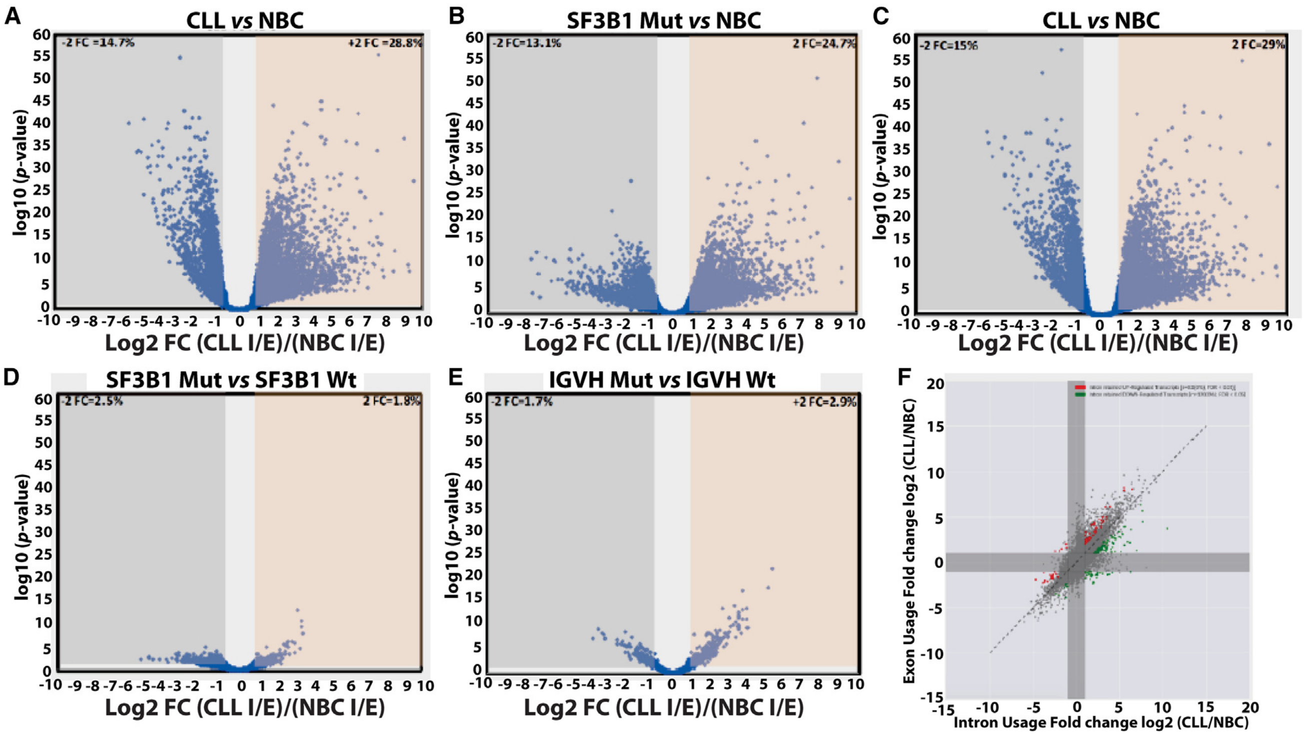

Figure 2. RNA-sequencing analysis for global intron retention in CLL vs. normal B cells and correlation with transcript expression

RNA-seq analysis was conducted on sequences obtained from the EGA data derived from CLL samples (n = 97 including high risk = 41, low risk = 56) and normal B cells (n = 9 from healthy controls in triplicate). (A–E) Volcano plot displaying the differential intron retention log2 ratios TPM (intron/exon) in different combinations including (A) CLL-B cells and NBCs (CLL-B vs. NBC). The scatterplot shows 14811 transcripts for intron retention between CLL-B cells vs. NBCs. (B) CLL-B cells carrying Mut-SF3B1 and NBC (Mut-SF3B1 vs. NBC). (C) CLL-B cells carrying Wt-SF3B1 and NBC (Wt-SF3B1 vs. NBC). (D) CLL-B cells carrying Mut-SF3B1 and CLL-B cells carrying Wt-SF3B1 (Mut-SF3B1 vs. Wt-SF3B1). (E) CLL-B cells carrying Mut-IgV$_H$ and CLL-B cells carrying Wt-IgV$_H$ (Mut-IgV$_H$ vs. Wt-IgV$_H$). The bottom black line represents the diagonal regression line where intron retention ratios are equal in both samples compared. (F) Further, the data were presented in form of a quadrant between CLL-B cells vs. NBCs to show the distribution of ratio of intronic and exonic TPM values for 14,811 transcripts.

To study the sample-wise association between the IR and transcript expression, we sorted the set-I (11969 transcripts), ranked them by p value from lowest to the highest order (Figure 3A), and selected the top 200 transcripts (see materials and methods and supplemental information). We recorded values for these 200 transcripts; for IR and transcript expression observed in all CLL cells and NBC samples. We segregated the CLL samples based on IGHV mutational status in order to understand the association with disease prognosis. An overall sample-wise IR in CLL cells was positively associated with the corresponding transcripts sample-wise expression as compared with IR and transcript expression in NBCs (Figures 3B and 3C). However, the association between IR and transcript expression observed, comparing unmutated-IGHV CLL cases (HR) with NBCs had a statistically higher significance ($p < 0.05$) than the association kept comparing mutated IGHV CLL cases (LR) with NBCs. Further, the degree of association was increased in IGHV unmutated (poor prognosis) cases.

Analysis of biological pathways and molecular functions altered due to IR between CLL vs. NBC in transcripts with maximum IR

Next, we analyzed the top 25% transcripts from Figure 1A with high IR in CLL compared with NBCs (Table S2). We observed several different pathways affected by IR (Figure 4A), including extracellular matrix including collagen organization, inflammation mediated by chemokine and cytokine, hemostasis, antigen processing (T cell activation), immune-regulatory interactions between a lymphoid and non-lymphoid cell, and cellular response to stress. However, we found RNA processing, splicing, and gene expression pathway as the major ones where 30% of the transcript input belongs to this pathway. In summary, the IR-containing transcripts were enriched for biologically important pathways in CLL cells.

We analyzed the top 25% of transcripts for molecular function (MF) and assessed the effect of IR on molecular function in CLL cells. We observed several different MFs affected due to high IR in CLL/NBC (Figure 4B). Among the top MFs were protein binding, ion binding, nucleic acid binding, hydrolase activity, and transferase activity.

Network analysis

We analyzed RNA-seq data obtained from 98 CLL patients and nine NBCs using Reactome Functional Interaction (FI) network database, a highly reliable, manually curated pathway-based protein functional interaction network; this analysis allowed us to obtain protein

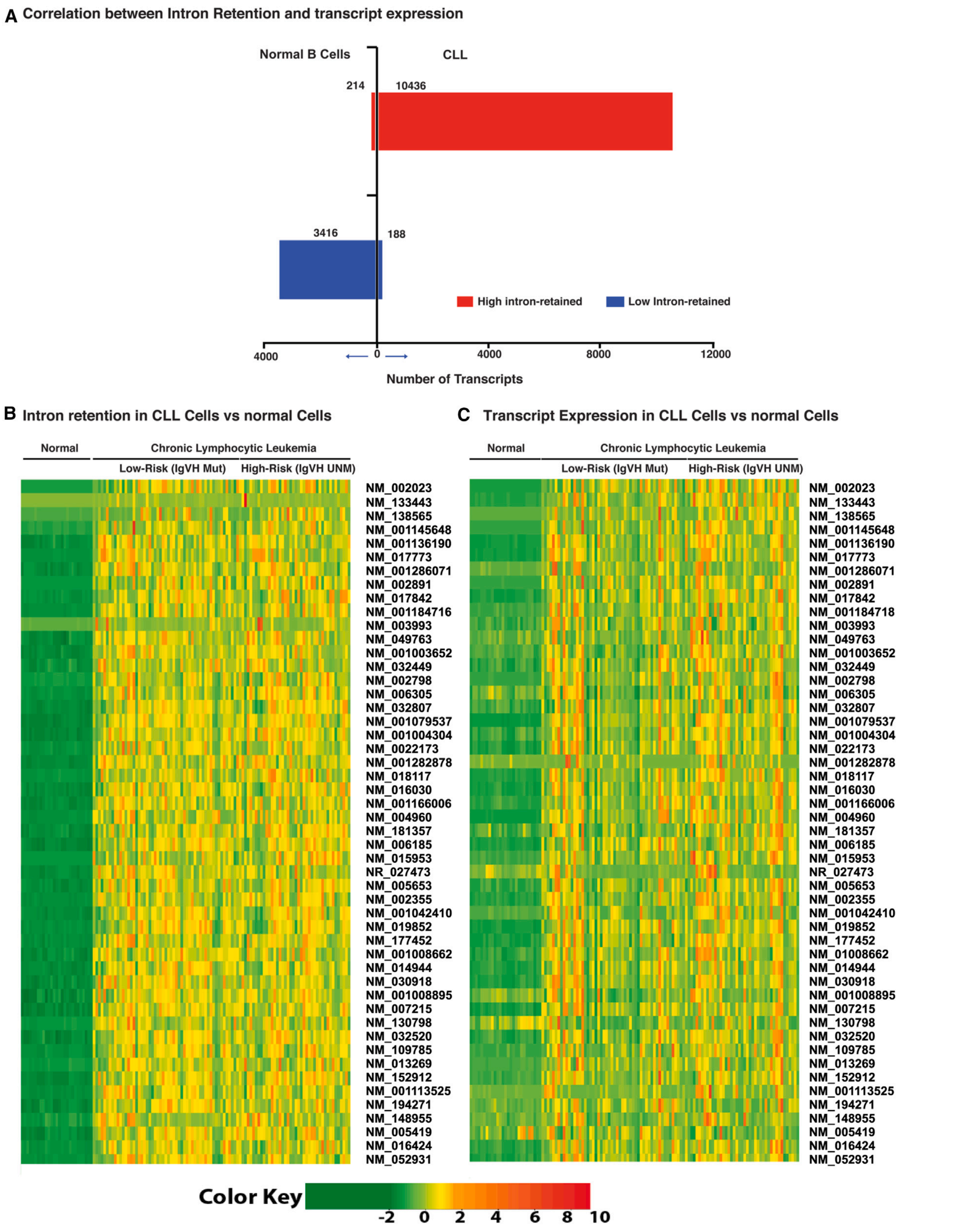

(legend on next page)

interaction data for 9,821 genes. Protein interaction networks were used to assign sets of genes to discrete subnetworks.

Cytoscape V3.4 software enables us to identify the first neighbors of SF3B1 and visualize the subnetwork centered on SF3B1. In this subnetwork, the color of the nodes represents the log2 ratio of IR in CLL cells vs. in NBCs (Figure 4C).

Furthermore, the network analysis provided insight into the pathways that emerged due to high IR in NBCs: the immune system, metabolism of RNA, DNA replication, cell cycle, and metabolism of proteins (Figure S3). When analyzed in CLL cells, the network analysis provided insight into the pathways that emerged due to high IR in CLL cells: immune system, homeostasis, and signal transduction (Figure S4).

Validation of candidate transcripts for IR in CLL cells and NBC

To validate our findings from the high-throughput RNA-seq data, we chose several transcripts with high IR in CLL cells and selected a region showing the event; the area, including the intron, was amplified, and we found that in all CLL cells but not in NBCs the intron was present. We selected a few transcripts for validation that included novel as well as known transcripts in the context of CLL. These were androgen-dependent TFPI regulating protein (*ADTRP*), cytotoxic T-lymphocyte-associated protein 4 (*CTLA4*), fibromodulin (*FMOD*), guanylate cyclase 2C (*GUCY2C*), heparin sulfate-glucosamine 3- sulfotransferase 1 (*HS3ST1*), NUBP iron-sulfur cluster assembly factor 2, cytosolic (*NUBP2*), protein phosphatase 2 regulatory subunit B'beta (*PPP2R5B*), protein tyrosine phosphatase receptor type J (*PTPRJ*), proline-serine-threonine phosphatase interacting protein 1 (*PSTPIP1*), ribosomal protein L39 like (*RPL39L*), and THAP domain containing 8 (*THAP8*). The tracks of intron usage have been shown for the selected transcripts (Figures 5A, S6A, S6B, S7A, S8A, S9A, S10A, S11A, and S12A). One such example of the *CTLA4* gene has been shown for the intronic region retained in CLL cells but not in NBCs (Figure 5A). Using RT-PCR, validation of transcripts for *CTLA4*, *ADTRP*, *FMOD*, *HS3ST1*, *GUCY2C*, *RPL39L*, *THAP8*, *PPP2R5B*, *PSTPIP1*, *PTPR5*, and *NUBP2* was done in CLL-B as well as in NBC cells for IR (Figures 5B, S7B, S8B, S9B, S10B, S11B, and S12B). The IR ratio for *CTLA4* was 29.5 in CLL-B cells as compared with NBCs (Figure 5B). The housekeeping gene *GAPDH* was used as a loading control and run along with *HS3ST1*, *NUPB2*, and *THAP8* in the same set of experiments for CLL-B and NBCs (Figures S7B, S8B, and S12B). We were able to amplify and detect the intronic region for *CTLA4* in all CLL-B cells but not in the case of NBCs. Further, we validated FMOD, HS3ST1, and GUCY2C in NBC and CLL-B cells at the protein levels by western blot using the respective antibodies targeting FMOD, HS3ST1, and GUCY2C. We found that the qualitative expression of FMOD and GUCY2C was more in case of CLL-B cells as compared with the NBCs (Figure S13).

We validated C6orf105, which was later designated as the *ADTRP* gene (Figure 5B). The IR ratio between CLL-B cells and NBC was 25.6, indicating a significantly higher IR in CLL-B cells compared with NBCs. Another known gene was (*FMOD*), for which we found a significant increase in IR in CLL-B cells compared with NBC. Previously, *FMOD* was over-expressed in CLL-B cells compared with NBCs.[29–31] The ratio of IR in CLL-B cells/NBCs was 23.4, and it was also validated in CLL-B cells (Figure 5B). Overall, we observed aberrant splicing and in particular high IR in CLL-B cells compared with NBC in ~73% of the transcripts.

Upregulation of total SF3B1 and pSF3B1 in CLL cells

A large number of studies in solid tumors and hematological malignancies reported aberrant regulation of the spliceosome complex. Since SF3B1 is one of the significant spliceosome complex proteins associated with leukemia, we reasoned to study SF3B1, one of the spliceosome complex's significant proteins.[32] We found that the expression of SF3B1 and pSF3B1 was significantly higher in CLL-B cells compared with NBCs ($p < 0.0001$ and $p < 0.01$, respectively) (Figure 6A). However, no significant difference was observed between SF3B1 and pSF3B1 expression levels in NBCs.

Effect of anti-IgM on SF3B1 and pSF3B1 expression in CLL-B cells and NBC

We tested whether SF3B1 and pSF3B1 expression could be modulated by anti-immunoglobulin (Ig)M stimulation. After *in vitro* stimulation of CLL-B cells with anti-IgM for 15 min, no difference in SF3B1 expression was observed between IgM stimulated and unstimulated; the same effect was observed between stimulated and unstimulated NBCs. Also, no difference in SF3B1 expression was observed when we compared CLL-B cells; we made a comparison between Mut-*SF3B1* and Wt- *SF3B1* (Figure 6B). But, when we compared both groups of CLL-B cells (Mut-SF3B1 and Wt-SF3B1) with NBCs, higher expression of total SF3B1 was evident in CLL (p value <0.05).

Figure 3. The correlation between intron retention and transcript expression in various cell types

(A) A total of 16,725 transcripts that were accumulated differentially used-intron respectively in CLL (set-I) and NBC (set-II) at $p \leq 0.05$ (FDR = 5%) and among those the three subsets each of the sets corresponding to over-expressed (set-IA, set-IIA), under-expressed (set-IB, set-IIB), and non-differentially expressed (set- IC, set-IIC) transcripts consequently found in CLL and NBC at $p < 0.05$ (FDR = 5%). The chi-square p value for the 2×3 table is $\leq$1.0e−300. The bar graph represents two sets of bars, each for a fraction of high intron used in CLL (set-I) and NBC (set-II) cases. It is associated with upregulated (set-IA, set-IIA) and downregulated (set-IB, set-IIB) at transcription level. The 2×2 contingency table shows significant p value <1.0e−300 and ODDs = 886. (B) The Heatmap Z scores of TPM values for sample-wise intron retention in NBC and CLL from each of the top 200 transcripts selected based on high intron retention in CLL with fulfilling condition CLL > NBC at $p < 0.05$; FDR = 5%. (C) The Heatmap Z scores of TPM values for sample-wise transcripts expression in NBC and CLL from each of the top 200 transcripts selected based on high intron retention in CLL with fulfilling condition CLL > NBC at $p < 0.05$; FDR = 5%.

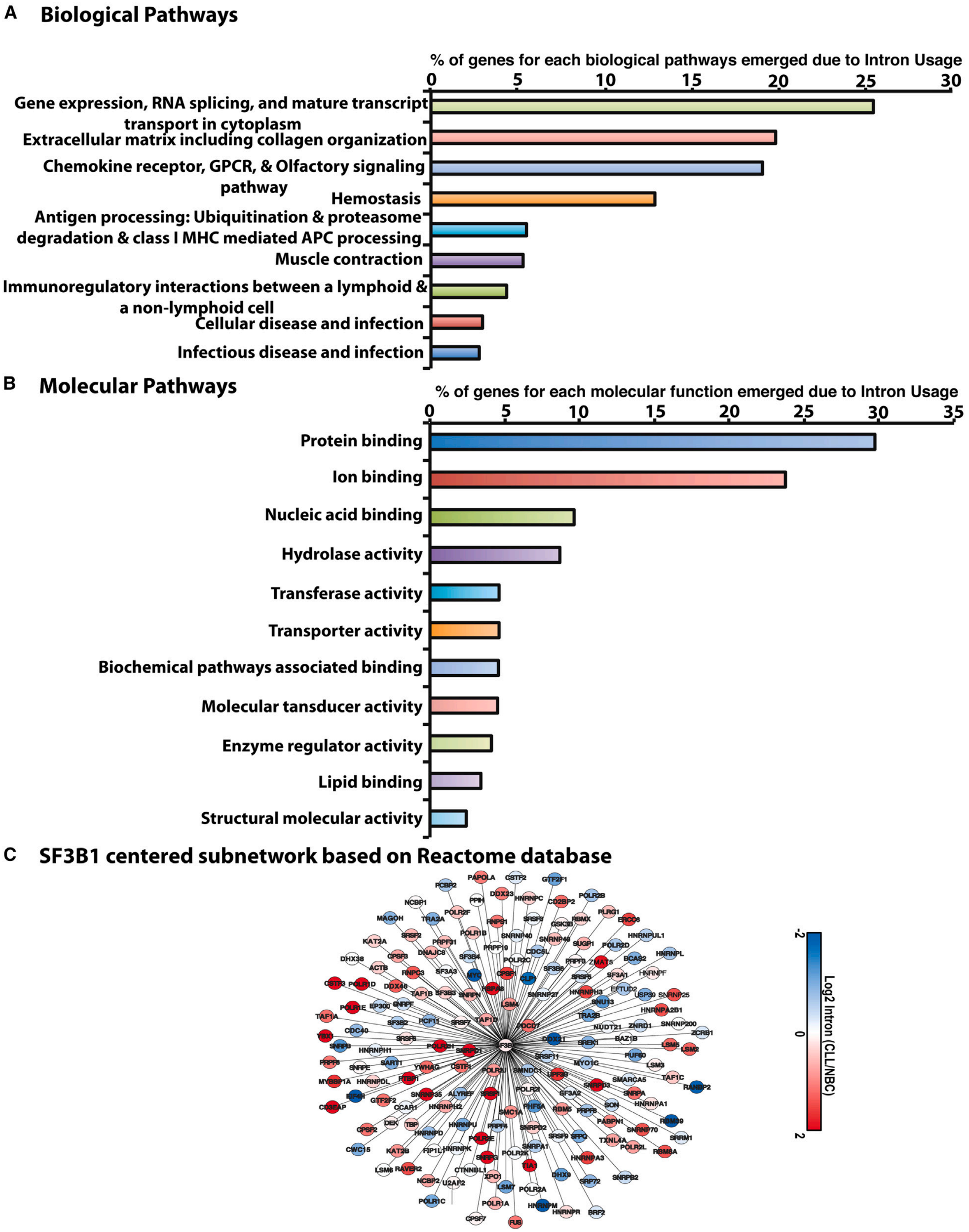

(legend on next page)

When the expression of pSF3B1 levels was observed between Wt-*SF3B1* vs. Mut-*SF3B1* CLL samples, no differences were observed; however, in both cases, there was a significant rise in protein expression after IgM stimulation. The pSF3B1 levels in both Wt-SF3B1 and Mut-SF3B1 had a marked overrepresentation compared with NBCs in both treatments, with IgM stimulation or without stimulation.

Modulation of expression of SF3B1 and effect on post-translational modification upon macrolide probe treatment in CLL-B cells

Since we observed IR in more than 70% of the transcripts, we hypothesized that the CLL spliceosome machinery could have an operation defect that makes it not complete the intron removal function as it should. Due to the lack of an ideal method to measure spliceosome activity in cells, we reasoned to use a previously reported macrolide molecule PLAD-B to modulate the splicing factor subunit SF3B1 (a driver protein for spliceosome activity) and to determine its phosphorylation. In particular, we interrogated the threonine amino acid site, which gets phosphorylated at site Thr313, reported to indicate active the spliceosome.[33]

We treated CLL-B cells with 100 nM of PLAD-B for 15, 60, 180, 360, or 960 min and interrogated for expression of total SF3B1 and pSF3B1. We found no significant change in total SF3B1 expression between untreated and treated CLL-B cells over time. However, in CLL-B cells, the level of pSF3B1 significantly decreased after 15 min of PLAD-B treatment. In contrast, the protein level of total-SF3B1 does not change, suggesting that the PLAD-B could control pSF3B1 form, and in turn, modulate the IR program of the transcriptome of CLL-B cells (Figure 6C). In contrast, no change in the expression of SF3B1 and pSF3B1 was noticed upon treatment with 10 μM of Fludarabine (F-ara-A), a conventional chemotherapy agent used in the clinic for CLL patient treatment.

DISCUSSION

Many studies have focused on transcriptome profiling of CLL-B cells in recent years. They have reported aberrant splicing events and mutations in genes such as *SF3B1*, *SRSF2*, *U2AF1*, and *U2AF2*, which belong to splicing machinery, i.e., spliceosome.[9,19,20,27,32,34,35] Furthermore, mutations in *SF3B1* are associated with poor prognosis in CLL patients.[36,37]

Alternative splicing is the process, which leads to diversity in the genome via the generation of novel isoforms for different genes controlled by the spliceosome complex. Several splicing events have been reported to be associated with other diseases, including cancers,[14,18,38–40] and other diseases like diabetes,[41] cardiac hypertrophy,[42] and amyotrophic lateral sclerosis.[43]

Many factors, such as the length of the exons and introns play a critical role in recognizing the splice site, and when small, it is most efficient.[44] The splicing events are based on the combination of introns and exons that are altered. In our study we investigated the degree to which IR was observed in the transcriptome of CLL-B cells compared with NBCs.

Evaluation of the total events that are part of ARS is very challenging, and because most ARS takes place with partial or complete involvement of the intron, we decided to align the reads, quantify the retained-intron or exon across the transcriptome, and calculate for each transcript the intron/exon ratio and use this value as an IR measure and a mathematical surrogate for ARS.

In an earlier RNA-seq study, we used fragments per kilobase of exon model per million mapped reads (FPKM) as the quantitative measure. Since then, there has been advancement in RNA-seq, and FPKM can be converted to TPM. Due to the inconsistences in amount samples introduced while quantifying the transcript abundance with reads per RPKM or FPKM, Wagner et al. introduced TPM and demonstrated that TPM respects the average invariance and eliminate statistical biases inherent in the RPKM or FPKM measures,[45] and overall the effect of normalization is smaller than variability in the amount of RNA per cell.[45–48] Hence, in the current study, we used TPM for RNA-seq analysis.

In recent years, among different types of splicing events, IR came into highlight because of its association with the inactivation of tumor suppressor genes and reports in other malignancies including lung adenocarcinoma,[15] breast cancer,[16] CLL,[18] hepatocellular carcinoma,[49] and even in normal tissues/cells like T cells,[50] normal granulocyte differentiation,[4] and neuronal development,[8] IR has been reported as a common mechanism for the inactivation of tumor suppressor genes.[40]

In this study, we observed that 66% of the total transcripts have high IR, and an increased level of transcript expression. Further, we assessed change in ratio of an abundance of IR in pre-mRNA vs. exon-using mRNA transcripts in CLL cells and found that high ratio of the pre-mRNA to mRNA is more prominent than

Figure 4. Biological pathway, molecular functions, and network analysis based on intron retention

(A) For biological pathway analysis, we took the top 25% of transcripts with high intron retention in CLL as compared with normal B cells ($p < 0.05$, FDR = 0.1). The gene symbols for corresponding transcripts were used as input and subjected for Reactome Pathway. The *p* value was adjusted using a Bonferroni correction. The x axis shows the % of the genes enriched and y axis shows the description of the pathways. (B) Molecular function analysis was done for the top 25% of transcripts with high intron retention CLL/NBC Network analysis by selecting transcripts with high intron retention in CLL as compared with normal B cells using WebGestalt. The height of the bar represents the % of genes observed in the category. (C) Subnetwork centered on SF3B1 and its first neighbors. The SF3B1 centered subnetwork was based on Reactome FI network database and cytoscape V3.4 was used to identify the first neighbors of SF3B1. Nodes and links represent genes and functional interactions, respectively. The color of each node scale with log2 intron retention ratio in CLL cells vs. NBCs as indicated in the scale at the bottom.

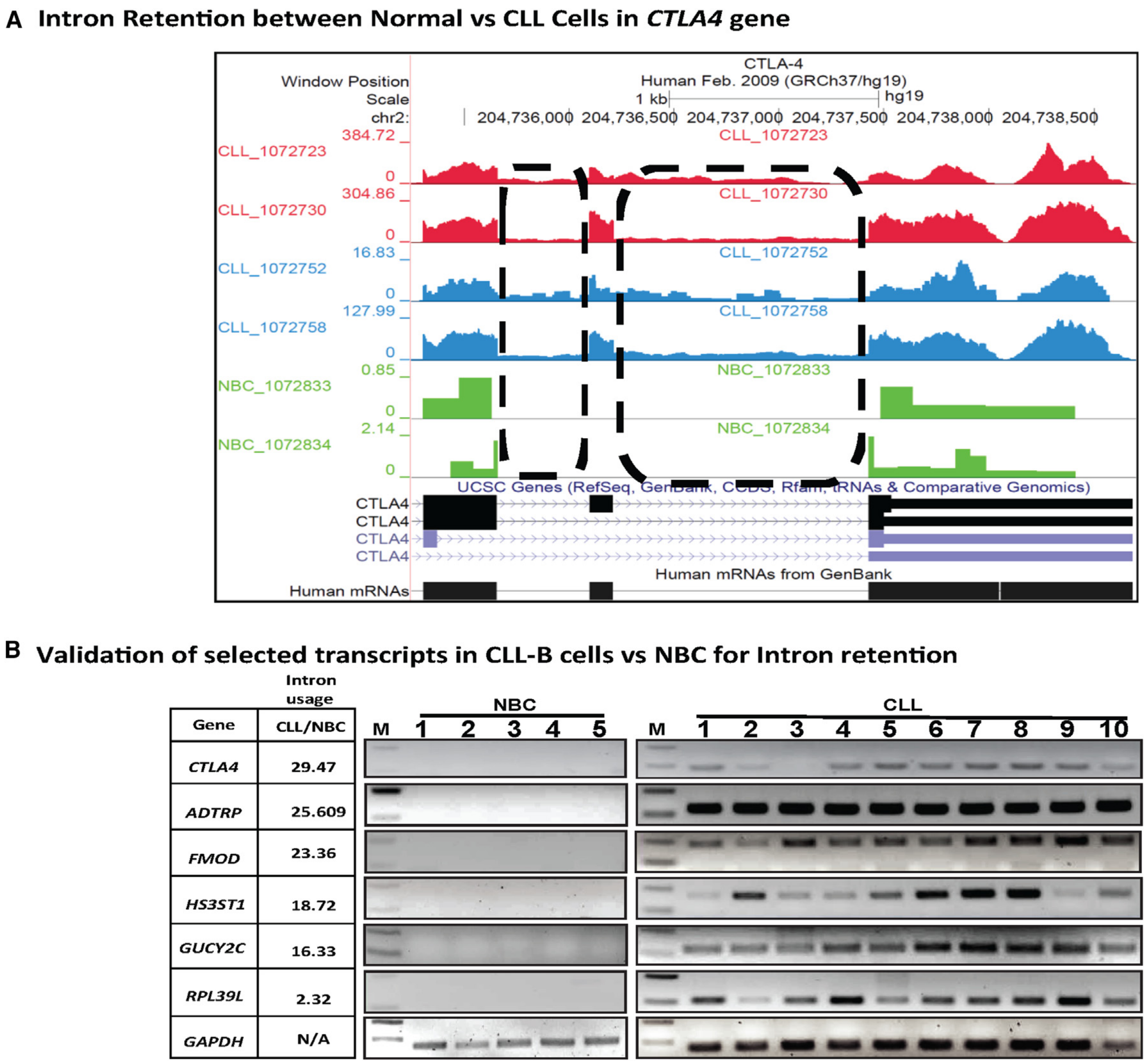

Figure 5. Selection and validation of transcripts for intron retention using RT-PCR

(A) RNA-seq read mapping (tracks) of the *CTLA4* gene from the UCSC reference genome (hg19), six representative samples belonging to high- or low-risk CLL cells, or normal B cells are shown. The intron retention tracks are shown in green (for normal B cells), blue (low-risk CLL), and red (high-risk CLL) colors. It is clear that the reads map to the intronic region of *CTLA4* transcript as annotated in the UCSC database. The absolute read counts for each sample are indicated on the y axis. (B) For validation, RNA was isolated from pure CLL-B cells or NBC and after DNase I digestion cDNA was prepared. Selected intronic region for assessment of intron retention were amplified using RT-PCR for *PPP2R5B*, *ADTRP*, *RPL39L*, *HS3ST1, CTLA4*, *FMOD*, and *GUCY2C* transcripts. *GAPDH* was used as control for normalization/loading of RNA.

low ratio, which globally suggests that highly accumulated pre-mRNA transcripts may downregulate expression of mRNA in CLL-B cells.

Further, among top IR harboring transcripts, RNA processing and splicing were the significant pathways represented in our findings. There are reports of genes with retained introns in concordance with our data, where an increase in transcript expression was observed for SLC25A37, *SPTA1,* and *SF3B1* genes.[51]

For all the six genes validated and analyzed, we found that none of the NBC samples showed intron region presence, whereas all the CLL samples showed presence of the intronic fragment indicating that IR occurs predominantly in CLL-B cells.

Among known genes, we found cortactin gene for which there was high IR as well as an increase in the transcript expression. *CTTN* has been reported in association with CLL with significantly increased mRNA expression in CLL-B cells compared with NBCs.[52] We validated cytotoxic T-lymphocyte-associated protein 4 (*CTLA4*) and Fibromodulin (*FMOD*) transcripts, which have been reported to be expressed in CLL. Another example of a known gene was *FMOD*. We found high IR and overexpression for *FMOD* in CLL specimens has been described previously.[17] In a recent study, *FMOD* was one of

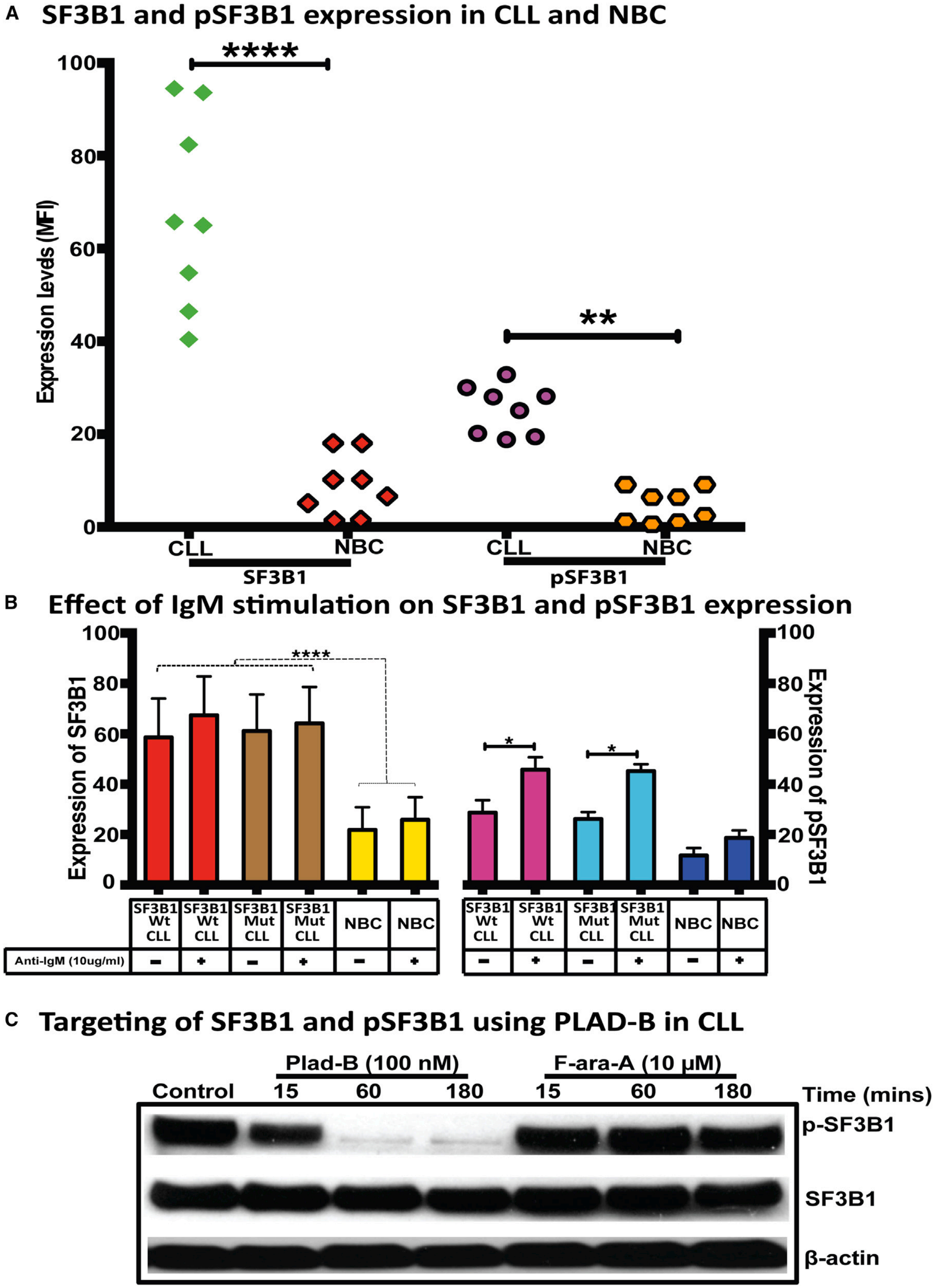

(legend on next page)

the seven genes included in an expression panel that was reliably able to distinguish CLL-B cells' clonal expansions from normal B-lymphocytes. *FMOD* has been reported to be upregulated and exclusively expressed in CLL and mantle cell lymphoma.[53]

Another gene *CTLA4* that code for cytotoxic T-lymphocyte-associated protein 4 (*CTLA4*) the ratio of IR ratio was 29.5 in CLL-B as compared with NBCs. *CTLA4* is also known as CD152 and has been reported in $CD4^+$ and $CD8^+$ T cells. It is a surface protein, which bears one transmembrane (TM) domain and a signal peptide. We were able to amplify *CTLA4* intronic region in all CLL-B but not in NBCs. *CTLA4* has been reported to be downregulated in CLL samples.[54] CTLA4 consists of two ligands present on B cells: CD80 (B7-1) and CD86 (B7-2), and has robust affinity toward these, and hence by binding to CD80, it can lead to inhibition of the T cell activation through AKT phosphorylation.[55,56] We were able to amplify the intronic region in all CLL samples but not detected in NBCs. We found that none of the NBC samples showed intron region presence. In contrast, all the CLL samples showed the intronic fragment's presence, indicating that IR occurs predominantly in CLL-B cells.

A widespread IR occurrence suggested an aberrant splicing pattern in CLL cells, further suggesting abnormal spliceosome activity. There has been an association between poor prognosis of CLL disease and Mut-*SF3B1*. Still, in this work, we found no significant IR difference between Mut-*SF3B1* and Wt-*SF3B1* harboring CLL-B cells, suggesting that the Mut-SF3B1 does not significantly influence the change of IR in CLL-B cell transcriptome. This may indicate that the K700E and other mutations assessed here are not related to the overall IR effect in CLL-B cells. In agreement with previous findings,[37] mutations in SF3B1 associated with poor outcome induced subtle but broad changes in gene expression across multiple pathways with no relation with spliceosome activity.

The findings in this study are in agreement with the previously published studies.[36,57] We reported an increased expression of *SF3B1* transcript and SF3B1 protein in CLL-B cells compared with NBCs. Still, there is no clear evidence whether it is because of chromatin hypomethylation of *SF3B1* or hyper-phosphorylation of threonine amino acid residues of SF3B1 at the 313rd position.[57] The *SF3B1* mutant cases have been observed to bear detectable levels of *SF3B1* transcript, which indicates the possibility that SF3B1 has a gain of function,[36,37,58] which explains that even Mut-SF3B1 also has overexpression of SF3B1 protein as compared with NBC. The transcript expression of SF3B1 has been reported previously as upregulated in CLL-B cells compared with NBCs.[36,59]

Dysregulated IR events in CLL-B cells as compared with NBCs indicate that these aberrant IR events could be associated with abnormalities in the spliceosome machinery in CLL-B cells. Part of the evidence for that is the pSF3B1 activity due to 313 threonine residue, which is found only in a catalytically active spliceosome form associated with chromatin and where ~80% of the pre-mRNA splicing occurs.[33] Moreover, our results suggest that there are significant abnormalities in IR/AS in cancer cells compared with their normal counterparts and highlight this pathway's role in carcinogenesis and potentially unveiling future therapeutic targets based on transcriptome analysis and gene expression.

After targeting the spliceosome with the SF3B1 macrolide inhibitor PLAD-B, we observed downregulation of pSF3B1 but no changes in total SF3B1 expression. The effect on pSF3B1 expression caused by PLAD-B appears to be highly specific as cytotoxic agents like F-ara-A did not induce pSF3B1 downregulation despite triggering cell death and even when this compound was used at supra-physiological concentrations. These data provide further support of the relevance of pSF3B1 and its role in splicing activity, cancer cell survival, and the mechanism of action of spliceosome inhibitors such as PLAD-B.

PLAD-B effectively induces cell death effectively in Mut-*SF3B1* or Wt-SF3B1 harboring CLL-B cells, which is intriguing. One possible reason could be the location of *SF3B1* mutations as in myelodysplastic syndrome, these are located in a close cluster, but in CLL, SF3B1 mutations are spread in the heat-repeat region.[60,61] Our study reported many non-coding RNAs (ncRNAs) with high intron usage, but their relevance concerning CLL is unknown. Interestingly, many RNAs that have been identified belonging to ncRNAs provide evidence on the translation of products in the form of peptides from the antisense transcripts and introns.[62] For future directions, we plan to study intron usage for transcripts, which retain the introns, harbor the premature termination codon, and examine how it targets the mRNA for nonsense-mediated decay. These studies could be critical as post-transcriptional mRNA processing. The stability of the transcripts is emerging as significant modes of gene regulation in cancer, even though little is known about how leukemic/cancerous cells control mRNA processing or target mRNAs for degradation.

Figure 6. Effect of IgM stimulation on SF3B1 and pSF3B1 expression in normal and CLL cells

(A) Normal B cells or CLL-B cells were stained for intracellular staining using anti-SF3B1 and anti-phospho-SF3B1 antibodies for total and phospho-SF3B1 protein using flow cytometry. (B) Normal B cells or CLL-B cells were stimulated with anti-IgM (10 μg/mL) for 15 min followed by intracellular staining using anti-SF3B1 and anti-phospho-SF3B1 antibodies for total and phospho-SF3B1 using flow cytometry. All the samples were run in duplicate and the data are presented with the means and their respective SDs. Statistical significance was determined by using Bonferroni correction test for multiple comparison test, where *, **, ***, **** represent $p < 0.05$, $p < 0.01$, $p < 0.001$, and $p < 0.0001$, respectively. (C) A total of 5 million CLL-B cells were incubated overnight and treated with 100 nM of Pladienolide-B (PLAD-B) or 10 μM of Fludarabine (F-ara-A) for 15, 60, and 180 min. Post-incubation of splicing modulators or chemotherapy, cells were harvested and lysed using modified RIPA. A total of 200 μg of protein was run on SDS-PAGE and subjected for western blot. Antibodies against SF3B1, and phospho-SF3B1 were used for assessing total and phospho-SF3B1 levels. β-actin was used as a loading control, while cells incubated in media only were used as the negative control (−).

In summary, our results suggest a crucial role of IR in CLL, IR/AS patterns, and pathways affected by these transcriptome alterations, and the potential impact of this in leukemia's pathophysiology. More importantly, we hypothesize that by extension, there are broader implications of our findings implicating IR/AS in the process of carcinogenesis in general. Furthermore, we found that pSF3B1 de-phosphorylation mediated by SF3B1 inhibitor (PLAD-B) is critical in the mechanism of these compounds' action and provides evidence that increased signaling through the spliceosome machinery components may be responsible for the IR/AS transcriptome alterations present in CLL. We showed that clearly there is significantly higher abundance of retained introns in CLL-B cells. The pathway analysis as well as macrolide treatment provided evidence in favor of the dysregulated RNA splicing machinery that could be targeted in CLL. These findings have the potential to be extrapolated in other malignancies as well. The role of not only IR, but also of other AS events could be of clinical significance in CLL, therefore that could be an area for further investigation to decipher the role of ARS in leukemogenesis. Our study provides evidence that there is correlation between transcript and protein levels as the validation using western blot technique for FMOD, HS3ST1, and GUCY2C1 showed that the expression of protein was qualitatively elevated in CLL-B cells as compared with NBCs. Our findings are in concordance with an earlier study where Pearson correlation at the transcript and protein levels was 0.74, 0.74, and 0.56 for *FMOD*, *GUCY2C*, and *HS3ST1* genes, respectively, suggesting that if there is an expression of transcript, we can expect protein expression as well.[63] Furthermore, other studies have reported that intron-retained mRNAs with premature termination codons (PTCs) are translated into new protein isoforms.[64] As other studies have reported that intron-retained mRNAs with PTCs are translated into new protein isoform ARS events in the context of leukemogenesis or carcinogenesis (in solid tumors), there is a need for large-scale integration of OMICS studies coupled with functional assays to address the question of how these events affect the process of leukemogenesis/carcinogenesis in different malignancies, including CLL.

MATERIALS AND METHODS

This study was conducted in compliance with the Declaration of Helsinki and approved by the Institutional Ethics Committee of the Moores Cancer Center, University of California, San Diego (CA), USA.

Strategy to identify the IR events in CLL vs. NBC samples

The RNA-seq data were obtained from the EGA (http://www.ebi.ac.uk/ega).[65] These data come from 98 CLL patients and nine NBC samples, and can be found in the following link on EGA (Study ID: EGAS00001000374).[27] In this study we used these datasets to study the significance of IR in CLL cells and NBCs. The workflow for the study shows different steps taken for executing the RNA-seq analysis on this dataset (Figure 1).

The details of the CLL samples used for RNA-seq analysis are provided in Table S1. The details of the primers used for detecting mutations in SF3B1 gene are provided in Table S3. The list of the patients with only *SF3B1* mutations is provided in Table S4.

The samples were classified into two risk groups: high risk and low risk based on IGHV mutational status. CLL patients with leukemic cells expressing unmutated IGHV (U-CLL) were classified as high risk (HR) and patients with CLL cells expressing mutated IGHV (M-CLL) as low risk (LR). The Illumina platform was used to sequence RNA collected from these CLL samples. The obtained reads were mapped to the Ensembl GRCh37.62 B (hg19) reference genome using RNA sequence aligner Tophat that aligns the reads across splice junctions independently of gene annotations.[66,67] The transcriptome-wide data were analyzed using TPM analysis. The transcripts that were expressed in at least 60% of both NBCs and CLL cell samples were subsequently analyzed for IR analysis.

Quantification of IR and isoform expression

For studying this problem, we downloaded paired-end reads fastq files of RNA-seq from the EGA (https://www.ebi.ac.uk/ega/home).[65] To perform the intron/exon quantification, we followed the guidelines published previously,[13,68] and detailed methods used in this study are provided in the supplemental material.

The reads from each sample were mapped onto the hg19 human genome using Tophat, version 2.0.10 with default parameters.[67] The output of that was an SAM file, which further converted to a BAM file using samtools (Version 1.3) *view* command and sorted the BAM file using and the samtools *sort* command.[66] Each sorted BAM file for NBC and CLL cells was used to calculate TPM values for each genomic feature (isoform, exonic and intronic regions) for every transcript. For the TPM calculation, we downloaded tablemaker and Ballgown Bioconductor packages.[69] Using the tablemaker package, we called first inbuilt Cufflinks command to reconstruct the individual transcripts and isoforms to estimate multi-mapped read count value for each of the genomic features from every BAM file and converted the output into a Ballgown readable formatted file. Later, using the Ballgown Bioconductor command, we loaded all readable formatted files of multi-mapped-corrected read values in two separate matrices in each case, for NBCs or CLL cells, corresponding to overlapped exonic and intronic regions for every transcript defined in hg19-GTF. We also quantified TPM expression values for every transcript and constructed two matrices for NBCs and CLL cells using the Ballgown command "transcript-gene-table" option applying Equation 1. We retained only those transcripts whose TPM expression values were >0 in more than 60% of NBC and CLL samples to ensure that the analyzed transcript expresses in the large majority of sample both in NBC and CLL cell conditions. The intron-less transcripts were removed from the hg19 GFF file. With this, we could capture 25,276 transcripts, which we used for the downstream analyses (Table S2).

We created an *in-house* program using python to calculate TPM values,[70] for every exonic and intronic region of the expressed

transcripts by applying the formulas 2 and 3 as shown in the supplemental material.

A 2D volcano plot was created to schematize the differential IR between CLL cells and NBCs, CLL cells harboring Mut-*SF3B1* and NBCs, CLL cells harboring Wt-*SF3B1* and NBCs, CLL cells harboring Mut-*SF3B1* and CLL cells harboring Wt-*SF3B1*, and finally M-CLL cells and U-CLL cells. The x axis displays the $\log_2$ fold change ratios between two different cell types (TPM [intron/exon]/TPM [intron/exon]) and the y axis corresponds to the $\log_{10}$ of the mean expression value (*p* value) (CLL-B [transcript expression]/NBC [transcript expression]).

Association between IR and transcript expression in CLL vs. NBC

To assess the differences in IR and transcript expression association between CLL cells and NBC, first we classified all the transcripts into two sets: (1) transcript with significantly high IR values in CLL cells and (2) transcript with significantly high IR values in NBC. For this, we compared the transcript-specific distribution of TPM in NBC-Intronic vs. CLL cells-Intronic values. We performed two separate one-sided Wilcoxon test, each for (CLL cells > NBCs) and (NBCs > CLL cells) conditions. The transcripts satisfying ($p < 0.05$; false discovery rate [FDR] = 5%) for CLL cells > NBC were separated as set-i and the same for the condition NBCs > CLL cells were separated for set-ii transcripts. Within each set, we further classified three sets of transcripts: (A) over-expressed, (B) under-expressed and (C) non-differentially expressed. For this, we used TPM values from the transcripts expression and performed one-sided Wilcoxon test each for (CLL cells > NBCs) and (NBCs > CLL cells) at $p < 0.05$ and FDR = 5% for set-A and set-B. For set-C we separately checked for expression difference for CLL cells ≠ NBC and NBC ≠ CLL cells at $p > 0.05$ and considered respectively as set-C for CLL cells and NBC.

After capturing several transcripts fulfilling the six sets, we first constructed 2 × 3 contingency table and performed a chi-square test. We also performed Fisher's 2 × 2 contingency table for high/low intron-used transcript vs. over/under-expression and recorded ODDs score.

To demonstrate sample-wise association study between high intron-retaining transcript in CLL cells with expression, we selected the top 200 transcripts from the set-IA based on ranked *p* value and captured the TPM values for CLL intron and CLL transcript from every CLL sample and also the TPM values for NBC intron and NBC transcript for each of the 200 NBC transcripts. Later we converted the TPM values into *Z* scores and plotted them into heatmaps using an in-house-built R-script. The heatmap column was sorted: first, NBC samples, second, M-CLL samples, and last U-CLL samples.

Analysis of biological pathways and MFs affected due to IR in CLL vs. NBC

We used the top 25% of transcripts with high IR in CLL cells as compared with NBCs. The biological pathway enrichment was done with the PANTHER (Protein Analysis Through Evolutionary Relationships) classification system. Top 25% of transcripts were used with significantly high IR in CLL cells compared with NBCs ($p \leq 0.05$; FDR = 0.1). We selected the transcript gene symbols used those as input, chose "Panther Overrepresentation Test" and picked Reactome Pathway as the annotation set. The *p* value was adjusted using a Bonferroni correction.[71,72]

Molecular function enrichment was done using WEB-based Gene Set Analysis Toolkit (WebGestalt, http://www.webgestalt.org).[73]

Network analysis

We used the Reactome FI network database, a highly reliable, manually curated pathway-based protein functional interaction network to analyze RNA-seq data obtained from 98 CLL patients and nine NBCs.[74] Reactome FI network database identified protein interaction data for 9821 genes. Protein interaction networks were used to assign sets of genes to discrete subnetworks.[75] A subnetwork is defined as a graphical representation of genes, represented as nodes, and functional interactions represented as connecting lines between nodes. The color of the nodes represents the log2 ratio of IR in CLL vs. in NBCs. A version V3.4 of Cytoscape was used to identify and visualize the first neighbors of SF3B1 in the subnetwork.[76]

B cell isolation

Peripheral blood mononuclear cells (PBMCs) derived from CLL cells were obtained from the CLL tissue bank of the Moores Cancer Center, University of California, San Diego (UCSD), La Jolla. Upon confirmation of the diagnosis of CLL,[77] patients provided written informed consent for blood sample collection on a protocol approved by the Institutional Review Board of UCSD, following with the Declaration of Helsinki.[78] NBCs were purified from buffy coats of healthy volunteer donors. Positive isolation with Dynabeads CD19 pan B (Life Technologies) and DETACHaBEAD CD19 (Life Technologies) were used to achieve more than 95% purity by flow cytometry analysis.

CLL cells

Chronic lymphocytic leukemia B cells (CLL cells) were either used as pure (purified for profiling using negative selection kit or studied by gating on double-positive cells for CD19/CD5 surface markers.[23]

SF3B1 sequencing for characterization of CLL samples to classify as Mut- SF3B1 or Wt-SF3B1.

For profiling of the CLL-B cells to know the *SF3B1* mutational status, we took 5e106 CLL-B cells and processed them for DNA isolation as described previously.[34] The DNA amplification for different *SF3B1* mutations focused on exons 14 and 15 was carried out using primers listed in Table S3. The details of the CLL samples used for SF3B1 and pSF3B1 profiling along with their *SF3B1* mutational status are provided in Table 1. Also, the *SF3B1*-mutated CLL samples used for RNA-seq analyses are listed in Table S4.

Table 1. CLL patients with *SF3B1* mutations used in the study

Sample ID	Type of *SF3B1* mutation	Type of *SF3B1* mutation
CLL101	Heterozygous	K700E
CLL102	Heterozygous	K700E
CLL103	Heterozygous	E622D
CLL104	Heterozygous	N626Y
CLL105	Heterozygous	K700E
CLL106	Heterozygous	K700E
CLL107	Heterozygous	G740E
CLL108	Heterozygous	K700E
CLL109	Heterozygous	K700E
CLL110	Heterozygous	R625C

Reverse-transcription PCR

For validation of candidates for IR, CLL, or NBCs, 5e106 cells/well were subjected to RNA isolation. Total RNA was isolated using the total RNA isolation kit from Life Technologies (Grand Island, NY, USA). For IR validation studies, the potential source of false positives could be the traces of DNA present in CLL or NBC samples after RNA isolation; isolated RNA samples were treated with DNase I to remove DNA contamination and then cDNA was prepared as described previously.[23] The list of primers used in the study for validation of IR for transcripts of different genes is shown in Tables 2 and S5.

PCR conditions were as follows: 95°C for 3 min; 35 cycles of 95°C for 30 s; 55-58°C (dependent on transcript-specific primers) for 30 s, and 72°C for 45 s; followed by 72°C for 5 min using a PTC-100 thermocycler (MJ Research). PCR products were separated on a 2% agarose gel and stained with ethidium bromide.[23]

Expression of SF3B1 and phospho-SF3B1 in CLL samples

The CLL cells and NBCs were seeded the day before the experiment and cultured overnight. Before plating, the DMSO content was removed by making the total volume to 10 mL by adding Hank's balanced salt solution and the cells were centrifuged at 300 × *g* for 5 min at room temperature. The supernatant was discarded, and the cells were re-suspended in appropriate volume to have at least 1e6 cells/50 μL/well.

The cells were plated and incubated with 10 μg/mL of anti-IgM (catalog # 2022-08, Southern Biotech) for 15 min at 37°C.[79,80] Upon incubation, the cells were centrifuged immediately at 4°C to stop the reaction and washed twice with FACS buffer. The cells were labeled with CD19/CD5 (for CLL cells) and CD19/CD20 (for NBCs) and incubated for 30 min at 4°C. The cells were incubated in pre-warmed cytofix buffer at 37°C for 10 min before use and centrifuged at 300 × *g* for 5 min; the supernatant was removed. The cells were disrupted by pipetting back and forth and permeabilized using Phosphoflow Perm buffer by incubating for 30 min on ice. The cells were washed twice with FACS buffer and centrifuged at 300 × *g* for 10–15 min to remove the supernatant. The PBMCs isolated from human blood were processed for CD19/CD20 staining at 4°C. The cells were washed twice with FACS buffer and fixed immediately using BD Phosphoflow Perm Buffer (Catalog # 558052) for 10 min at 37°C.

Post-incubation, cells were centrifuged and after decanting the supernatant the cells were fixed with BD PhosphoflowTM Perm buffer II (catalog # 558052) for 30 min on ice. The cells were centrifuged at 300 × *g* for 5 min and the supernatant was discarded. Further, cells were washed twice with FACS buffer. The Fc receptor (FcR) was blocked by using FcR blocking agent (catalog # 130-059–901, Miltenyi Biotec) in a ratio of 1:25 at room temperature for 10 min. The cells were treated using 1:200 dilutions of rabbit anti-Human SF3B1 (D7L5T, catalog #14434, Cell Signaling Technology), rabbit anti-human phosphor-SF3B1 (Catalog # 25009S, Cell Signaling Technology), or normal rabbit IgG (catalog # 2729, Cell Signaling Technology) as a non-specific isotype control for 30 min at room temperature. The cells were washed twice with FACS buffer and incubated with Alexa Fluor 488 Conjugated goat anti-rabbit IgG (H + L), F(ab') fragment (catalog #4412, Cell Signaling Technology) with a dilution of 1:50 for 30 min at room temperature. The cells were washed twice with FACS buffer and subjected to run on the flow cytometer. The data analysis was carried out using FlowJo software. All the samples were run in duplicate.

Western blot analysis

CLL cells were treated with 100 nM of PLAD-B for 15, 60, and 180 min. The lysates were prepared using a modified RIPA buffer. Untreated CLL cells were used as a control. The samples containing at least 20 μg of total protein were subjected to 4%–20% Criterion Precast Gel (Bio-Rad) SDS-PAGE, followed by transfer using polyvinylidene difluoride (PVDF) membrane (Millipore). After blocking with 5% bovine serum albumin in TBST (20 mM Tris●HCl, 137 mM NaCl, 0.1% Tween 20 pH 7.6), the membrane was incubated with primary antibody overnight at 4°C.[24] The following primary antibodies were used: rabbit monoclonal anti-SF3B1 at dilution 1:1,000 (14434, clone D7L5T, Cell Signaling Technology), mouse anti-phospho-SF3B1 (Anti-pSF3B1) at dilution 1:1,000 (clone, D8D8V, site: Thr313, catalog # 25009S, Cell Signaling Technology), rabbit anti-GUCY2C at 1:5,000 dilution (Catalog # 29881, Cell Signaling Technology), mouse anti-FMOD at 1:5,000 dilution (Catalog # SAB1409025, Sigma-Aldrich), sheep anti-HS3ST1 at 1:5,000 dilution (Catalog # AF5968, R&D Systems) and rabbit anti-β-actin at dilution 1:5,000 (Catalog # 4967, Cell Signaling Technology). After washing twice with TBST, the membranes were incubated with horseradish peroxidase (HRP)-labeled anti-rabbit (Catalog # sc-2030, SantaCruz Biotechnology) or HRP-labeled anti-mouse (Catalog # sc-2031, Santa Cruz Biotechnology) or Anti-Sheep IgG (Catalog A3415, Sigma-Aldrich) secondary antibodies with a dilution of 1:5,000 dilution for 1 h at room temperature in TBST with 5% skimmed milk. Protein-antibody complex signals were detected by exposing the X-ray films (Catalog #E3018, HyBlot CL) after treatment with enhanced chemiluminescence (ECL) kit (catalog # 32106, Pierce Thermo Scientific).

Table 2. List of primers used in validation of intron usage for different genes in CLL-B and normal B cells

Gene symbol	HGNC approved Name/Gene symbol of the molecule (genomic region tested)	HGNC ID	HPRD ID	Refseq ID	Coordinates on UCSC genome browser	Primers	Primer sequence [5′ sequence 3′]	Tm (°C)	PCR product (bp)
ADTRP	Androgen-dependent TFPI regulating protein (chr6:11716510-11716630)	21214	12836	GenBank: NM_032744	chr6:11716510-11716529	FP	AATGGGTCTCTGTGGTGCTC	59	121
					chr6:11716611-11716630	RP	ACTAAGACCCGCTTGCCATT		
RPL39L	Ribosomal protein L39-like (chr3:186,847,868-186,848039)	17094	09610	GenBank: NM_052969	chr3:186,847,868-186,847,887	FP	TCCTTCAAGACAGCCCTTGC	56	172
					chr3:186,848,019–186,848,039	RP	TGCTTTGAAGGTGACATCGTG		
HS3ST1	Heparan sulfate (glucosamine) 3-O-sulfotransferase 1 (chr4:11,424,163-11,424,329)	5194	04455	GenBank: NM_005114	chr4:11,424,163-11,424,182	FP	GACAGGCAGAGACTCGGTT	57	167
					chr4:11,424,310-11,424,329	RP	TTGTGCCCTTGGAGACTCTG		
CTLA4	Cytotoxic T-lymphocyte-associated antigen 4 (chr2:203871925-203872008)	2505	00474	GenBank: NM_005214	chr2:203871925-203871944	FP	TGGTGCCTTCCTTGTCGAAG	53	84
					chr2:203871989-203872008	RP	AAGGCAAAACGTCCCAGTCA		
FMOD	Fibromodulin (chr1:203343741-203343920)	3774	*02591*	GenBank: NR_103757	chr1:203343741-203343760	FP	TAGGTGTGACCATGAGGGCT	56	180
					chr1:203343896-203343920	RP	GCTGCAACCTCACCTCATCT		
GUCY2C	Guanylate Cyclase 2C (chr12:14625022-14625136)	4688	*07529*	GenBank: NM_004963	chr12:14625022-14625041	FP	GTTCCCTACTGTTCCCAGGC	55	115
					chr12:14625117-14625136	RP	GGATCACAGCTTGGGGGAAA		
GAPDH	Glyceraldehyde-3-phosphate dehydrogenase (chr12:6536514-6536754)	4141	00713	GenBank: NM_002046	chr12:6536514-6536531	FP	5′ TGGTCACCAGGGCTGCTT 3′	57	151
					chr12:6536735-6536754	RP	5′ AGCTTCCCGTTCTCAGCCTT 3′		

FP, Forward Primer; RP, Reverse Primer.

Statistical analyses

Data were analyzed using GraphPad Prism 5.0 (GraphPad Software, Inc.). The error bars represent standard deviation (SD). Statistical differences for the mean values are indicated as follows: *, **, ***, and **** denote $p < 0.05$; $p < 0.01$; $p < 0.001$, $p < 0.0001$, respectively.

DATA AND CODE AVAILABILITY

All data generated and analyzed during our study are included either in the published article or supplemental data associated with the manuscript.

SUPPLEMENTAL INFORMATION

Supplemental information can be found online at https://doi.org/10.1016/j.omtn.2024.102202.

ACKNOWLEDGMENTS

We thank the EGA for providing us extensive large RNA-seq data set on CLL cell and NBC samples. The authors would like to thank for grant support: Lymphoma Research Foundation (LRF, grant #285871) to J.E.C., the National Institutes of Health (PO1-CA081534)-CLL Research Consortium Grant to T.J.K., J.E.C. and UC San DiegoFoundation Blood Cancer Research Fund (T.J.K.), and the Bailey Family Foundation (J.E.C.). M.K.K. is the recipient of the TARE fellowship (Grant # TAR/2018/001054) from the Science and Engineering Research Board (SERB), Department of Science and Technology, Government of India, and sanction #: 5/13/55/2020/NCD-III from the Indian Council of Medical Research (ICMR), New Delhi, India.

AUTHOR CONTRIBUTIONS

J.E.C., T.J.K., and M.K.K. conceived and guided the research. M.K.K., H.K., D.K., and J.V.L. designed the experiments, analyzed the data, and interpreted the results. M.K.K., D.K., J.V.L., C.I.A., E.M.G., R.V.A., C.I.A., N.M.V., D.A., S.H., S.M.M., V.F.F.J, L.M., and Z.Y. conducted the experiments, data analysis, and interpretation of the results. J.E.C., M.K.K., and H.K. wrote the manuscript. J.E.C., M.Y.C., L.Z.R., E.G., and T.J.K. provided patient samples and clinical and laboratory data. S.H., L.M.R., S.M.M., P.A.L., J.V.F.F., and E.M. discussed the results and editing of the manuscript critically. All of the authors read and approved the final manuscript.

DECLARATION OF INTERESTS

The authors declare no competing interests.

REFERENCES

1. Hewamana, S., and Dearden, C. (2011). Treatment options for high-risk chronic lymphocytic leukaemia. Ther. Adv. Hematol. *2*, 147–159.
2. Stilgenbauer, S., and Zenz, T. (2010). Understanding and managing ultra high-risk chronic lymphocytic leukemia. Hematology. Am. Soc. Hematol. Educ. Program *2010*, 481–488.
3. Wong, J.J.L., Gao, D., Nguyen, T.V., Kwok, C.T., van Geldermalsen, M., Middleton, R., Pinello, N., Thoeng, A., Nagarajah, R., Holst, J., et al. (2017). Intron retention is regulated by altered MeCP2-mediated splicing factor recruitment. Nat. Commun. *8*, 15134.
4. Wong, J.J.L., Ritchie, W., Ebner, O.A., Selbach, M., Wong, J.W.H., Huang, Y., Gao, D., Pinello, N., Gonzalez, M., Baidya, K., et al. (2013). Orchestrated intron retention regulates normal granulocyte differentiation. Cell *154*, 583–595.
5. Ergun, A., Doran, G., Costello, J.C., Paik, H.H., Collins, J.J., Mathis, D., and Benoist, C.; ImmGen Consortium (2013). Differential splicing across immune system lineages. Proc. Natl. Acad. Sci. USA *110*, 14324–14329.
6. Moore, M.J., Wang, Q., Kennedy, C.J., and Silver, P.A. (2010). An alternative splicing network links cell-cycle control to apoptosis. Cell *142*, 625–636.
7. Shaked, I., Zimmerman, G., and Soreq, H. (2008). Stress-induced alternative splicing modulations in brain and periphery: acetylcholinesterase as a case study. Ann. N. Y. Acad. Sci. *1148*, 269–281.
8. Yap, K., Lim, Z.Q., Khandelia, P., Friedman, B., and Makeyev, E.V. (2012). Coordinated regulation of neuronal mRNA steady-state levels through developmentally controlled intron retention. Genes Dev. *26*, 1209–1223.
9. DeBoever, C., Ghia, E.M., Shepard, P.J., Rassenti, L., Barrett, C.L., Jepsen, K., Jamieson, C.H.M., Carson, D., Kipps, T.J., and Frazer, K.A. (2015). Transcriptome sequencing reveals potential mechanism of cryptic 3' splice site selection in SF3B1-mutated cancers. PLoS Comput. Biol. *11*, e1004105.
10. Park, E., Pan, Z., Zhang, Z., Lin, L., and Xing, Y. (2018). The Expanding Landscape of Alternative Splicing Variation in Human Populations. Am. J. Hum. Genet. *102*, 11–26.
11. Bjorklund, S.S., Panda, A., Kumar, S., Seiler, M., Robinson, D., Gheeya, J., Yao, M., Alnaes, G.I.G., Toppmeyer, D., Riis, M., et al. (2017). Widespread alternative exon usage in clinically distinct subtypes of Invasive Ductal Carcinoma. Sci. Rep. *7*, 5568.
12. Zhao, S., Zhang, Y., Gamini, R., Zhang, B., and von Schack, D. (2018). Evaluation of two main RNA-seq approaches for gene quantification in clinical RNA sequencing: polyA+ selection versus rRNA depletion. Sci. Rep. *8*, 4781.
13. Gaidatzis, D., Burger, L., Florescu, M., and Stadler, M.B. (2015). Analysis of intronic and exonic reads in RNA-seq data characterizes transcriptional and post-transcriptional regulation. Nat. Biotechnol. *33*, 722–729.
14. Eswaran, J., Horvath, A., Godbole, S., Reddy, S.D., Mudvari, P., Ohshiro, K., Cyanam, D., Nair, S., Fuqua, S.A.W., Polyak, K., et al. (2013). RNA sequencing of cancer reveals novel splicing alterations. Sci. Rep. *3*, 1689.
15. Zhang, Q., Li, H., Jin, H., Tan, H., Zhang, J., and Sheng, S. (2014). The global landscape of intron retentions in lung adenocarcinoma. BMC Med. Genomics *7*, 15.
16. Dvinge, H., and Bradley, R.K. (2015). Widespread intron retention diversifies most cancer transcriptomes. Genome Med. *7*, 45.
17. Liao, W., Jordaan, G., Nham, P., Phan, R.T., Pelegrini, M., and Sharma, S. (2015). Gene expression and splicing alterations analyzed by high throughput RNA sequencing of chronic lymphocytic leukemia specimens. BMC Cancer *15*, 714.
18. Kashyap, M.K., Kumar, D., Villa, R., La Clair, J.J., Benner, C., Sasik, R., Jones, H., Ghia, E.M., Rassenti, L.Z., Kipps, T.J., et al. (2015). Targeting the spliceosome in chronic lymphocytic leukemia with the macrolides FD-895 and pladienolide-B. Haematologica *100*, 945–954.
19. Singh, B., and Eyras, E. (2017). The role of alternative splicing in cancer. Transcription *8*, 91–98.
20. Urbanski, L.M., Leclair, N., and Anczuków, O. (2018). Alternative-splicing defects in cancer: Splicing regulators and their downstream targets, guiding the way to novel cancer therapeutics. Wiley Interdiscip. Rev. RNA *9*, e1476.
21. Zhang, Z., Chen, S., Chen, S., Chen, G., Zhang, R., Li, J., and Qu, J. (2017). SF3B1 mutation is a prognostic factor in chronic lymphocytic leukemia: a meta-analysis. Oncotarget *8*, 69916–69923.
22. Te Raa, G.D., Derks, I.A.M., Navrkalova, V., Skowronska, A., Moerland, P.D., van Laar, J., Oldreive, C., Monsuur, H., Trbusek, M., Malcikova, J., et al. (2015). The impact of SF3B1 mutations in CLL on the DNA-damage response. Leukemia *29*, 1133–1142.
23. Dhar, S., La Clair, J.J., León, B., Hammons, J.C., Yu, Z., Kashyap, M.K., Castro, J.E., and Burkart, M.D. (2016). A Carbohydrate-Derived Splice Modulator. J. Am. Chem. Soc. *138*, 5063–5068.
24. Kumar, D., Kashyap, M.K., La Clair, J.J., Villa, R., Spaanderman, I., Chien, S., Rassenti, L.Z., Kipps, T.J., Burkart, M.D., and Castro, J.E. (2016). Selectivity in Small Molecule Splicing Modulation. ACS Chem. Biol. *11*, 2716–2723.

25. Burkart, M.D., LC, J.J., Jones, B.D., Mandel, A., Villa, R., Castro, J.E., Kashyap, M.K., and Kumar, D. (2017). Anti-cancer Polyketide Compounds. US Patent *1*.

26. Tang, Q., Rodriguez-Santiago, S., Wang, J., Pu, J., Yuste, A., Gupta, V., Moldón, A., Xu, Y.Z., and Query, C.C. (2016). SF3B1/Hsh155 HEAT motif mutations affect interaction with the spliceosomal ATPase Prp5, resulting in altered branch site selectivity in pre-mRNA splicing. Genes Dev. *30*, 2710–2723.

27. Ferreira, P.G., Jares, P., Rico, D., Gómez-López, G., Martínez-Trillos, A., Villamor, N., Ecker, S., González-Pérez, A., Knowles, D.G., Monlong, J., et al. (2014). Transcriptome characterization by RNA sequencing identifies a major molecular and clinical subdivision in chronic lymphocytic leukemia. Genome Res. *24*, 212–226.

28. Fernandez-Orth, D., Rueda, M., Singh, B., Moldes, M., Jene, A., Ferri, M., Vasallo, C., Fromont, L.A., Navarro, A., and Rambla, J. (2022). A quality control portal for sequencing data deposited at the European genome-phenome archive. Brief Bioinform. *23*, bbac136.

29. Mayr, C., Bund, D., Schlee, M., Moosmann, A., Kofler, D.M., Hallek, M., and Wendtner, C.M. (2005). Fibromodulin as a novel tumor-associated antigen (TAA) in chronic lymphocytic leukemia (CLL), which allows expansion of specific CD8+ autologous T lymphocytes. Blood *105*, 1566–1573.

30. Mikaelsson, E., Danesh-Manesh, A.H., Lüppert, A., Jeddi-Tehrani, M., Rezvany, M.R., Sharifian, R.A., Safaie, R., Roohi, A., Osterborg, A., Shokri, F., et al. (2005). Fibromodulin, an extracellular matrix protein: characterization of its unique gene and protein expression in B-cell chronic lymphocytic leukemia and mantle cell lymphoma. Blood *105*, 4828–4835.

31. Farahi, L., Ghaemimanesh, F., Milani, S., Razavi, S.M., Hadavi, R., Bayat, A.A., Salimi, A., Akhondi, M.M., and Rabbani, H. (2019). Anchored Fibromodulin as a Novel Target in Chronic Lymphocytic Leukemia: Diagnostic and Therapeutic Implications. Iran. J. Immunol. *16*, 127–141.

32. Wang, L., Lawrence, M.S., Wan, Y., Stojanov, P., Sougnez, C., Stevenson, K., Werner, L., Sivachenko, A., DeLuca, D.S., Zhang, L., et al. (2011). SF3B1 and other novel cancer genes in chronic lymphocytic leukemia. N. Engl. J. Med. *365*, 2497–2506.

33. Girard, C., Will, C.L., Peng, J., Makarov, E.M., Kastner, B., Lemm, I., Urlaub, H., Hartmuth, K., and Lührmann, R. (2012). Post-transcriptional spliceosomes are retained in nuclear speckles until splicing completion. Nat. Commun. *3*, 994.

34. Schwaederle, M., Ghia, E., Rassenti, L.Z., Obara, M., Dell'Aquila, M.L., Fecteau, J.F., and Kipps, T.J. (2013). Subclonal evolution involving SF3B1 mutations in chronic lymphocytic leukemia. Leukemia *27*, 1214–1217.

35. Quesada, V., Conde, L., Villamor, N., Ordóñez, G.R., Jares, P., Bassaganyas, L., Ramsay, A.J., Beà, S., Pinyol, M., Martínez-Trillos, A., et al. (2011). Exome sequencing identifies recurrent mutations of the splicing factor SF3B1 gene in chronic lymphocytic leukemia. Nat. Genet. *44*, 47–52.

36. Wan, Y., and Wu, C.J. (2013). SF3B1 mutations in chronic lymphocytic leukemia. Blood *121*, 4627–4634.

37. Wang, L., Brooks, A.N., Fan, J., Wan, Y., Gambe, R., Li, S., Hergert, S., Yin, S., Freeman, S.S., Levin, J.Z., et al. (2016). Transcriptomic Characterization of SF3B1 Mutation Reveals Its Pleiotropic Effects in Chronic Lymphocytic Leukemia. Cancer Cell *30*, 750–763.

38. Hsu, T.Y.T., Simon, L.M., Neill, N.J., Marcotte, R., Sayad, A., Bland, C.S., Echeverria, G.V., Sun, T., Kurley, S.J., Tyagi, S., et al. (2015). The spliceosome is a therapeutic vulnerability in MYC-driven cancer. Nature *525*, 384–388.

39. Wong, J.J.L., Au, A.Y.M., Ritchie, W., and Rasko, J.E.J. (2016). Intron retention in mRNA: No longer nonsense: Known and putative roles of intron retention in normal and disease biology. Bioessays *38*, 41–49.

40. Jung, H., Lee, D., Lee, J., Park, D., Kim, Y.J., Park, W.Y., Hong, D., Park, P.J., and Lee, E. (2015). Intron retention is a widespread mechanism of tumor-suppressor inactivation. Nat. Genet. *47*, 1242–1248.

41. Newman, J.R.B., Conesa, A., Mika, M., New, F.N., Onengut-Gumuscu, S., Atkinson, M.A., Rich, S.S., McIntyre, L.M., and Concannon, P. (2017). Disease-specific biases in alternative splicing and tissue-specific dysregulation revealed by multitissue profiling of lymphocyte gene expression in type 1 diabetes. Genome Res. *27*, 1807–1815.

42. Kim, T., Kim, J.O., Oh, J.G., Hong, S.E., and Kim, D.H. (2014). Pressure-overload cardiac hypertrophy is associated with distinct alternative splicing due to altered expression of splicing factors. Mol. Cells *37*, 81–87.

43. Wang, Q., Conlon, E.G., Manley, J.L., and Rio, D.C. (2020). Widespread intron retention impairs protein homeostasis in C9orf72 ALS brains. Genome Res. *30*, 1705–1715.

44. Amit, M., Donyo, M., Hollander, D., Goren, A., Kim, E., Gelfman, S., Lev-Maor, G., Burstein, D., Schwartz, S., Postolsky, B., et al. (2012). Differential GC content between exons and introns establishes distinct strategies of splice-site recognition. Cell Rep. *1*, 543–556.

45. Wagner, G.P., Kin, K., and Lynch, V.J. (2012). Measurement of mRNA abundance using RNA-seq data: RPKM measure is inconsistent among samples. Theory Biosci *131*, 281–285.

46. Conesa, A., Madrigal, P., Tarazona, S., Gomez-Cabrero, D., Cervera, A., McPherson, A., Szcześniak, M.W., Gaffney, D.J., Elo, L.L., Zhang, X., and Mortazavi, A. (2016). A survey of best practices for RNA-seq data analysis. Genome Biol. *17*, 13.

47. Li, B., and Dewey, C.N. (2011). RSEM: accurate transcript quantification from RNA-Seq data with or without a reference genome. BMC Bioinf. *12*, 323.

48. Hafemeister, C., and Satija, R. (2019). Normalization and variance stabilization of single-cell RNA-seq data using regularized negative binomial regression. Genome Biol. *20*, 296.

49. Zhou, J., Lai, P.B.S., and Tsui, S.K.W. (2015). Identification of a non-coding KLF4 transcript generated from intron retention and downregulated in human hepatocellular carcinoma. Int. J. Oncol. *47*, 1554–1562.

50. Cho, V., Mei, Y., Sanny, A., Chan, S., Enders, A., Bertram, E.M., Tan, A., Goodnow, C.C., and Andrews, T.D. (2014). The RNA-binding protein hnRNPLL induces a T cell alternative splicing program delineated by differential intron retention in polyadenylated RNA. Genome Biol. *15*, R26.

51. Pimentel, H., Parra, M., Gee, S.L., Mohandas, N., Pachter, L., and Conboy, J.G. (2016). A dynamic intron retention program enriched in RNA processing genes regulates gene expression during terminal erythropoiesis. Nucleic Acids Res. *44*, 838–851.

52. Gattazzo, C., Martini, V., Frezzato, F., Trimarco, V., Tibaldi, E., Castelli, M., Facco, M., Zonta, F., Brunati, A.M., Zambello, R., et al. (2014). Cortactin, another player in the Lyn signaling pathway, is over-expressed and alternatively spliced in leukemic cells from patients with B-cell chronic lymphocytic leukemia. Haematologica *99*, 1069–1077.

53. McCarthy, B.A., Yancopoulos, S., Tipping, M., Yan, X.J., Wang, X.P., Bennett, F., Li, W., Lesser, M., Paul, S., Boyle, E., et al. (2015). A seven-gene expression panel distinguishing clonal expansions of pre-leukemic and chronic lymphocytic leukemia B cells from normal B lymphocytes. Immunol. Res. *63*, 90–100.

54. Mittal, A.K., Chaturvedi, N.K., Rohlfsen, R.A., Gupta, P., Joshi, A.D., Hegde, G.V., Bociek, R.G., and Joshi, S.S. (2013). Role of CTLA4 in the proliferation and survival of chronic lymphocytic leukemia. PLoS One *8*, e70352.

55. Collins, A.V., Brodie, D.W., Gilbert, R.J.C., Iaboni, A., Manso-Sancho, R., Walse, B., Stuart, D.I., van der Merwe, P.A., and Davis, S.J. (2002). The interaction properties of costimulatory molecules revisited. Immunity *17*, 201–210.

56. Parry, R.V., Chemnitz, J.M., Frauwirth, K.A., Lanfranco, A.R., Braunstein, I., Kobayashi, S.V., Linsley, P.S., Thompson, C.B., and Riley, J.L. (2005). CTLA-4 and PD-1 receptors inhibit T-cell activation by distinct mechanisms. Mol. Cell Biol. *25*, 9543–9553.

57. Kulis, M., Heath, S., Bibikova, M., Queirós, A.C., Navarro, A., Clot, G., Martínez-Trillos, A., Castellano, G., Brun-Heath, I., Pinyol, M., et al. (2012). Epigenomic analysis detects widespread gene-body DNA hypomethylation in chronic lymphocytic leukemia. Nat. Genet. *44*, 1236–1242.

58. Yang, J.Y., Huo, Y.M., Yang, M.W., Shen, Y., Liu, D.J., Fu, X.L., Tao, L.Y., He, R.Z., Zhang, J.F., Hua, R., et al. (2021). SF3B1 mutation in pancreatic cancer contributes to aerobic glycolysis and tumor growth through a PP2A-c-Myc axis. Mol. Oncol. *15*, 3076–3090.

59. Rossi, D., Bruscaggin, A., Spina, V., Rasi, S., Khiabanian, H., Messina, M., Fangazio, M., Vaisitti, T., Monti, S., Chiaretti, S., et al. (2011). Mutations of the SF3B1 splicing factor in chronic lymphocytic leukemia: association with progression and fludarabine-refractoriness. Blood *118*, 6904–6908.

60. Cretu, C., Schmitzová, J., Ponce-Salvatierra, A., Dybkov, O., De Laurentiis, E.I., Sharma, K., Will, C.L., Urlaub, H., Lührmann, R., and Pena, V. (2016). Molecular Architecture of SF3b and Structural Consequences of Its Cancer-Related Mutations. Mol. Cell *64*, 307–319.

61. Zhou, Z., Gong, Q., Wang, Y., Li, M., Wang, L., Ding, H., and Li, P. (2020). The biological function and clinical significance of SF3B1 mutations in cancer. Biomark. Res. *8*, 38.

62. Prabakaran, S., Hemberg, M., Chauhan, R., Winter, D., Tweedie-Cullen, R.Y., Dittrich, C., Hong, E., Gunawardena, J., Steen, H., Kreiman, G., and Steen, J.A. (2014). Quantitative profiling of peptides from RNAs classified as noncoding. Nat. Commun. *5*, 5429.

63. Nusinow, D.P., Szpyt, J., Ghandi, M., Rose, C.M., McDonald, E.R., 3rd, Kalocsay, M., Jané-Valbuena, J., Gelfand, E., Schweppe, D.K., Jedrychowski, M., et al. (2020). Quantitative Proteomics of the Cancer Cell Line Encyclopedia. Cell *180*, 387–402.e16.

64. Zheng, J.T., Lin, C.X., Fang, Z.Y., and Li, H.D. (2020). Intron Retention as a Mode for RNA-Seq Data Analysis. Front. Genet. *11*, 586.

65. Lappalainen, I., Almeida-King, J., Kumanduri, V., Senf, A., Spalding, J.D., Ur-Rehman, S., Saunders, G., Kandasamy, J., Caccamo, M., Leinonen, R., et al. (2015). The European Genome-phenome Archive of human data consented for biomedical research. Nat. Genet. *47*, 692–695.

66. Li, H., Handsaker, B., Wysoker, A., Fennell, T., Ruan, J., Homer, N., Marth, G., Abecasis, G., and Durbin, R.; 1000 Genome Project Data Processing Subgroup (2009). The Sequence Alignment/Map format and SAMtools. Bioinformatics *25*, 2078–2079.

67. Trapnell, C., Pachter, L., and Salzberg, S.L. (2009). TopHat: discovering splice junctions with RNA-Seq. Bioinformatics *25*, 1105–1111.

68. Anders, S., Reyes, A., and Huber, W. (2012). Detecting differential usage of exons from RNA-seq data. Genome Res. *22*, 2008–2017.

69. Frazee, A.C., Pertea, G., Jaffe, A.E., Langmead, B., Salzberg, S.L., and Leek, J.T. (2015). Ballgown bridges the gap between transcriptome assembly and expression analysis. Nat. Biotechnol. *33*, 243–246.

70. Vera Alvarez, R., Pongor, L.S., Mariño-Ramírez, L., and Landsman, D. (2019). TPMCalculator: one-step software to quantify mRNA abundance of genomic features. Bioinformatics *35*, 1960–1962.

71. Mi, H., Lazareva-Ulitsky, B., Loo, R., Kejariwal, A., Vandergriff, J., Rabkin, S., Guo, N., Muruganujan, A., Doremieux, O., Campbell, M.J., et al. (2005). The PANTHER database of protein families, subfamilies, functions and pathways. Nucleic Acids Res. *33*, D284–D288.

72. Mi, H., Poudel, S., Muruganujan, A., Casagrande, J.T., and Thomas, P.D. (2016). PANTHER version 10: expanded protein families and functions, and analysis tools. Nucleic Acids Res. *44*, D336–D342.

73. Wang, J., Duncan, D., Shi, Z., and Zhang, B. (2013). WEB-based GEne SeT AnaLysis Toolkit (WebGestalt): update 2013. Nucleic Acids Res. *41*, W77–W83.

74. Fabregat, A., Sidiropoulos, K., Garapati, P., Gillespie, M., Hausmann, K., Haw, R., Jassal, B., Jupe, S., Korninger, F., McKay, S., et al. (2016). The Reactome pathway Knowledgebase. Nucleic Acids Res. *44*, D481–D487.

75. Chuang, H.Y., Rassenti, L., Salcedo, M., Licon, K., Kohlmann, A., Haferlach, T., Foà, R., Ideker, T., and Kipps, T.J. (2012). Subnetwork-based analysis of chronic lymphocytic leukemia identifies pathways that associate with disease progression. Blood *120*, 2639–2649.

76. Cline, M.S., Smoot, M., Cerami, E., Kuchinsky, A., Landys, N., Workman, C., Christmas, R., Avila-Campilo, I., Creech, M., Gross, B., et al. (2007). Integration of biological networks and gene expression data using Cytoscape. Nat. Protoc. *2*, 2366–2382.

77. Rassenti, L.Z., Huynh, L., Toy, T.L., Chen, L., Keating, M.J., Gribben, J.G., Neuberg, D.S., Flinn, I.W., Rai, K.R., Byrd, J.C., et al. (2004). ZAP-70 compared with immunoglobulin heavy-chain gene mutation status as a predictor of disease progression in chronic lymphocytic leukemia. N. Engl. J. Med. *351*, 893–901.

78. WMA (2000). World Medical Association Declaration of Helsinki: ethical principles for medical research involving human subjects. JAMA *284*, 3043–3045.

79. Lumb, S., Fleischer, S.J., Wiedemann, A., Daridon, C., Maloney, A., Shock, A., and Dörner, T. (2016). Engagement of CD22 on B cells with the monoclonal antibody epratuzumab stimulates the phosphorylation of upstream inhibitory signals of the B cell receptor. J. Cell Commun. Signal. *10*, 143–151.

80. Talab, F., Allen, J.C., Thompson, V., Lin, K., and Slupsky, J.R. (2013). LCK is an important mediator of B-cell receptor signaling in chronic lymphocytic leukemia cells. Mol. Cancer Res. *11*, 541–554.

OMTN, Volume *35*

Supplemental information

Aberrant spliceosome activity via elevated intron retention and upregulation and phosphorylation of SF3B1 in chronic lymphocytic leukemia

Manoj Kumar Kashyap, Hiren Karathia, Deepak Kumar, Roberto Vera Alvarez, Jose Vicente Forero-Forero, Eider Moreno, Juliana Velez Lujan, Carlos Ivan Amaya-Chanaga, Newton Medeiros Vidal, Zhe Yu, Emanuela M. Ghia, Paula A. Lengerke-Diaz, Daniel Achinko, Michael Y. Choi, Laura Z. Rassenti, Leonardo Mariño-Ramírez, Stephen M. Mount, Sridhar Hannenhalli, Thomas J. Kipps, and Januario E. Castro

Supplemental Material

Detailed information required to perform the intron/exon quantification as we did in this work

Our assumption was if the expression of a distinct transcript *i* in sample *j* differs from the expected value for an experimental condition (i.e., normal or cancer), the values *μijl* for all of the counting bins *l* of transcript *i* will deviate from the values expected for condition in normal or CLL by the same factor. Hence, we consider the bin (*l*) as length of exonic and intronic region of a transcript defined in standard annotation of hg19 NCBI Refseq.

Formulas used to operate the *in-house* program designed to calculate TPM values for every exonic and intronic region of the expressed transcripts

The TPM calculations were performed separately for every exon, intron and transcript in each sample (CLL and NBC). To capture single value of intron and exon per transcript in a sample, we first calculated $\max(\forall TPM_{exon})$ and $\max(\forall TPM_{intron})$ for each transcript and then took $mean[\max(\forall TPM_{exon})]$ and $mean[\max(\forall TPM_{intron})]$ across NBC and CLL samples, which we termed respectively for intron-usage and exon-usage as defined as $TPM\_NBC\_Intronic$, TPM_NBC_Exonic, $TPM\_CLL\_Intronic$ and TPM_CLL_Exonic (Equation S1, S2, and S3). [1]

$$TPM_{i=} \frac{r_i * 10^6}{l_i \sum_{j=1}^{N} \frac{r_i}{l_j}}$$ **Equation - S1**

$$TPMexon\_i = 10^6 * \frac{\frac{NEi}{LEi}}{\sum_{j=0}^{j=n} \left(\frac{NEj}{LEj}\right)}$$ **Equation - S2**

$$TPMintron\_i = 10^6 * \frac{\frac{NIi}{LIi}}{\sum_{j=0}^{j=n} \left(\frac{NIj}{LIj}\right)}$$ **Equation - S3**

where *N*=Transcript, *NE*=Exonic region, *NI*=Intronic region, *LE*=Length of the exonic region and *LI*=Length of intronic region.

In order to quantify relative intron-usage and exon-usage for each transcript in NBC and CLL, we took two combinations of ratios. Firstly, two separated ratios of $TPM\_Intronic$ and TPM_Exonic in between CLL and NBC; we call these ratios as Log2_intronic and Log2_exonic respectively (Equation S4 & S5). Secondly, the same way the two separate ratios; one for $TPM\_Intronic$ and TPM_Exonic in CLL and the same in NBC.

$$Log2Intronic_i = log_2\left(\frac{TPM\_CLL\_Intronic_i}{TPM\_NBC\_Intronic_i}\right) \qquad \textbf{Equation – S4}$$

$$Log2Exonic_i = log_2\left(\frac{TPM\_CLL\_Exonic_i}{TPM\ NBC\ Exonic_i}\right) \qquad \textbf{Equation – S5}$$

Equations (S4 & S5) were used for comparing the similar entity between experimental conditions, which was CLL versus NBC cells in this case. By doing so, TPM value of the exonic regions can be related to the expression level of the isoform in the experiments, an isoform with a positive value of equation S4 can be read as more expression of those exonic or intronic regions in the CLL compared to NBC and vice versa if the value is negative.

Supplemental Tables

* **Table S1.** List of Chronic Lymphocytic Leukemia Patients and Normal Subject Used in the study

* **Table S2.** List of transcripts with IR in Chronic Lymphocytic Leukemia (CLL)-B Cells and Normal B Cells (NBCs)

* Please refer to the "**Supplemental Videos and Spreadsheets**"

Table S3. List of primers for detection of SF3B1 mutations in CLL Samples[2]

SF3B1	Primer Direction	Primer Sequence	PCR Product (BPs)
Exon 14	Forward	5’ TCTGTTTATGGAATTGATTATGGAA 3’	424
Exon 14	Reverse	3’ GGGCAACATAGTAAGACCCTGT 5’	
Exon 15	Forward	5’ TTGGGGCATAGTTAAAACCTG 3’	209
Exon 15	Reverse	3’ AAATCAAAAGGTAATTGGTGGA 5’	

Table S4. *SF3B1* Mutational status of CLL patients used in RNA-seq Study

S. NO.	Sample ID	Type of *SF3B1* Mutation
1	CLL009	MUT
2	CLL019	MUT
3	CLL029	MUT
4	CLL053	MUT
5	CLL154	MUT
6	CLL156	MUT
7	CLL182	MUT
8	CLL197	MUT
9	CLL306	MUT

Table S5. Primers used for other genes validated using RT-PCR

Name of Molecule (Gene Symbol)	Refseq ID	Primers	Primer Sequence [5' -----→ 3']	Amplicon Size (bp)
THAP8	NM_152658	FP	GATGGGGAGAGCCAAGACTTC	213
		RP	GTTTGTGTCCCGGAAGAATGG	
PPP2R5B	NM_006244	FP (Set-1A)	ATTAGACTCGCTTTGGGATGC	104
		RP (Set-1A)	GCCCATAAGAACGGGAGGAAG	
		FP (Set-1B)	CATTAGACTCGCTTTGGGATGC	106
		RP (Set-1B)	GGCCCATAAGAACGGGAGGA	
PSTPIP1	NM_003978	FP	GTGGAAACCAAGACTGCCTCT	135
		RP	GGAGGATGGAGCATGACTGA	
PTPRJ	NM_001098503	FP	TTCAACCCGTGGACTTTGGT	177
		RP	TGCCCCCACGATGTTGATTT	
NUBP2	NM_001284502	FP	CCTGGCTCCGTGTTCTGATT	178
		RP	TCCATCAGGTGTCACTTGCC	

FP: Forward primer, RP: Reverse primer, THAP8: THAP domain containing 8, PPP2R5B: Protein phosphatase 2 regulatory subunit B'beta, PSTPIP1: Proline-serine-threonine phosphatase interacting protein 1, PTPRJ: *Protein tyrosine phosphatase receptor type J*, *NUMBP2*: NUBP iron-sulfur cluster assembly factor 2, cytosolic

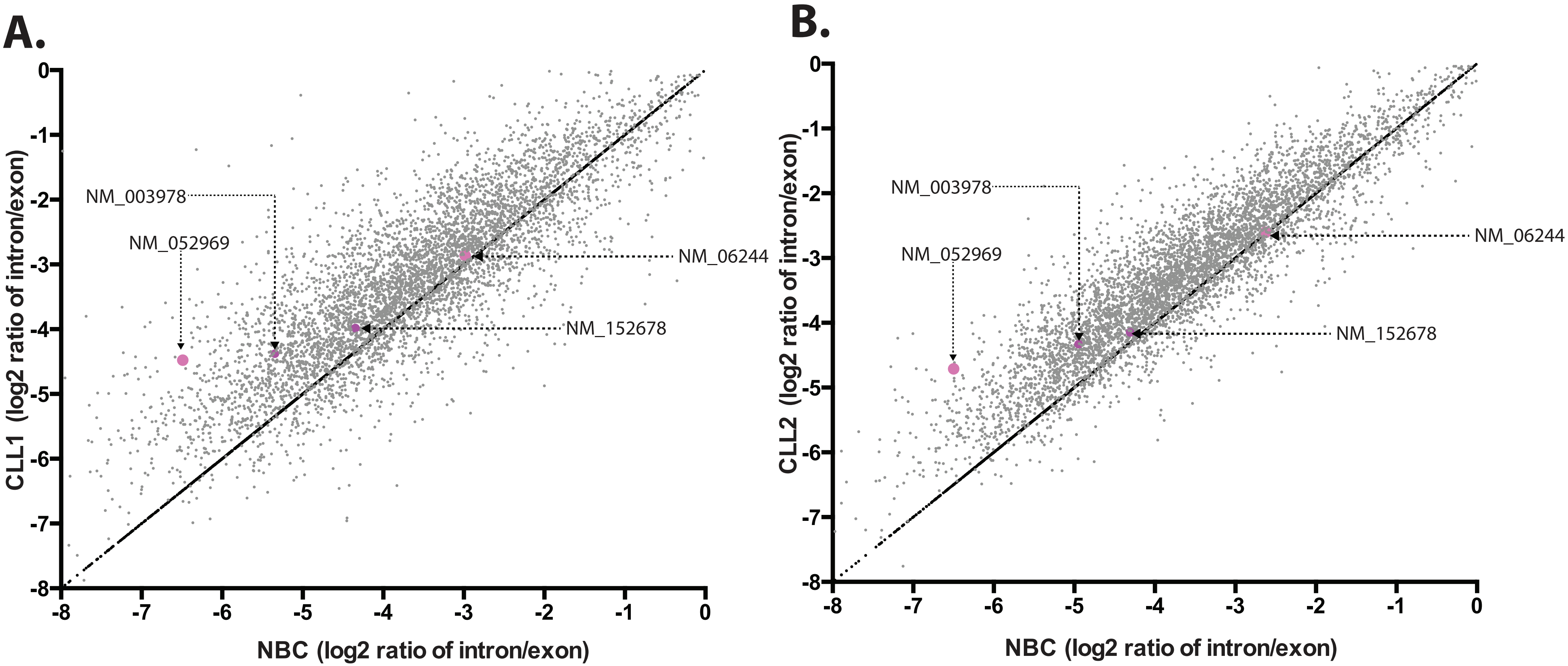

Figure S1: Intron/Exon ratio in CLL vs. NBCs

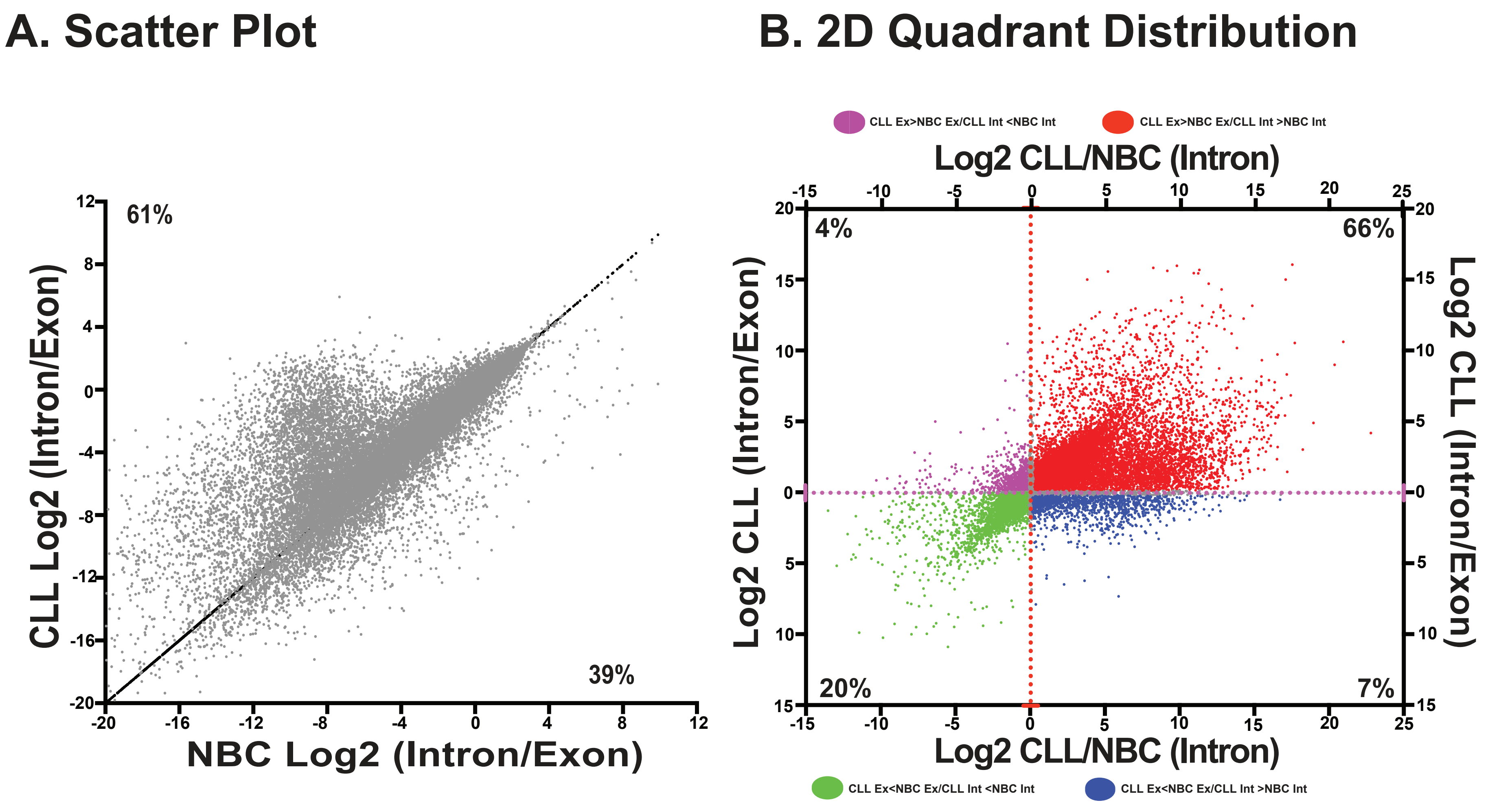

Figure S2. Global distribution of I/E Between CLL vs NBC

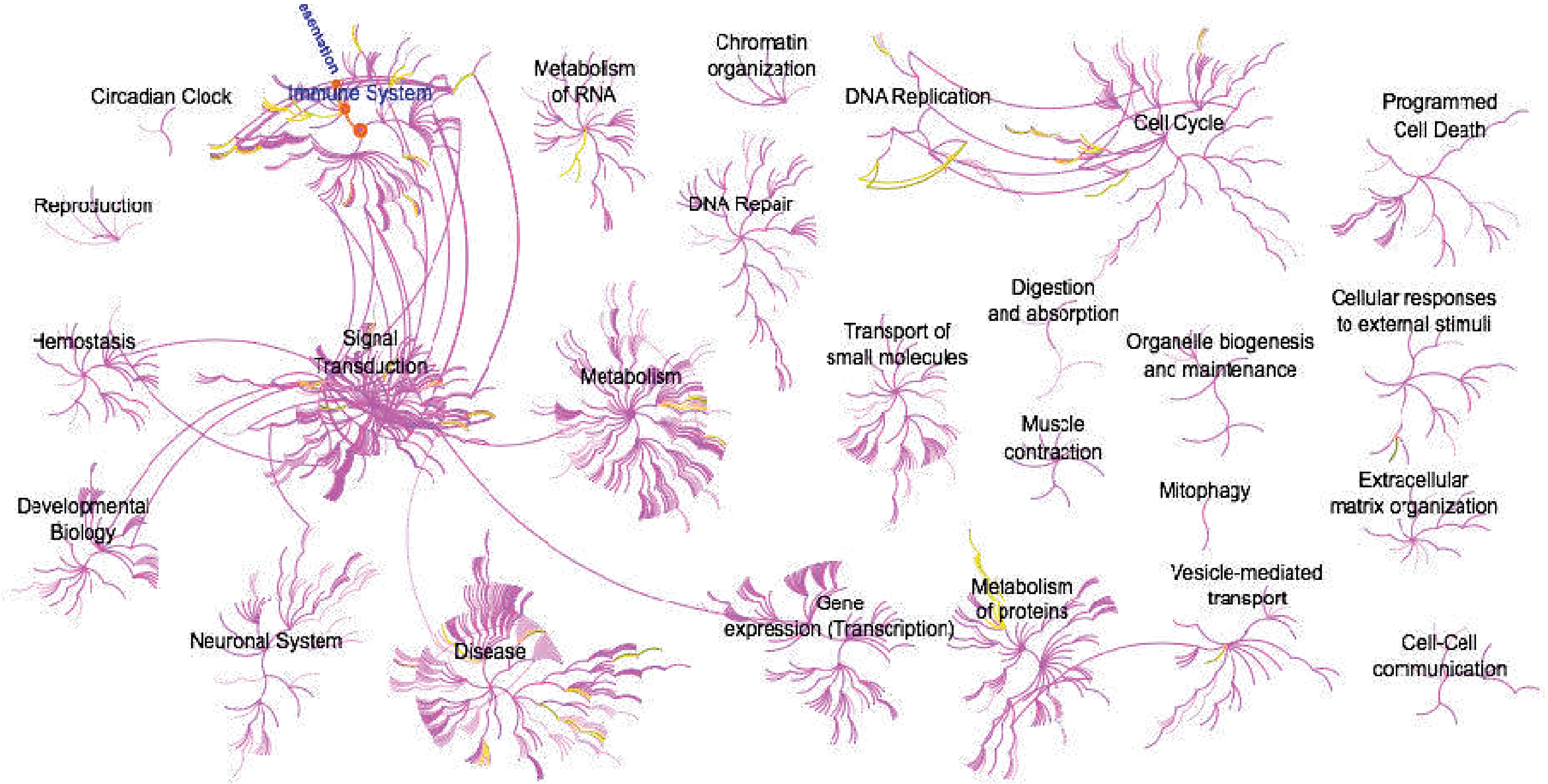

Figure S3. Emergence of major spliceosome pathwyas with higher intron retention possessing transcripts in Normal B Cells

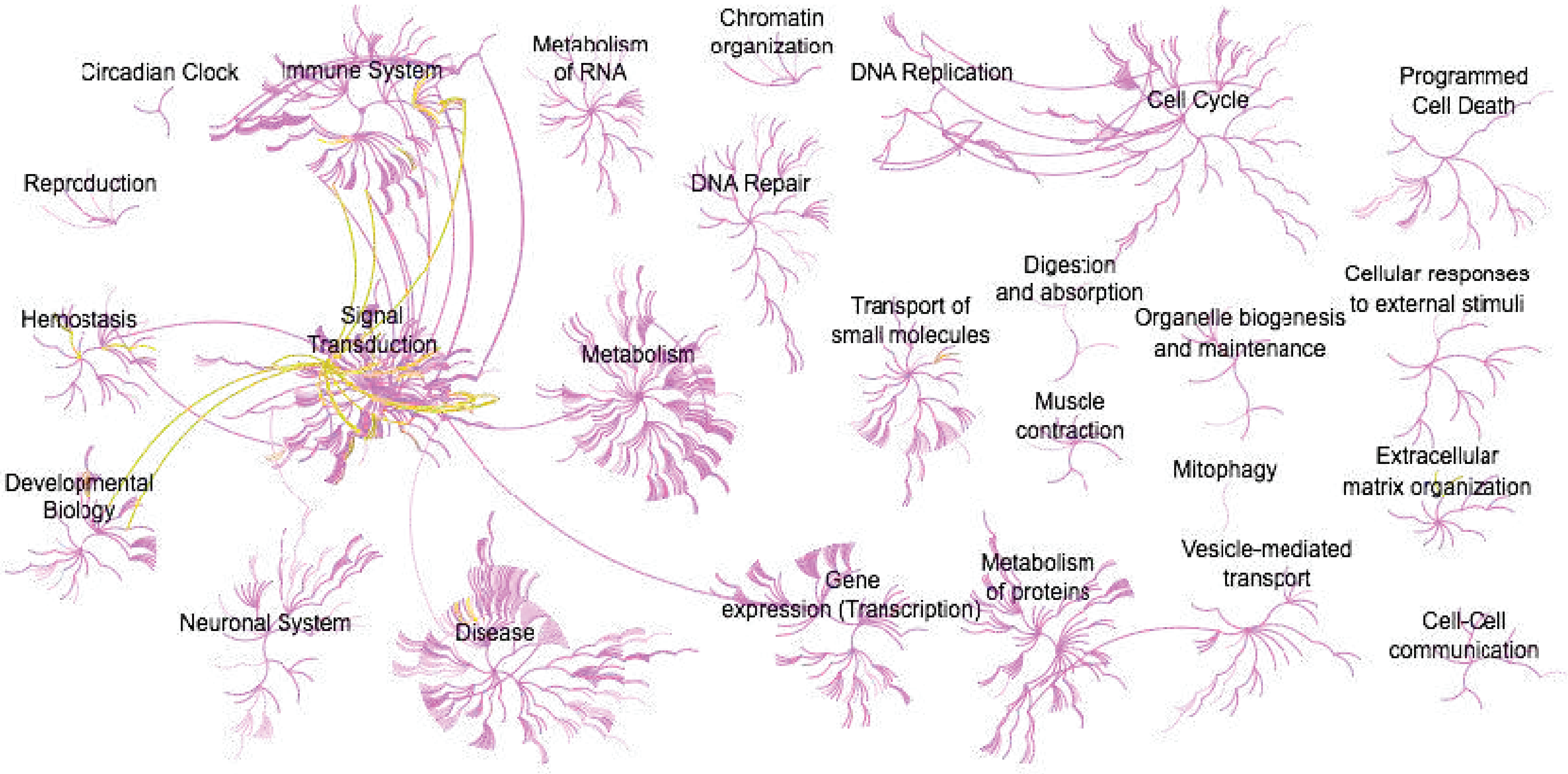

Figure S4. Emergence of major spliceosome pathwyas with higher intron retention possessing transcripts in CLL

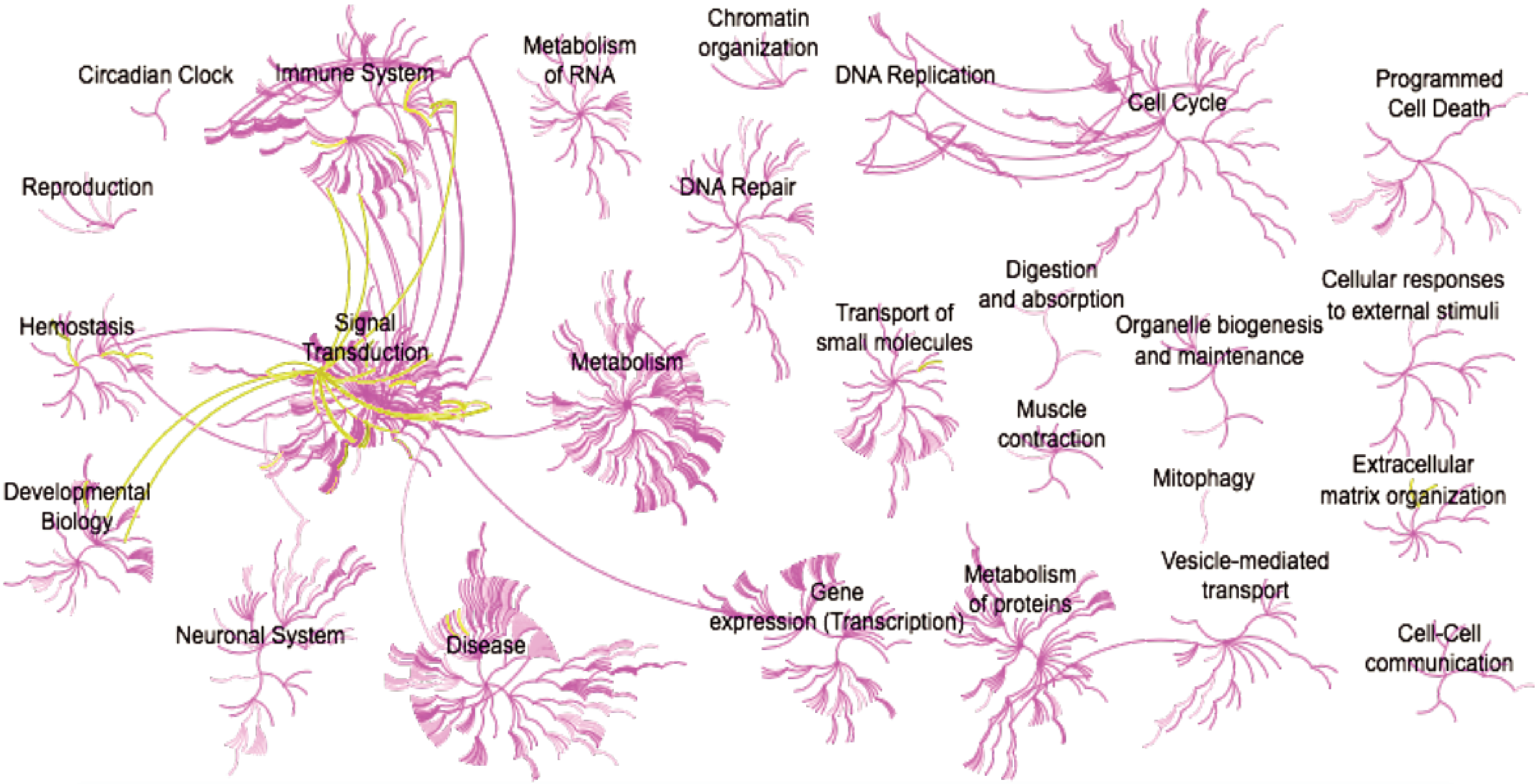

Figure S5. Emergence of major spliceosome pathwyas with higher intron retention possessing transcripts in CLL upon superimposing on NBC

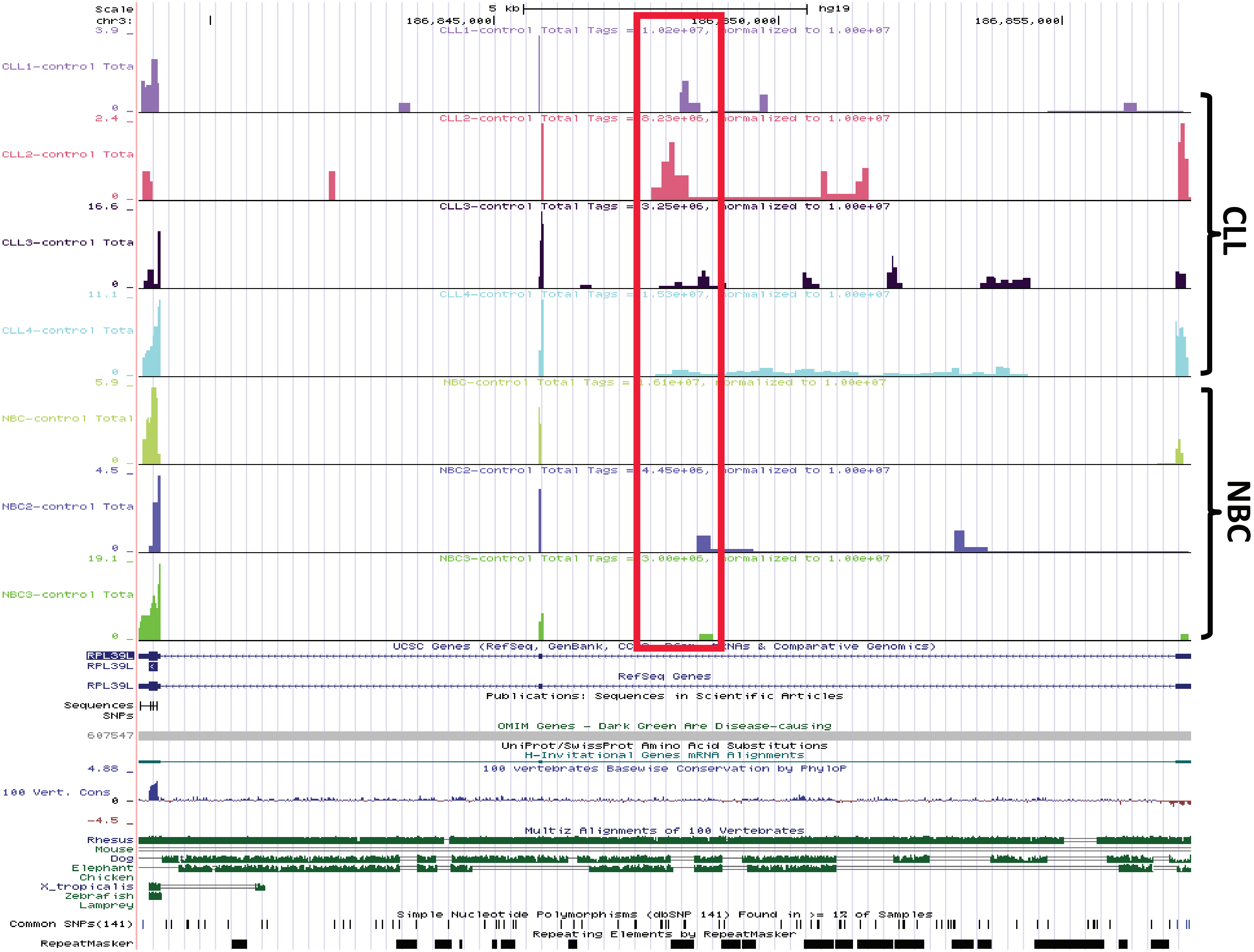

Figure S6A. Tracks of R*PL39L* gene showing intron usage in CLL vs. NBC

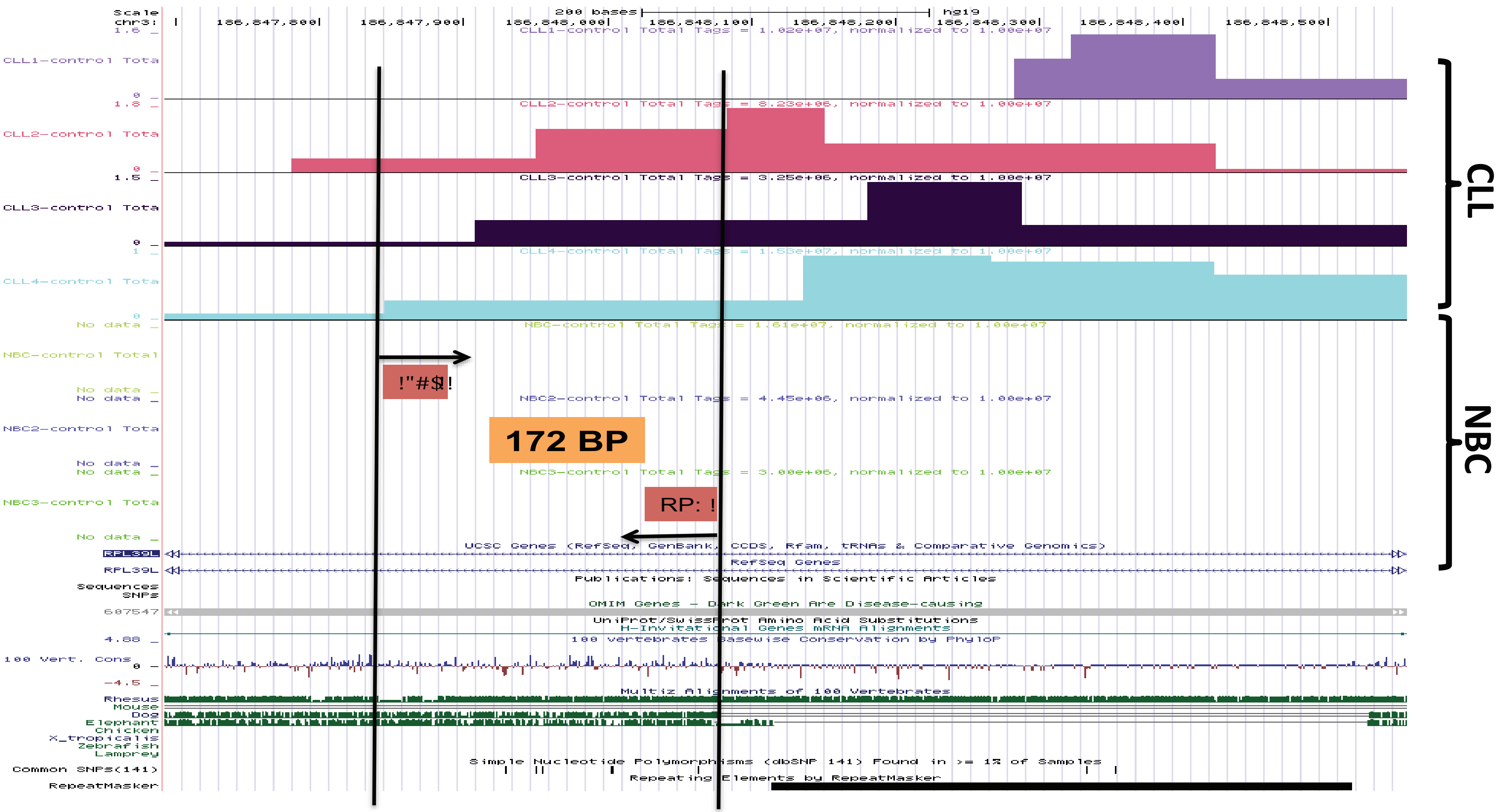

Figure S6B. Tracks of *RPL39L* gene showing intron usage in CLL vs. NBC

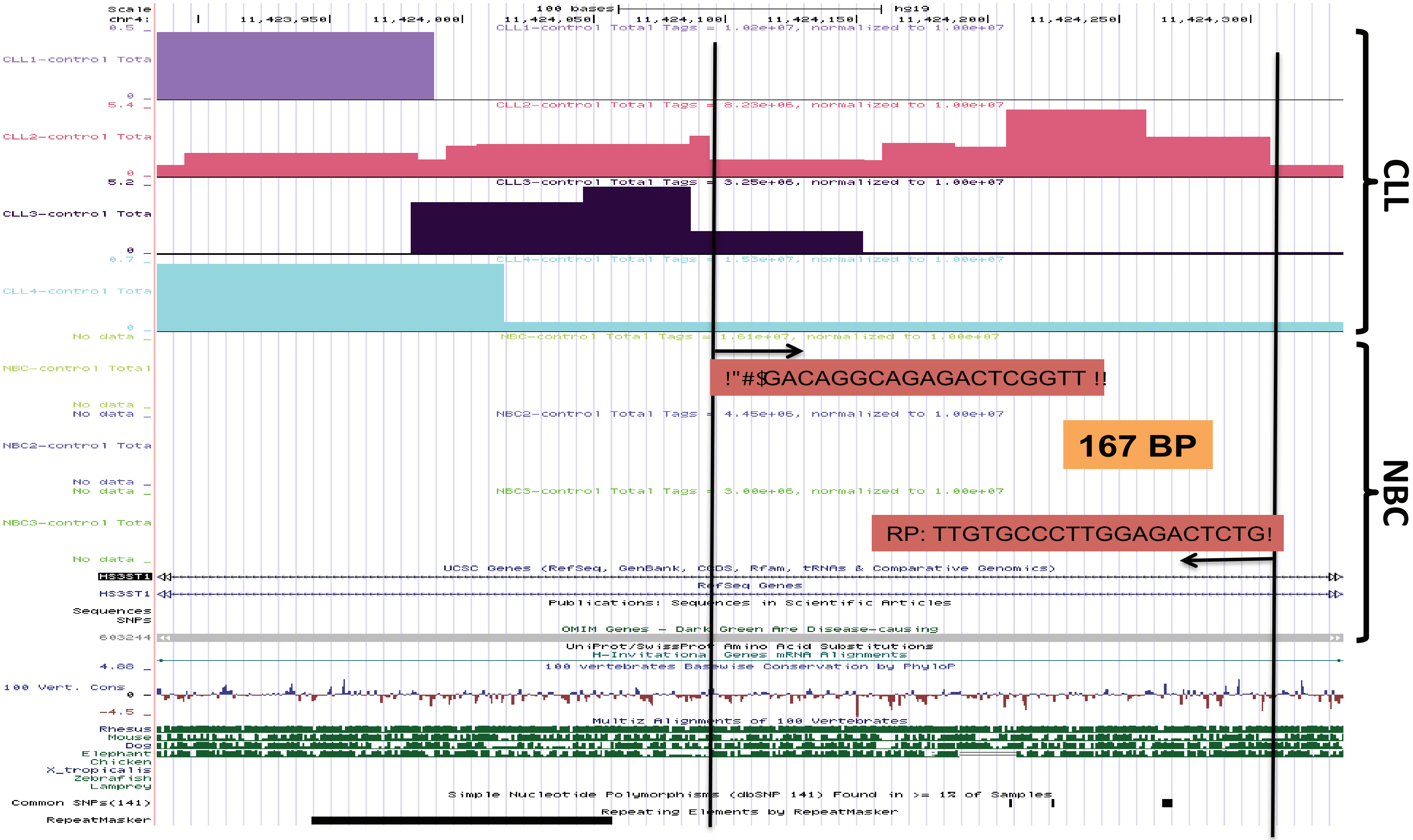

Figure S7A. Tracks of *HS3ST1* gene showing intron usage in CLL vs. NBC

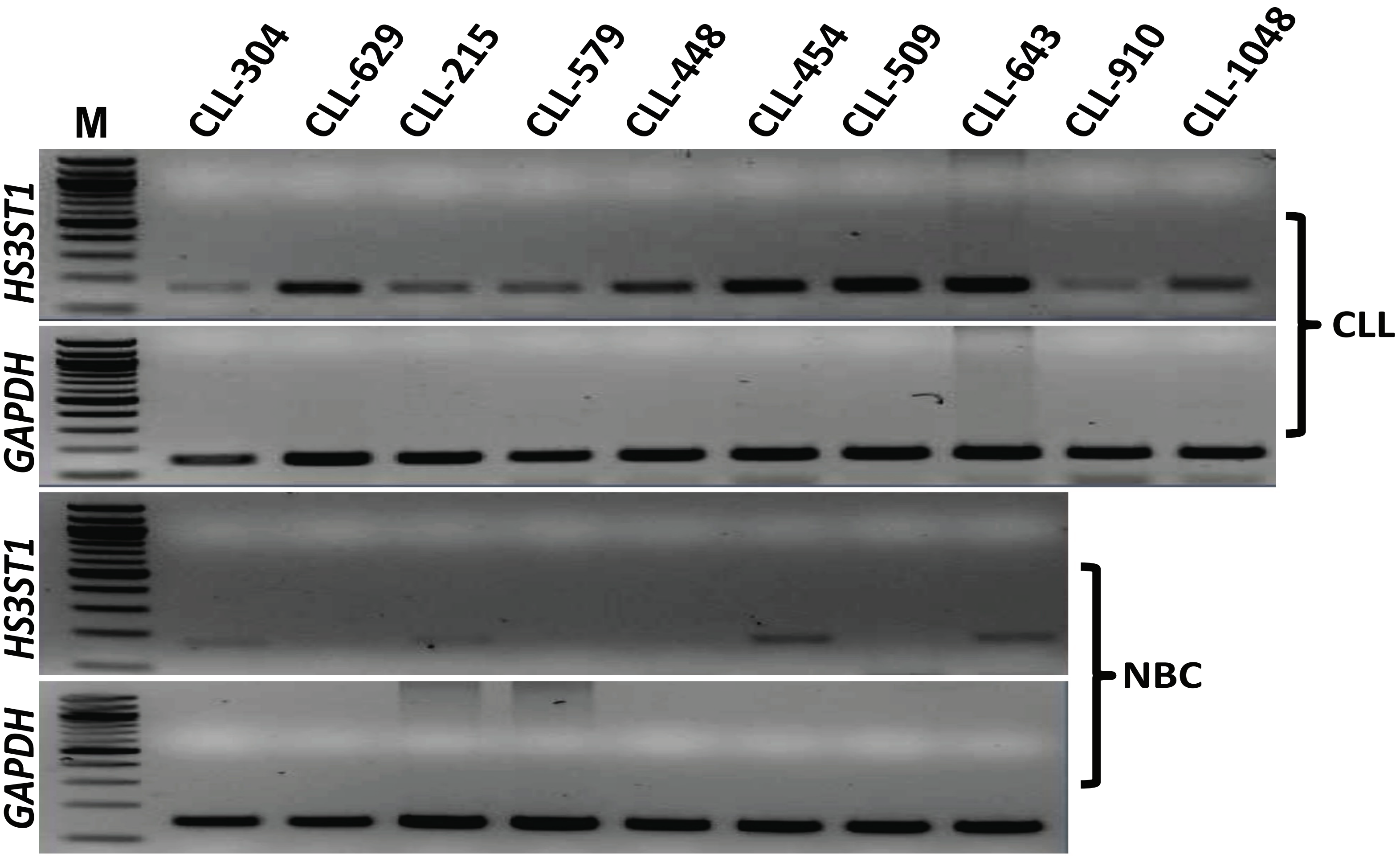

Figure S7B. RT-PCR of *HS3ST1* showing expression between CLL vs. NBC

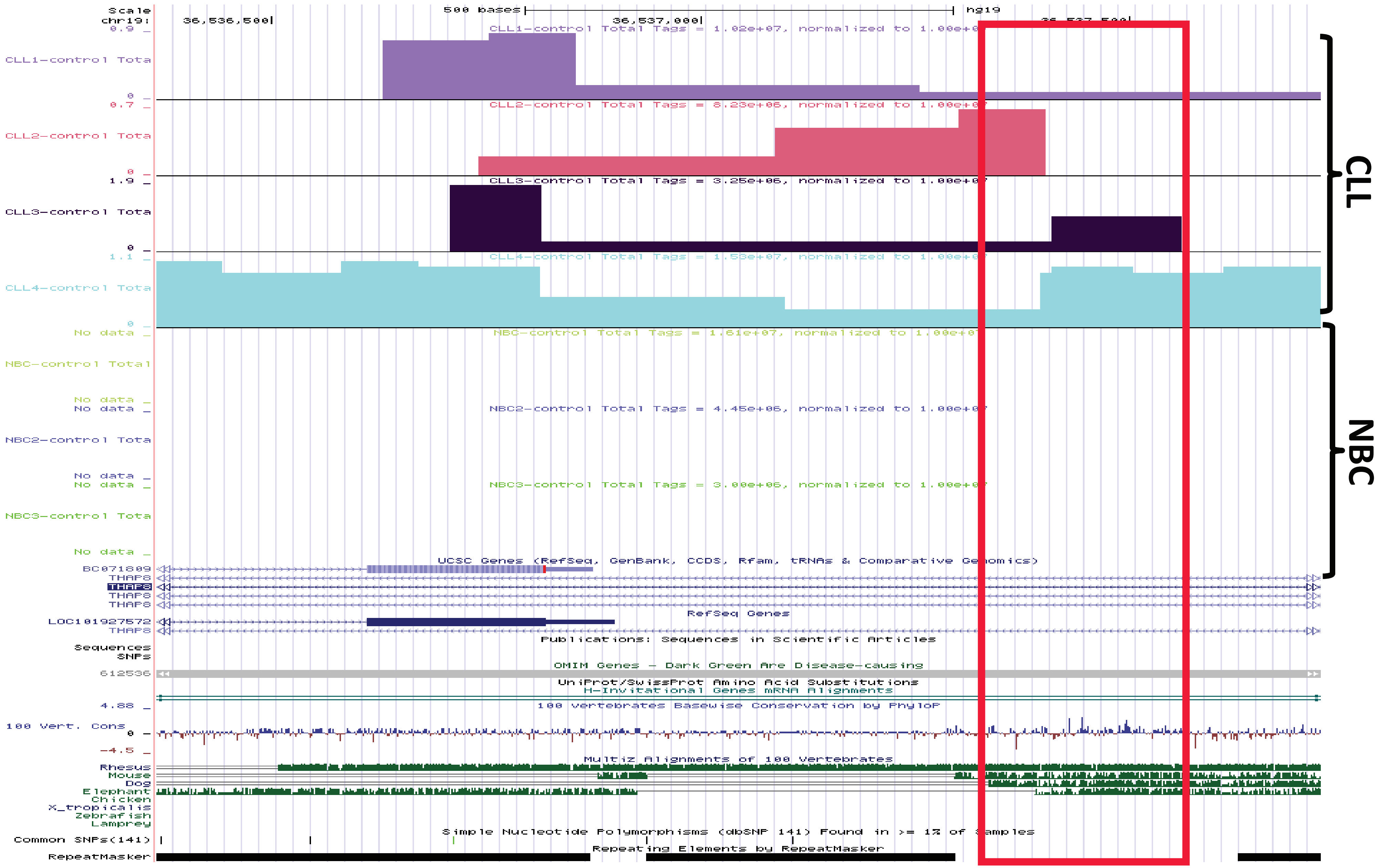

Figure S8A. Tracks of *THAP8* gene showing intron usage in CLL vs. NBC

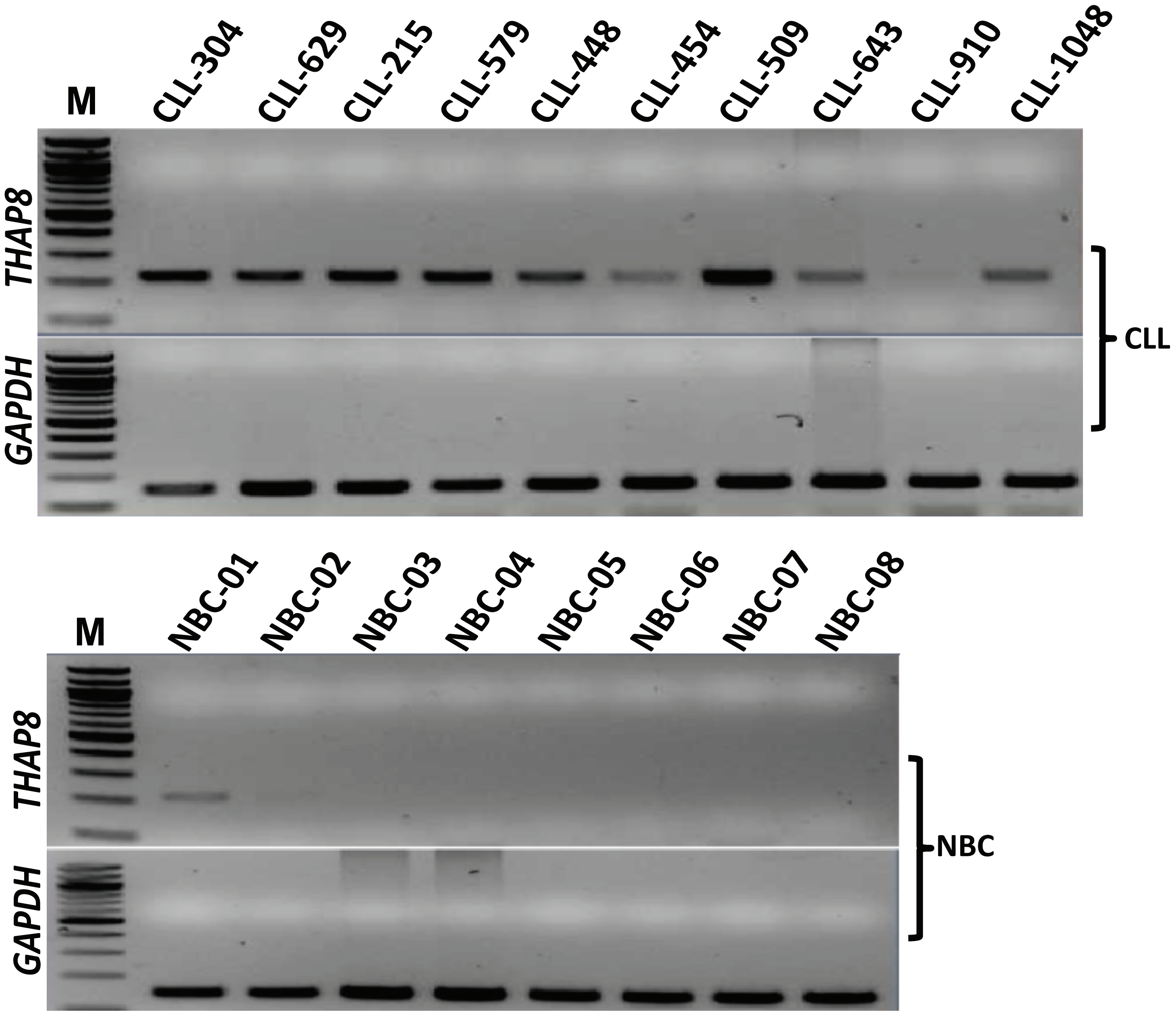

Figure S8B. RT-PCR of *THAP8* showing expression between CLL vs. NBC

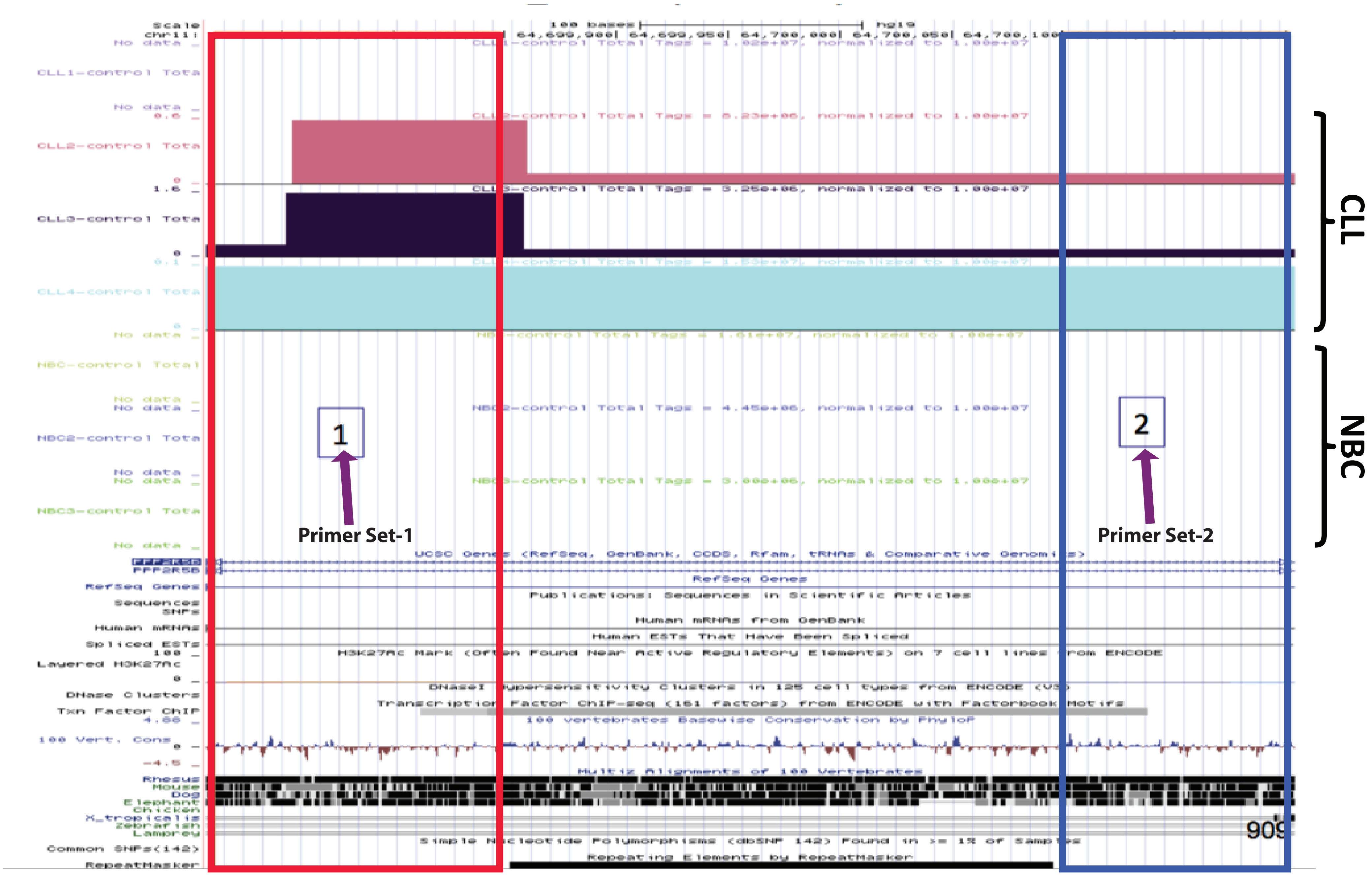

Figure S9A. Tracks of *PPP2R5B* gene showing intron usage in CLL vs. NBC

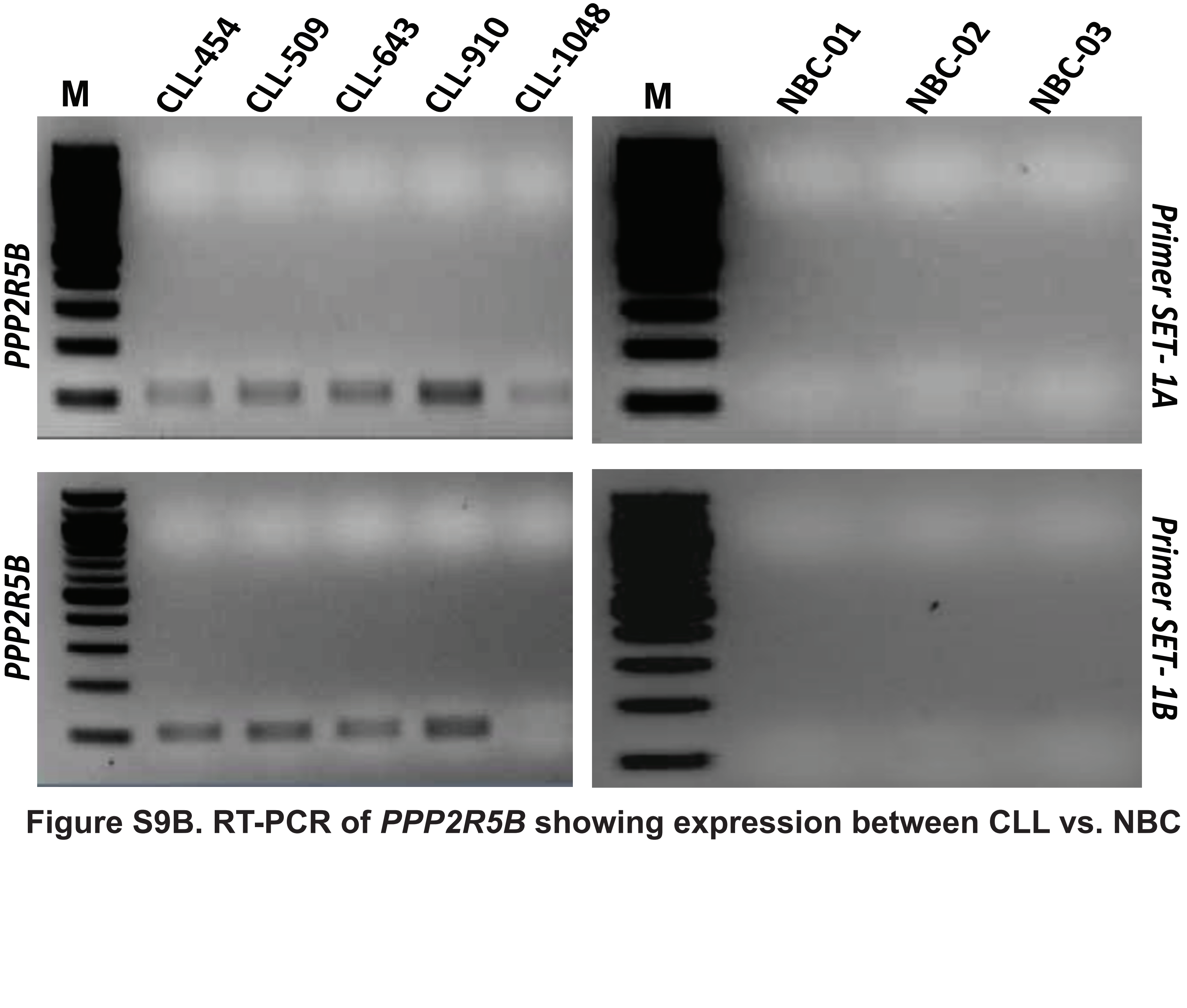

Figure S9B. RT-PCR of *PPP2R5B* showing expression between CLL vs. NBC

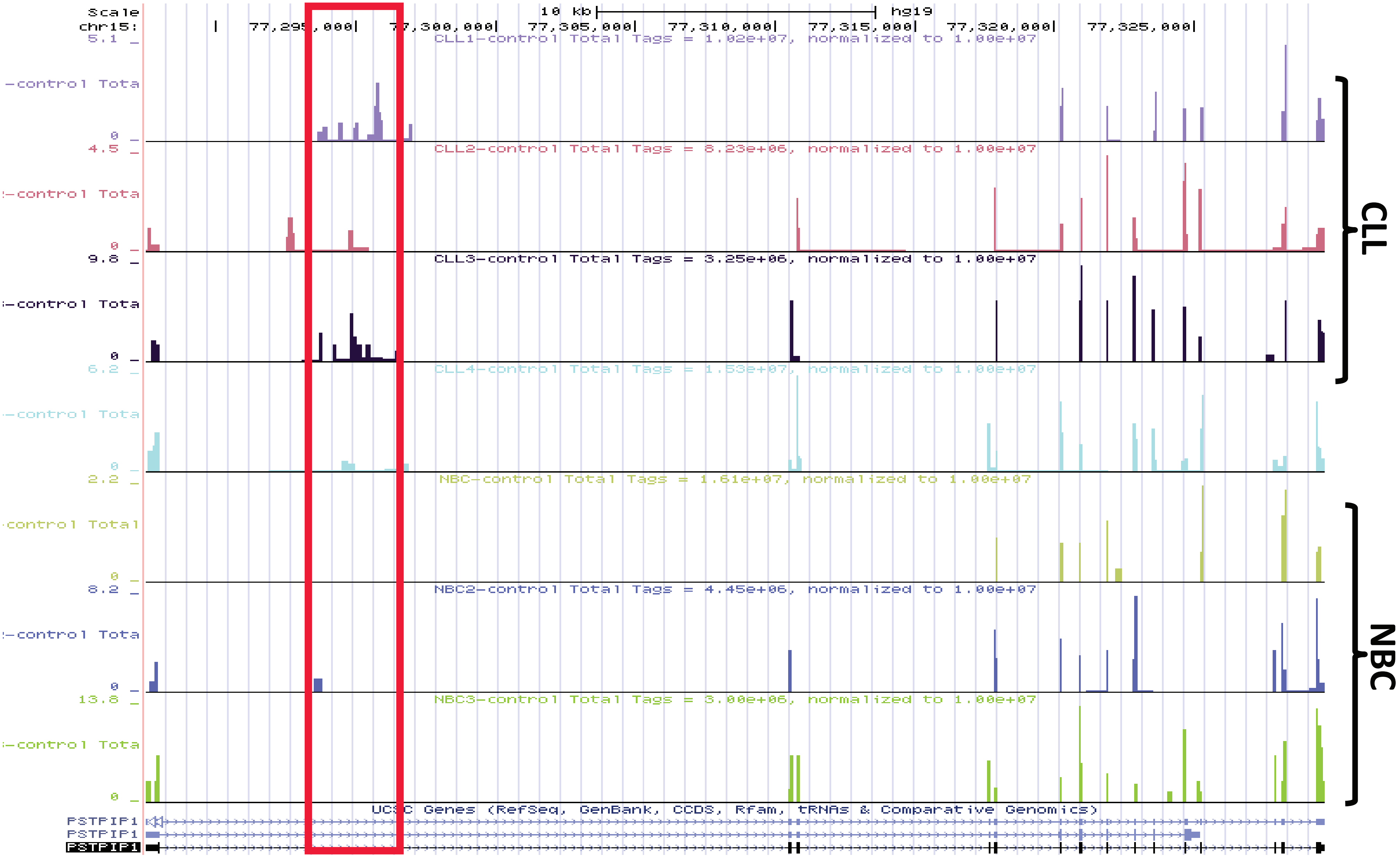

Figure S10A. Tracks of *PSTPIP1* gene showing intron usage in CLL vs. NBC

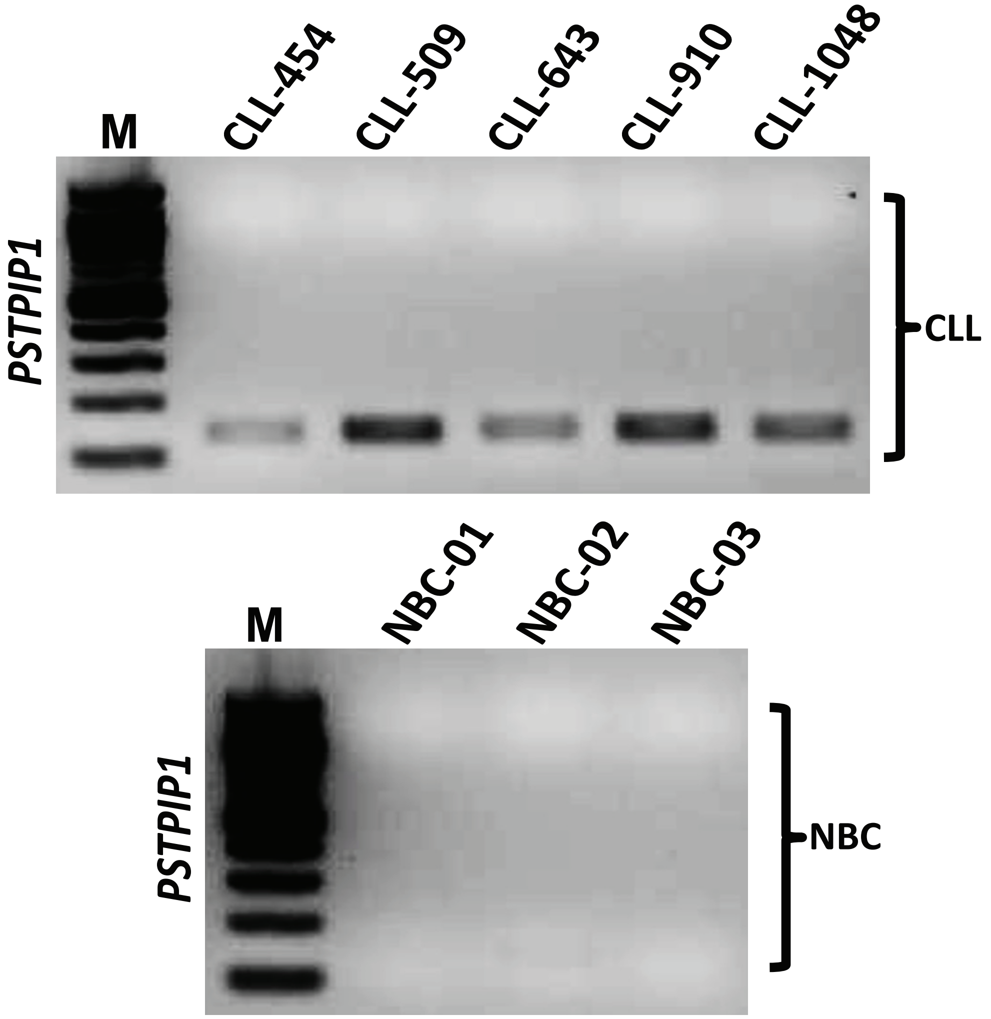

Figure 10B. RT-PCR of *PSTPIP1* showing expression between CLL vs. NBC

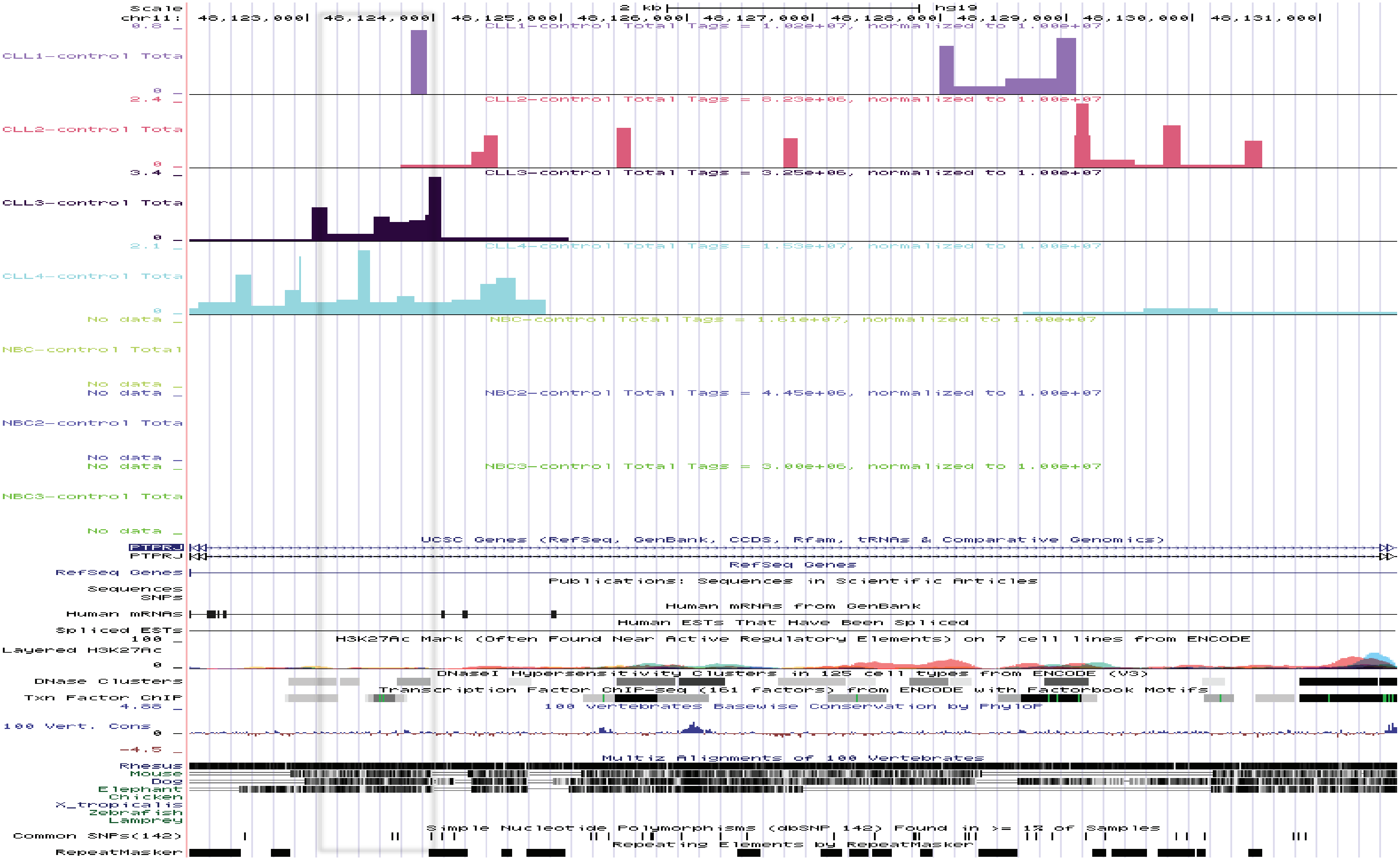

Figure S11A. Tracks of *PTPRJ* gene showing intron usage in CLL vs. NBC

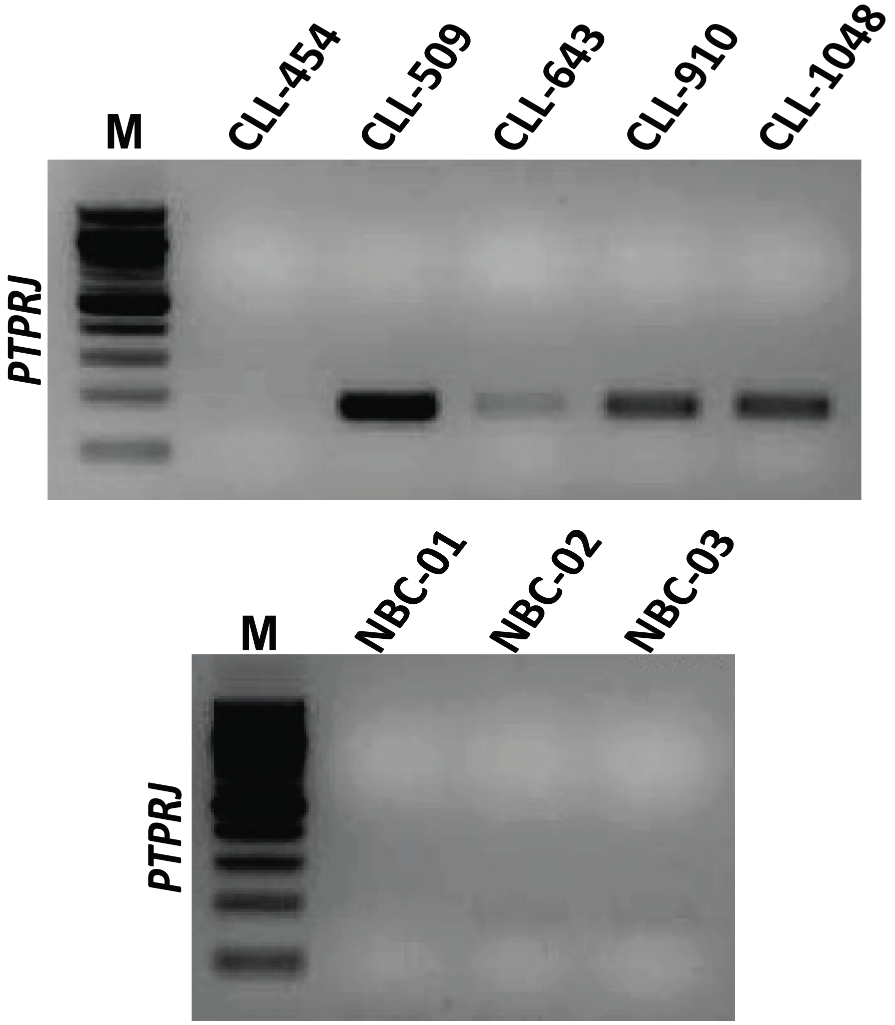

Figure 11B. RT-PCR of *PTPRJ* showing expression between CLL vs. NBC

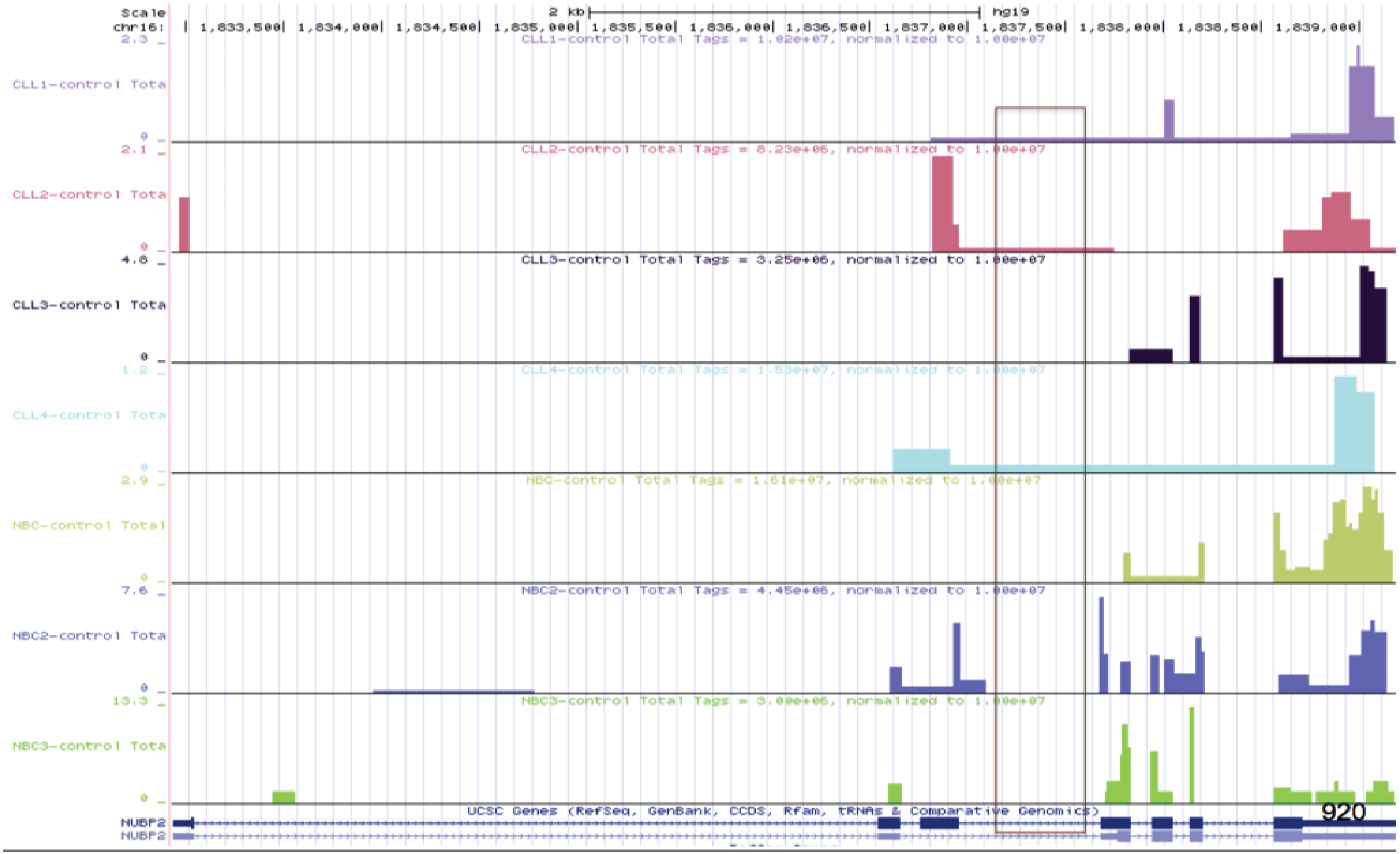

Figure S12A. Tracks of *NUBP2* gene showing intron usage in CLL vs. NBC

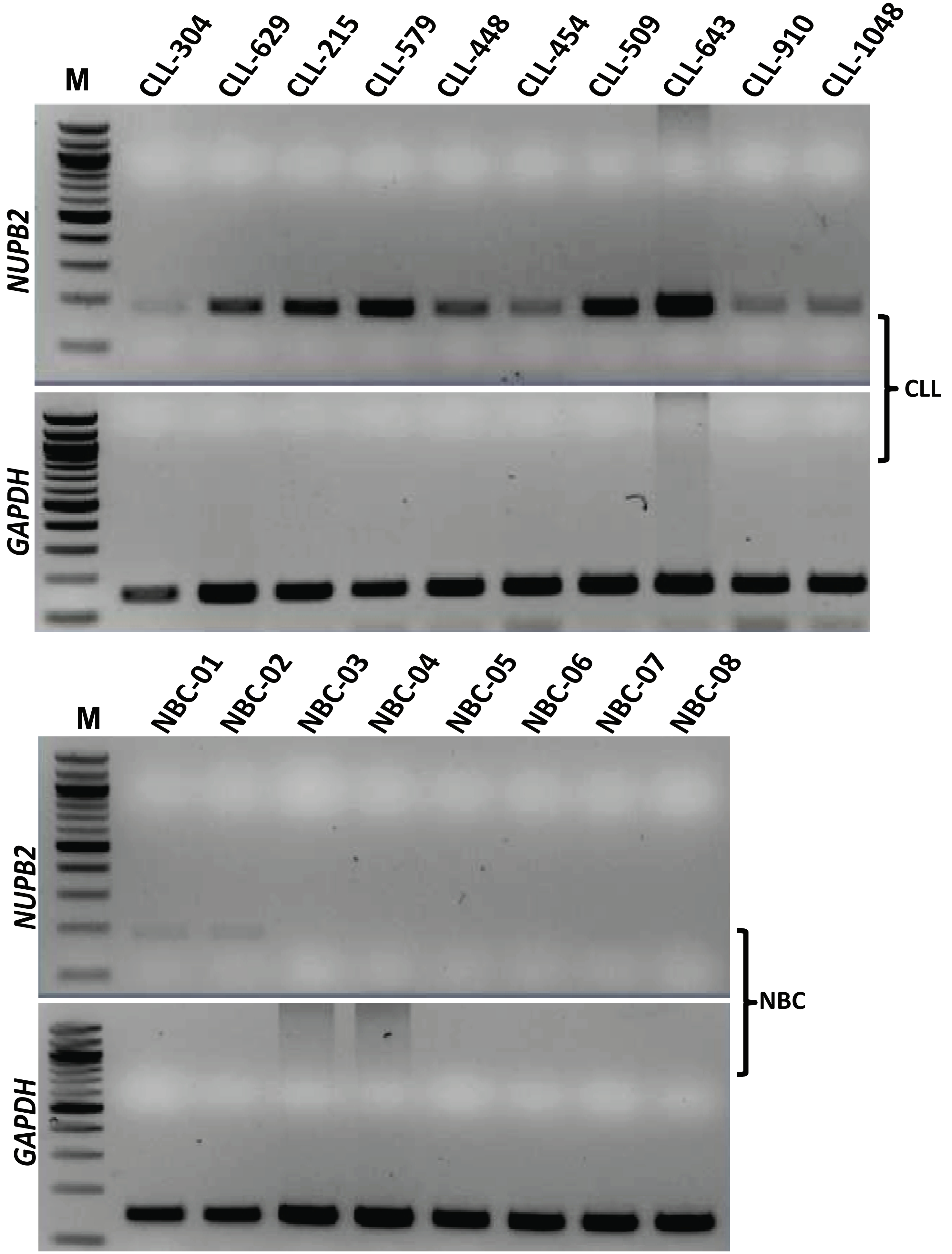

Figure S12B. RT-PCR of *NUPB2* showing expression between CLL vs. NBC

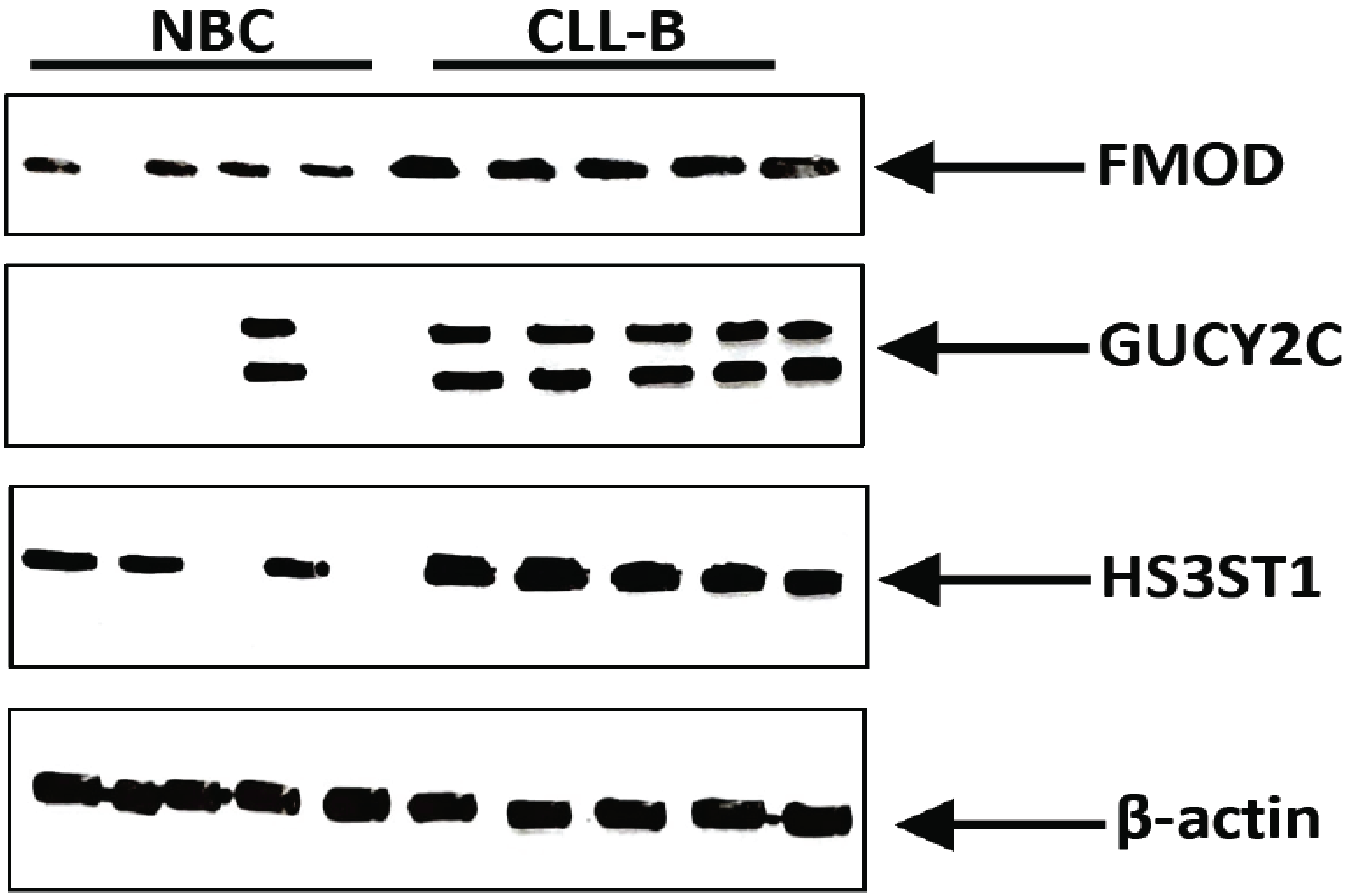

Figure S13: Validation of FMOD, GUCY2C, and HS3ST1 Using Western Blot Technique in NBC and CLL-B Cells

References

1. Vera Alvarez, R, Pongor, LS, Marino-Ramirez, L, and Landsman, D (2019). TPMCalculator: one-step software to quantify mRNA abundance of genomic features. *Bioinformatics* **35**: 1960-1962.

2. Schwaederle, M, Ghia, E, Rassenti, LZ, Obara, M, Dell'Aquila, ML, Fecteau, JF, *et al.* (2013). Subclonal evolution involving SF3B1 mutations in chronic lymphocytic leukemia. *Leukemia* **27**: 1214-1217.
